# *Clostridium butyricum* Protects Against Pancreatic and Intestinal Injury After Severe Acute Pancreatitis *via* Downregulation of MMP9

**DOI:** 10.3389/fphar.2022.919010

**Published:** 2022-07-18

**Authors:** Qingqing Yan, Lin Jia, Biyan Wen, Yao Wu, Yanbo Zeng, Qing Wang

**Affiliations:** ^1^ Department of Gastroenterology, Guangzhou First People’s Hospital, South China University of Technology, Guangzhou, China; ^2^ Department of Gastroenterology, The Second Affiliated Hospital, School of Medicine, South China University of Technology, Guangzhou, China; ^3^ Department of Gastroenterology, The First Affiliated Hospital of Nan Chang University, Nanchang, China; ^4^ Department of Gastroenterology, Changhai Hospital, Shanghai, China

**Keywords:** severe acute pancreatitis, *Clostridium butyricum*, gut microbiota, matrix metallopeptidase 9, intestinal injury

## Abstract

**Background:** Evidence have shown that gut microbiota plays an important role in the development of severe acute pancreatitis (SAP). In addition, matrix metalloproteinase-9 (MMP9) plays an important role in intestinal injury in SAP. Thus, we aimed to determine whether gut microbiota could regulate the intestinal injury during SAP *via* modulating MMP9.

**Methods:** In this study, the fecal samples of patients with SAP (*n* = 72) and healthy controls (*n* = 32) were analyzed by 16S rRNA gene sequencing. In addition, to investigate the association between gut microbiota and MMP9 in intestinal injury during SAP, we established MMP9 stable knockdown Caco2 and HT29 cells *in vitro* and generated a MMP9 knockout (MMP9−/−) mouse model of SAP *in vivo*.

**Results:** We found that the abundance of *Clostridium butyricum* (C. *butyricum*) was significantly decreased in the SAP group. In addition, overexpression of MMP9 notably downregulated the expressions of tight junction proteins and upregulated the expressions of p-p38 and p-ERK in Caco2 and HT29 cells (*p* < 0.05). However, C. *butyricum* or butyrate treatment remarkably upregulated the expressions of tight junction proteins and downregulated the expressions of MMP9, p-p38 and p-ERK in MMP9-overexpressed Caco2 and HT29 cells (*p* < 0.05). Importantly, C. *butyricum* or butyrate could not affect the expressions of tight junction proteins, and MMP9, p-p38 and p-ERK proteins in MMP9-knockdown cells compared with MMP9-knockdown group. Consistently, C. *butyricum* or butyrate could not attenuate pancreatic and intestinal injury during SAP in MMP9−/− mice compared with the SAP group.

**Conclusion:** Collectively, C. *butyricum* could protect against pancreatic and intestinal injury after SAP *via* downregulation of MMP9 *in vitro* and *in vivo*.

## Introduction

Acute pancreatitis (AP) is a common severe inflammatory disease of the pancreas characterized by immune infiltration, necrosis, abscess, hemorrhage, and pain (Leung, 2010; García-Alonso et al., 2012; Lankisch et al., 2015). According to the disease severity, AP is classified into three types: mild AP, moderately severe AP, and severe AP (SAP). SAP is the most serious subtype of AP with a high morbidity and mortality rate (Zerem, 2014). During the AP, excessive inflammatory cytokines were released into the bloodstream (Liang et al., 2021). These released cytokines induced the injury in intestinal barrier and intestinal microbiota dysbiosis and gut bacterial translocation (Liang et al., 2021). Once gut bacteria pass through the intestinal wall and enter the blood, it can affect multiple organs of the human body (Cen et al., 2018; Liang et al., 2021). A previous study showed that gut bacterial translocation is a key process for aggravating AP (Jia et al., 2020). Thus, gut microbiota may play an important role in the development of SAP.

Human gut microbiota plays an important role on the host’s health *via* interfering multiple intestinal functions (Nishijima et al., 2016; Meng et al., 2018). In addition, a healthy gut microbiota is characterized by large bacterial taxonomic richness and diversity, which has more than 1,500 species including Firmicutes, Bacteroidetes and Proteobacteria (Qin et al., 2010; Lozupone et al., 2012; Li X. Y. et al., 2020). Evidence has shown that gut microbiota in SAP is characterized by reduced bacterial diversity (Tan et al., 2015). Patients with SAP have a lower proportion of Bifidobacterium and a higher proportion of *Enterococcus* and Enterobacteriaceae in fecal microbiota (Tan et al., 2015). Thus, the use of gut microbiota therapies might be a potential approach for the treatment of SAP. In this study, 16s rRNA gene sequencing was used to analyze differences in the gut microbiota in fecal samples from patients with SAP and healthy controls, and we found that the abundance of Clostridiales was significantly decreased in the SAP group. Meanwhile, our previous study has found that *Clostridium butyricum* (C. *butyricum*) and its major metabolite butyrate could reduce intestinal injury in a rat model of SAP with intra-abdominal hypertension (Zhao et al., 2020). However, the mechanism by which C. *butyricum* regulates the intestinal barrier function in a mice model of SAP remains unclear.

Matrix metallopeptidase 9 (MMP9), a member of MMPs family, has been implicated in a variety of biological processes, such as inflammation and cell proliferation (Kou et al., 2020; Zhang et al., 2020; Ringland et al., 2021). Mechanically, MMP9 can degrade many extracellular matrix (ECM) proteins and remodel the dynamic balance of ECM (Huang, 2018). Evidence have shown that MMPs play an important role in the pathogenesis of gastrointestinal diseases by affecting matrix degradation, mucosal destruction, and inflammatory cell migration (Cervinková et al., 2014; Jiang et al., 2020). Pan et al. (2017) found that MMP9 expression was upregulated in intestinal tissues in a rat model of SAP. In addition, MMP9 overexpression could promote the loss of intestinal villous (Kocael et al., 2016). Conversely, intestinal tissues from MMP9−/− mice displayed reduced intestinal epithelial injury compared with wild type (WT) mice (Santana et al., 2006). These data indicated that MMP9 may play an important role in the development of intestinal injury. Thus, in this study, we aimed to investigate whether C. *butyricum* could exert protective roles on intestinal injury during SAP *via* modulating MMP9.

## Materials and Methods

### Human Subjects

Seventy-four patients who underwent SAP and admitted to Guangzhou First People’s Hospital and Changhai Hospital, from June 2018 to July 2020, were recruited in this study. In addition, thirty-two healthy controls were enrolled in this study. The inclusion criteria for healthy controls: participants had no prior history of illness and were in good mental health. The inclusion criteria for patients with SAP were based on the 2012 revised Atlanta criteria (Banks et al., 2013), and the exclusion criteria included chronic pancreatitis, cancer, diabetes, severe liver disease and inflammatory bowel disease. Approximately 1–2 g of fresh fecal samples from all participants were collected and then stored at −80°C. Written informed consent was obtained from all participants. This study has been approved by the Medical Ethics Committee of the South China University of Technology.

### 16S rRNA Gene Sequencing

Microbial genomic DNA was extracted from fecal samples with the HiPure Stool DNA Kits (Magen, Guangzhou, China). Next, the 16S rDNA target region of the ribosomal RNA gene was amplified by PCR and the cycling was as follows: 95°C for 5 min, followed by 30 cycles for 60 s at 95°C, 72°C for 60 s and 72°C for 7 min. After that, amplicons were purified using the AMPure XP Beads (Beckman Coulter, Inc., Union City, CA, Unites States). Subsequently, the purified amplicons were sequenced on an Illumina Novaseq 6,000 sequencing platform (Illumina Inc.) according to the protocol by Gene Denovo Biotechnology Co., Ltd.

The alpha diversity, including Chao1, ACE, Shannon and Simpson, was analyzed by Kruskal–Wallis test and Dunn’ test. In addition, Anosim test was used to evaluate the differences in microbiota abundance between SAP and control groups. Meanwhile, the indicator species analysis was conducted to screen the biomarker between SAP and control groups using R language labdsv package.

### Cell Culture and Transfection

The human colorectal adenocarcinoma cell lines (Caco-2 and HT-29) were purchased from Shanghai Zhongqiao Xinzhou Biotechnology Co., Ltd. and maintained in DMEM medium (BasalMedia, Shanghai, China) supplemented with 10% FBS (Thermo Fisher Scientific, Waltham, MA, United States) and incubated at 5% CO_2_ at 37°C.

The MMP9 sequence was synthesized by General Biol and then inserted into the pCDH-CMV-MCS-EF1-copGFP-T2A-Puro lentiviral expression vector plasmids (General Biol, Anhui, China), called MMP9 OE plasmids. In addition, lentivirus-containing shRNA targeting MMP9 (MMP9 shRNA1: 5′-GGA​ATA​CCT​GTA​CCG​CTA​TGG-3′, MMP9 shRNA2: 5′- GCA​GAC​ATC​GTC​ATC​CAG​TTT​C-3′ and MMP9 shRNA3: 5′-GCT​TAG​ATC​ATT​CCT​CAG​TGC-3′) were synthesized by GenePharma (Shanghai, China). After that, 293T cells were transfected with indicated lentiviral plasmids, packaging plasmids (pAX2) and envelope plasmid (pMD2. G) for 72 h. Next, virus-containing supernatants was filtered through a filter membrane (0.22 μm pore size), and then transduced into Caco-2 and HT-29 cells. Later on, the infected cells were selected by puromycin.

### Western Blot Assay

Cells were lysed in RIPA buffer (Beyotime, Beijing, China) and the concentration of the proteins were measured by a BCA protein assay kit (Sangon biotech, Shanghai, China). After that, proteins were subjected to SDS-PAGE gels and then transferred onto polyvinylidene fluoride (PVDF) membranes (Millipore, Billerica, MA, United States). The membranes were then incubated with primary antibodies targeting ZO-1 (1:1,000, Abcam Cambridge, MA, United States), MMP9 (1:1,000, Abcam, Claudin3 (1:1,000, Abcam), Claudin5 (1:1,000, Abcam), Occludin (1:1,000, Abcam), p-p38 (1:1,000, Cell Signaling Technology, Danvers, MA, United States), p38 (1:1,000, Cell Signaling Technology), p-ERK (1:1,000, Cell Signaling Technology), ERK (1:2,000, Cell Signaling Technology), α-tubulin (1:8,000, proteintech, Rosemont, IL, United States), and GAPDH (1:8000, proteintech) at 4°C overnight. Later on, the membranes were incubated with corresponding horseradish peroxidase (HRP)-conjugated secondary antibodies (Beyotime) at room temperature for 1 h. Subsequently, the blots were visualized by an ECL kit (Yeasen, Shanghai, China).

### Cell-Counting Kit-8 Assay

The Caco-2 and HT-29 cells were added into 96-well plates with 1 × 10^4^ cells per well and cultured overnight at 37°C. After that, cells were treated with different concentrations of butyrate (Sigma Aldrich, St. Louis, MO, United States) and incubated for 48 h. Later on, 10 µl of CCK-8 solution (Beyotime) added to each well and cells were then incubated for another 3 h. Next, the absorbance was detected with a microplate reader (Tecan M1000, Tecan, Switzerland) at 450 nm.

### Immunofluorescence Assay

Cells were fixed in 4% paraformaldehyde for 30 min at room temperature. After blocking with 1% BSA for 30 min, cells were incubated with the following primary antibodies: ZO-1 (1: 400, Abcam), Occludin (1: 400, Abcam), Claudin 3 (1: 400, Abcam), Claudin 5 (1: 400, Abcam) at 4°C overnight. Later on, cells were incubated with the Alexa Fluor 594 goat anti-rabbit IgG (H + L) secondary antibody (Yeasen) for 60 min at room temperature. Subsequently, images were captured with a fluorescence microscope. Nuclei were stained with DAPI for 5 min.

### Culture of C. *butyricum*

The C. *butyricum* was obtained from Shanghai qingdayingke Medical Technology Co., Ltd, and was cultured in Reinforced Clostridial Medium (Beijing Land Bridge Technology Co., Ltd, Beijing, China) at 37°C for 30 h in an anaerobic environment. After that, C. *butyricum* were washed 3 times with ice-cold PBS and resuspended with saline. The Caco-2 and HT-29 cells were co-cultured with C. *butyricum* for 6 h in an anaerobic environment.

### Animal Study

A total of 24 6-week-old C57BL/6-*Mmp9* ^*em1Smoc*^ mice (MMP9 gene knockout mice; males, SPF level) were obtained from Shanghai Model Organisms (Shanghai, China) and were then randomly assigned into four groups: control, SAP, SAP + C. *butyricum* and SAP + butyrate groups. All experimental protocols were performed according to the National Institutes of Health Laboratory Animal Guidelines and were approved by the Animal Ethics Committee of the South China University of Technology. Mice in the control or SAP group were orally gavaged with 0.9% saline (200 µl) for 1 week once a day; mice in the SAP + C. *butyricum* and SAP + butyrate groups were orally gavaged with C. *butyricum* (2 × 10^8^ CFU, 200 µl) or butyrate (200 mg/kg, 200 µl) respectively for 1 week once a day. After that, mice in the control group were injected intraperitoneally by 0.9% saline (200 µl) once an hour for 9 h. Mice in the SAP, SAP + C. *butyricum* and SAP + butyrate groups were intraperitoneally (i.p.) treated with caerulein (50 μg/kg) once an hour for 8 h. At 1 h after the final caerulein injection, mice were injected with LPS (10 mg/kg, i.p.) (Liu et al., 2017; Kong et al., 2021). After 1 h of LPS injection, all mice were sacrificed.

### Histological Analysis

The pancreatic and small intestine tissues were paraffin embedded and cut into 5 μM sections. After that, hematoxylin and eosin (H and E) were used to stain the sections. Subsequently, morphological changes were observed by using a light microscope.

### Immunohistochemistry Analysis

The paraffin-embedded sections were deparaffinized and rehydrated in graded alcohol series. Later on, the sections were boiled in pressure cooker for 3 min to retrieve antigen. After blocking with 1% BSA for 30 min at room temperature, the sections were incubated at 4°C overnight with the following primary antibodies: ZO-1 (1: 400, Abcam), Occludin (1: 400, Abcam), Claudin 3 (1: 400, Abcam), Claudin 5 (1: 400, Abcam). After that, the sections were incubated with the HRP-conjugated secondary antibody (Beyotime) for 50 min at 37°C. Subsequently, images were visualized with a light microscope.

### Transmission Electron Microscope Analysis

The small intestine tissues were fixed in 2.5% glutaraldehyde at 4°C overnight, and then fixed in 1% osmium tetroxide. After that, the samples were dehydrated in graded ethanol, embedded in epoxy resin, and then cut into ultra-thin sections (80 nm). Subsequently, the samples were observed in a HT7800 TEM (HITACHI, Japan).

### ELISA Assay

The levels of IL-1β (cat. no. ab197742; Abcam), IL-6 (cat. no. ab100712; Abcam), TNF-α (cat. no. ab208348; Abcam) and diamine oxidase (DAO; Fine Test, Wuhan, China) in serum samples were measured by ELISA kits. In addition, the levels of α-amylase (cat. no. H012; Beijing Leadman Biochemistry Co., Ltd., Beijing, China) and lipase (cat. no. H427; Beijing Leadman Biochemistry Co., Ltd., Beijing, China) in serum samples were detected by a HITACHI 7180 automatic biochemical analyzer (HITACHI).

### Statistical Analysis

Data are presented as the mean ± standard deviation (S.D.) Differences between three or more groups were analyzed by One-way analysis of variance (ANOVA) and Tukey’s tests. *p* values of <0.05 were considered statistically significant. All data were repeated independently at least three times.

## Results

### Difference in Species of Bacteria Between Control and Severe Acute Pancreatitis Groups

**FIGURE 1:**
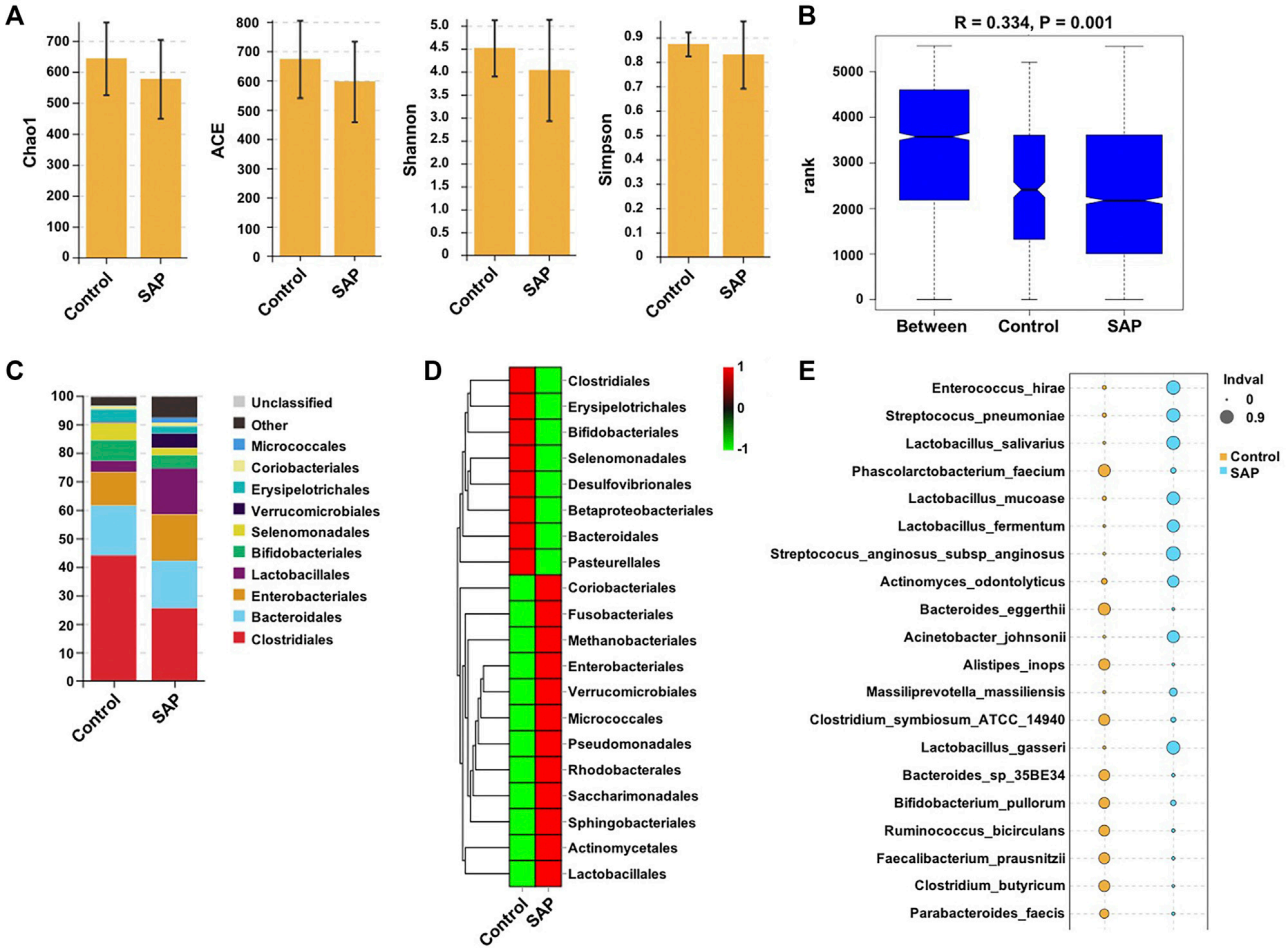
Difference in species of bacteria between control and SAP groups. 16s rRNA gene sequencing was used to determine the abundance and composition of gut microbiota in fecal samples from patients with SAP and controls. **(A)** Alpha diversity (Chao1, ACE, Shannon, Simpson) and **(B)** Beta diversity (Anosim) comparison between control and SAP groups. **(C)** The relative abundance of the microbiota at the order level was displayed using the stacked bar chart. **(D)** Heatmap showed the differences in microbiota abundance at genus level between SAP and control groups. **(E)** The indicator species analysis was conducted to screen the biomarker between SAP and control groups. SAP, severe acute pancreatitis.

To explore gut microbiome linked to the progression of SAP, the abundance and composition of gut microbiota in fecal samples between SAP and control groups were analyzed by 16s rRNA gene sequencing. First, Shannon and Simpson analyses were used to measure intestinal microbial diversity and ACE and Chao analyses were used to determine species richness. The data showed that microbial richness and diversity in the fecal samples was slightly, but not significantly, lower in the SAP group than that in the control group (*p* > 0.05, Figure 1A), indicating that the two groups had similar microbial alpha diversity. In addition, Anosim test (beta diversity) showed significant differences in bacterial community composition between SAP and control groups (Figure 1B). Collectively, there are significant changes in microbial communities between the two groups.

Next, we assessed whether there are changes in the relative abundance of the taxa. The results showed that the gut microbiota from individuals with SAP harbored a lower relative abundance of Clostridiales, Bacteroidales, Bifidobacteriales, Erysipelotrichales, Selenomonalales and a higher relative abundance of Enterobacteriales, Lactobacillales, Verrucomicrobiales, Coriobacteriales, Micrococcales compared with that from healthy controls (Figure 1C). In addition, the heatmap analysis showed that among the top 20 order with significant difference, the abundance of Clostridiales was notably decreased in the SAP group compared with the control group (Figure 1D).

The indicator species analysis was used to further compare the microbiota between SAP and control groups. As shown in Figure 1E and Supplementary Figure S1, the relative abundances of the species *Clostridium butyricum* (C. *butyricum*) were found to be lower in the fecal samples in patients with SAP compared with healthy controls.

### C. *butyricum* or Butyrate Treatment Reduced MMP9 Expression in Caco-2 and HT-29 Cells

**FIGURE 2:**
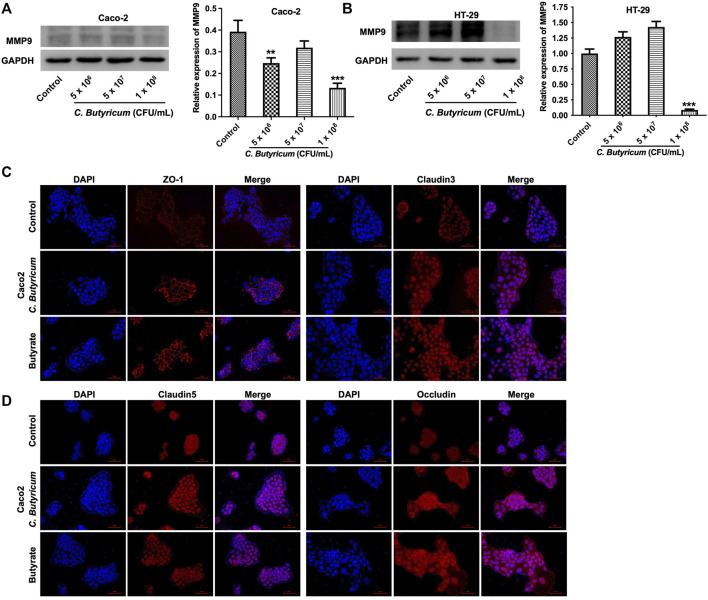
*Clostridium butyricum* or butyrate treatment reduced MMP9 expression in Caco-2 and HT-29 cells. **(A)** The Caco-2 and **(B)** HT-29 cells were co-cultured with **(C)** *butyricum* (5 × 10^6^, 5 × 10^7^, or 1 × 10^8^ CFU/ml) for 6 h in an anaerobic environment. After that, the supernatant was removed, and cells were then cultured in normoxia environment for 48 h. Western blot assay was used to determine the expression of MMP9 in cells. **(C,D)** Caco-2 cells were treated with 2 mM butyrate for 48 h. Meanwhile, cells were co-cultured with C. *butyricum* (1 × 10^8^ CFU/ml) for 6 h in an anaerobic environment. After that, the supernatant was removed, and cells were then cultured in normoxia environment for 48 h. IF assay was used to determine the expressions of ZO-1, Claudin3, Claudin5, Occludin in cells. **p < 0.01, ***p < 0.001 vs. control group. MMP9, matrix metallopeptidase 9; ZO-1, Zona occludens 1.

To investigate whether the protective role of C. *butyricum* in intestinal injury is associated with MMP9, we detected MMP9 expression in C. *butyricum*-treated Caco-2 and HT-29 cells. As indicated in Figures 2A,B *butyricum* (1 × 10^8^ CFU/mL) treatment remarkably downregulated the expression of MMP9 in Caco-2 and HT-29 cells. In addition, 2 and 1 mM butyrate had very limited effect on the viability of Caco-2 and HT-29 cells respectively (Supplementary Figures S2A,B). Thus, 2 and 1 mM of butyrate were used to treat Caco-2 and HT-29 cells in the following experiment respectively.

**FIGURE 3:**
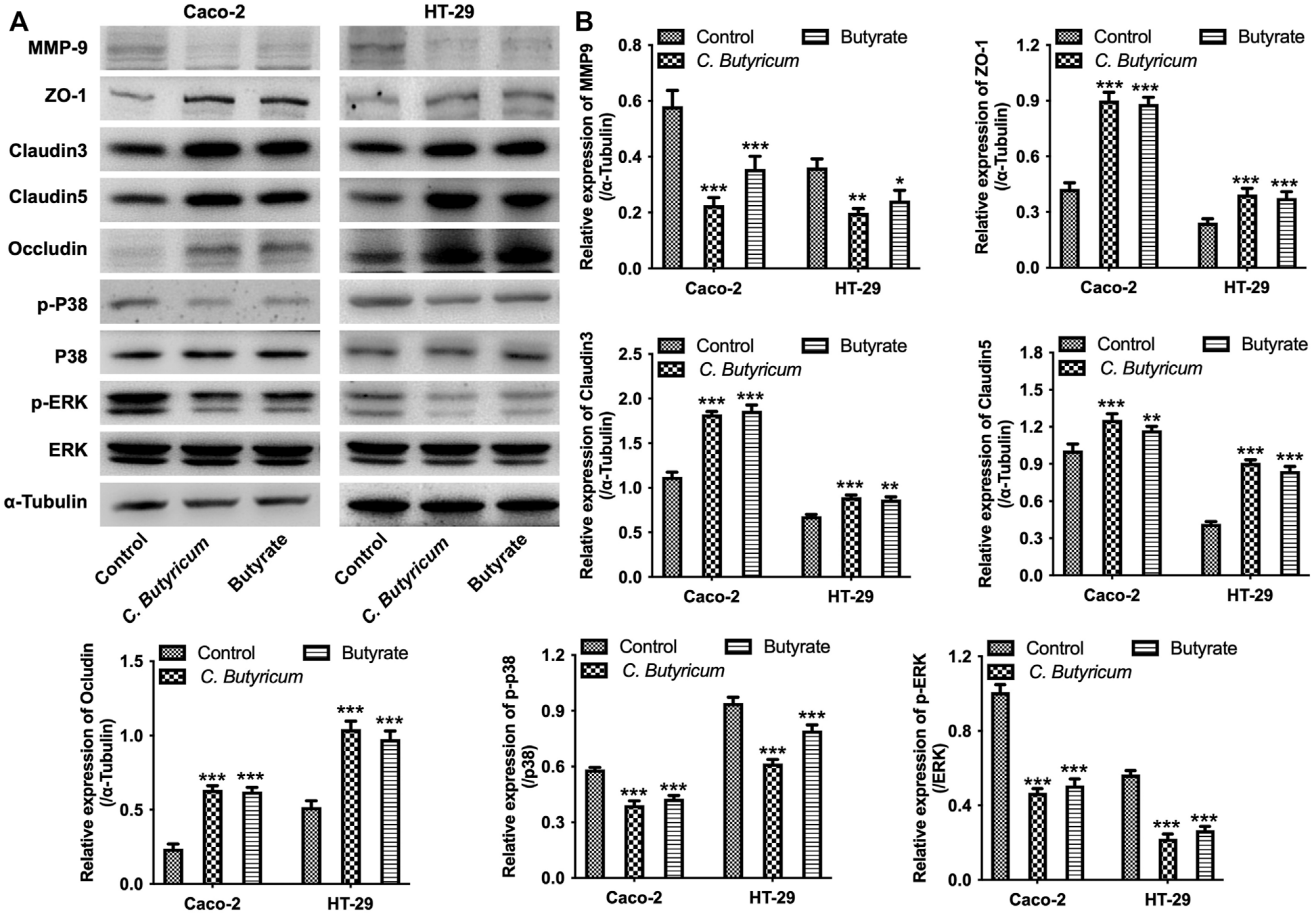
*Clostridium butyricum* or butyrate treatment reduced MMP9 expression and upregulated the expressions of tight junction proteins in Caco-2 and HT-29 cells. **(A,B)** Caco-2 and HT-29 cells were treated with butyrate (2 mM for Caco-2 cell and 1 mM for HT-29 cell) for 48 h. Meanwhile, Caco-2 and HT-29 cells were co-cultured with C. *butyricum* (1 × 10^8^ CFU/ml) for 6 h in an anaerobic environment. After that, the supernatant was removed, and cells were then cultured in normoxia environment for 48 h. Western blot assay was used to determine the expressions of MMP9, ZO-1, Claudin3, Claudin5, Occludin, p-p38, p-ERK in cells. *p < 0.05, **p < 0.01, ***p < 0.001 vs. control group. MMP9, matrix metallopeptidase 9; ZO-1, Zona occludens 1.

Furthermore, IF and western blot assays results showed that C. *butyricum* or butyrate treatment notably increased the expressions of tight junction proteins ZO-1, Claudin3, Claudin5, Occludin in Caco-2 and HT-29 cells compared with the control group (Figures 2C,D, 3A,B and Supplementary Figures S3A,B). Meanwhile, C. *butyricum* or butyrate treatment remarkably reduced the expressions of MMP9, p-p38 and p-ERK in Caco-2 and HT-29 cells compared to the control group (Figures 3A,B). To sum up, C. *butyricum* or butyrate treatment could reduce MMP9 expression and upregulate the expressions of tight junction proteins in Caco-2 and HT-29 cells.

### C. *butyricum* or Butyrate Treatment Protected MMP9-Induced the Destruction of Intercellular Tight Junction in Caco-2 and HT-29 Cells

**FIGURE 4:**
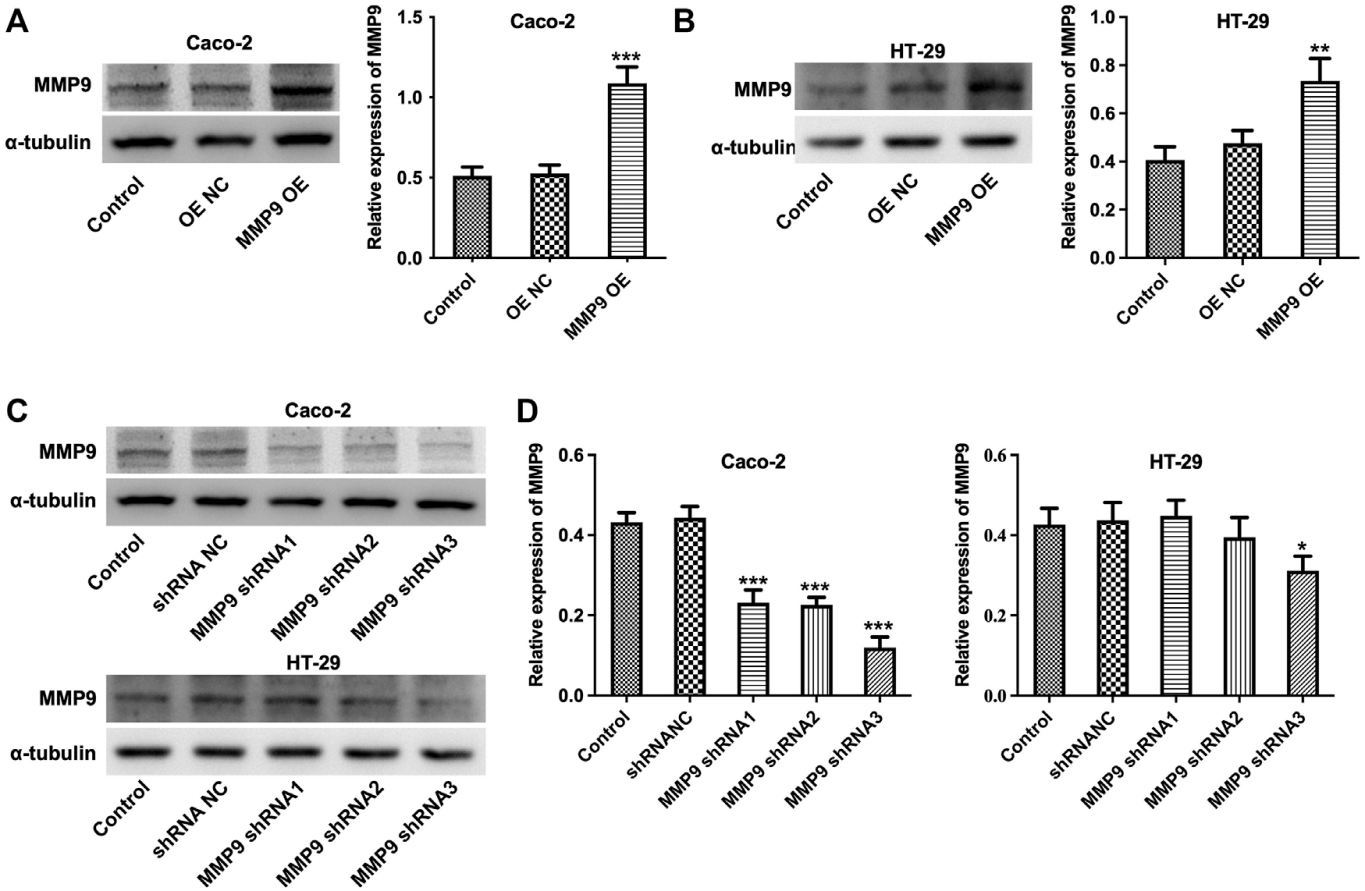
Establishment of MMP9 overexpressing and MMP9 knockdown Caco-2 and HT-29 cells. **(A,B)** Caco-2 and HT-29 cells were transfected with MMP9 OE plasmids. Western blot assay was used to determine the expression of MMP9 in cells. **p < 0.01, ***p < 0.001 vs. OE NC group. **(C,D)** Caco-2 and HT-29 cells were transfected with MMP9 shRNA1, MMP9 shRNA2 or MMP9 shRNA3 plasmids. Western blot assay was used to determine the expression of MMP9 in cells. *p < 0.05, ***p < 0.001 vs. shRNA NC group. MMP9, matrix metallopeptidase 9; OE NC, overexpression negative control; shRNA NC, short hairpin RNA negative control.

To further investigate whether MMP9 participate in C. *butyricum*-mediated protective effects of intestinal injury, we overexpressed the expression of MMP9 in Caco-2 and HT-29 cells (Figures 4A,B). Meanwhile, three different shRNAs (MMP9 shRNA1, MMP9 shRNA2, and MMP9 shRNA3) was used to reduce the expression of MMP9 in Caco-2 and HT-29 cells (Figures 4C,D). Since MMP9 shRNA3 exhibited the best inhibitory effect on MMP9 expression comparing with MMP9 shRNA1 or MMP9 shRNA2, MMP9 shRNA3 was used in the next experiments.

**FIGURE 5:**
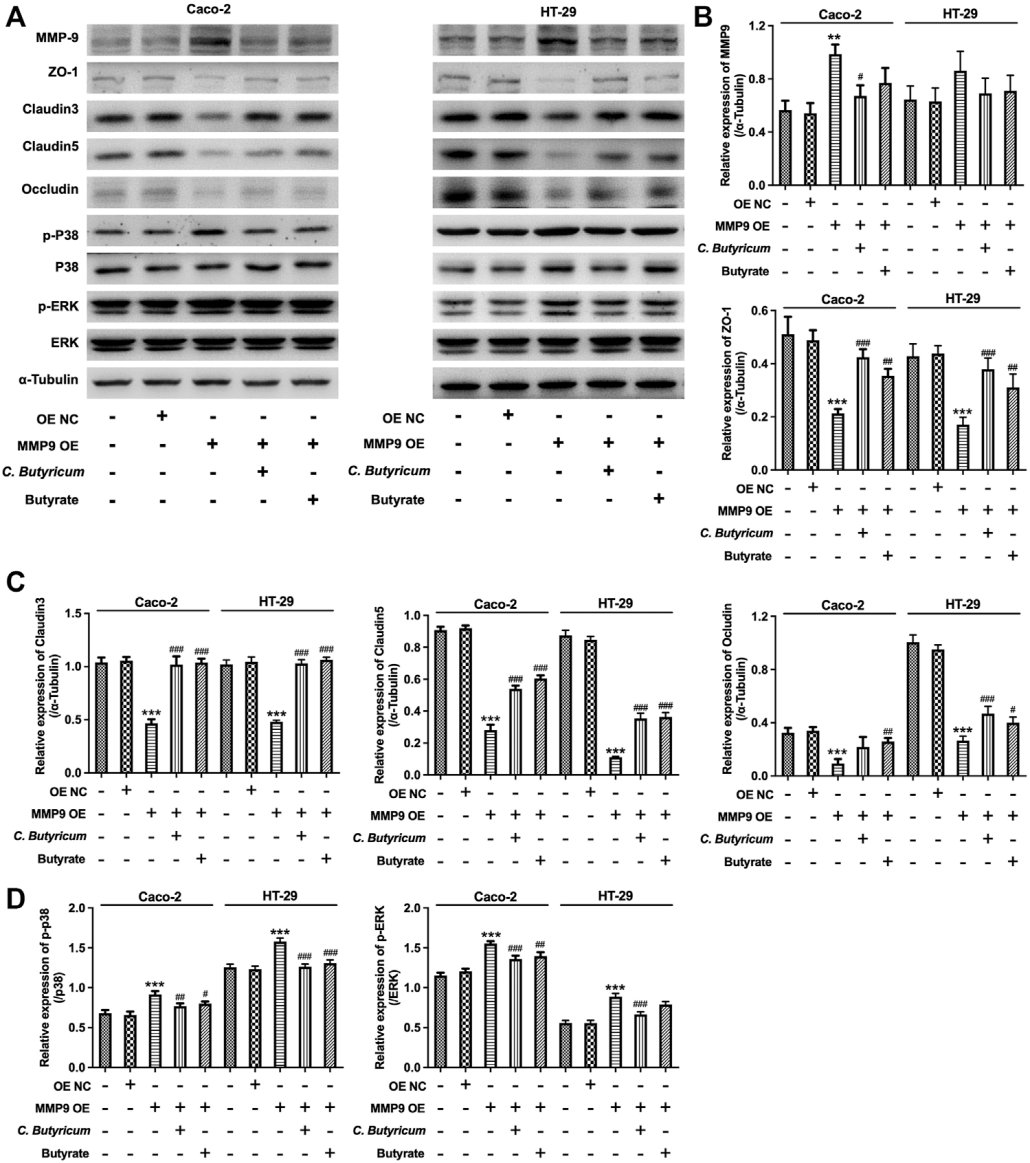
*Clostridium butyricum* or butyrate treatment protected MMP9-induced the destruction of intercellular tight junction in Caco-2 and HT-29 cells. **(A–D)** Caco-2 and HT-29 cells were transfected with MMP9 OE plasmids. The transfected Caco-2 and HT-29 cells were then treated with butyrate (2 mM for Caco-2 cell and 1 mM for HT-29 cell) for 48 h. Meanwhile, the transfected Caco-2 and HT-29 cells were co-cultured with C. *butyricum* (1 × 10^8^ CFU/ml) for 6 h in an anaerobic environment. After that, the supernatant was removed, and cells were then cultured in normoxia environment for 48 h. Western blot assay was used to determine the expressions of MMP9, ZO-1, Claudin3, Claudin5, p-p38, p-ERK in cells. **p < 0.01, ***p < 0.001 vs. control group; ^#^ *p* < 0.05, ^##^ *p* < 0.01, ^###^ *p* < 0.001 vs. MMP9 OE group. MMP9, matrix metallopeptidase 9; OE NC, overexpression negative control; ZO-1, Zona occluden 1.

**FIGURE 6:**
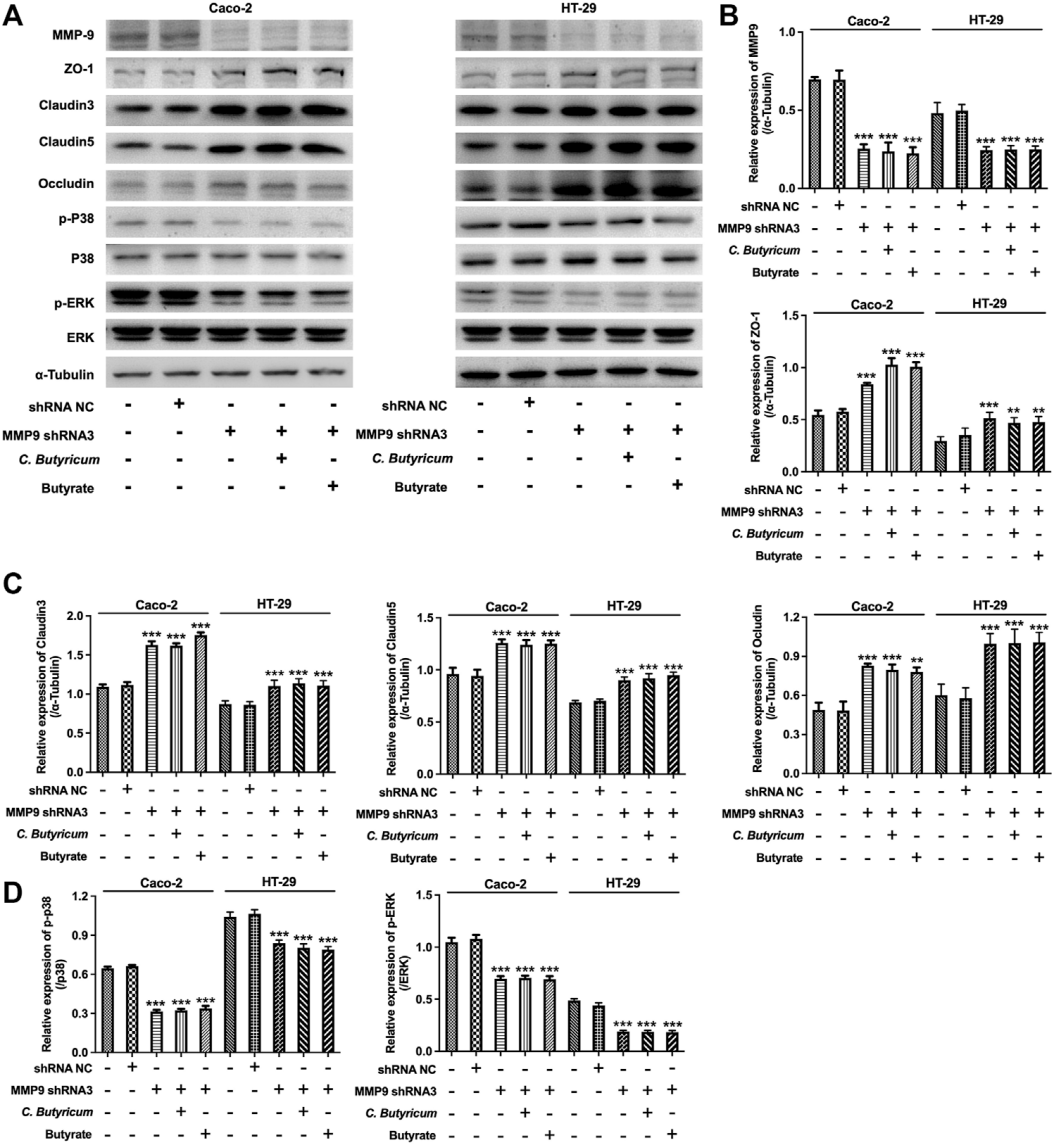
*Clostridium butyricum* or butyrate could not exert protective roles on intercellular tight junction in MMP9-knockdown Caco-2 and HT-29 cells. **(A–D)** Caco-2 and HT-29 cells were transfected with MMP9 shRNA3 plasmids. The transfected Caco-2 and HT-29 cells were then treated with butyrate (2 mM for Caco-2 cell and 1 mM for HT-29 cell) for 48 h. Meanwhile, the transfected Caco-2 and HT-29 cells were co-cultured with C. *butyricum* (1 × 10^8^ CFU/ml) for 6 h in an anaerobic environment. After that, the supernatant was removed, and cells were then cultured in normoxia environment for 48 h. Western blot assay was used to determine the expressions of MMP9, ZO-1, Claudin3, Claudin5, p-p38, p-ERK in cells. **p < 0.01, ***p < 0.001 vs. control group. MMP9, matrix metallopeptidase 9; shRNA NC, short hairpin RNA negative control; ZO-1, Zona occludens 1.

Furthermore, the results of western blot assay showed that upregulation of MMP9 markedly elevated the expressions of MMP9, p-p38 and p-ERK, and downregulated the expressions of ZO-1, Claudin3, Claudin5 and Occludin in Caco-2 and HT-29 cells; however, these changes were notably reversed by C. *butyricum* or butyrate treatment (Figures 5A–D). In addition, downregulation of MMP9 significantly decreased the expressions of MMP9, p-p38 and p-ERK, and increased the expressions of ZO-1, Claudin3, Claudin5, Occludin in Caco-2 and HT-29 cells. (Figures 6A–D). However, C. *butyricum* or butyrate treatment caused no differences in the expressions of MMP9, ZO-1, Claudin3, Claudin5, Occludin, p-p38 and p-ERK in MMP9 knockdown Caco2 and HT29 cells compared with MMP9 shRNA3 group (Figures 6A–D). Collectively, C. *butyricum* or butyrate treatment protected MMP9-induced the destruction of intercellular tight junction in Caco2 and HT29 cells.

### C. *butyricum* or Butyrate Could Not Exert Protective Roles on Intestinal Injury During Severe Acute Pancreatitis in MMP9−/− Mice

**FIGURE 7:**
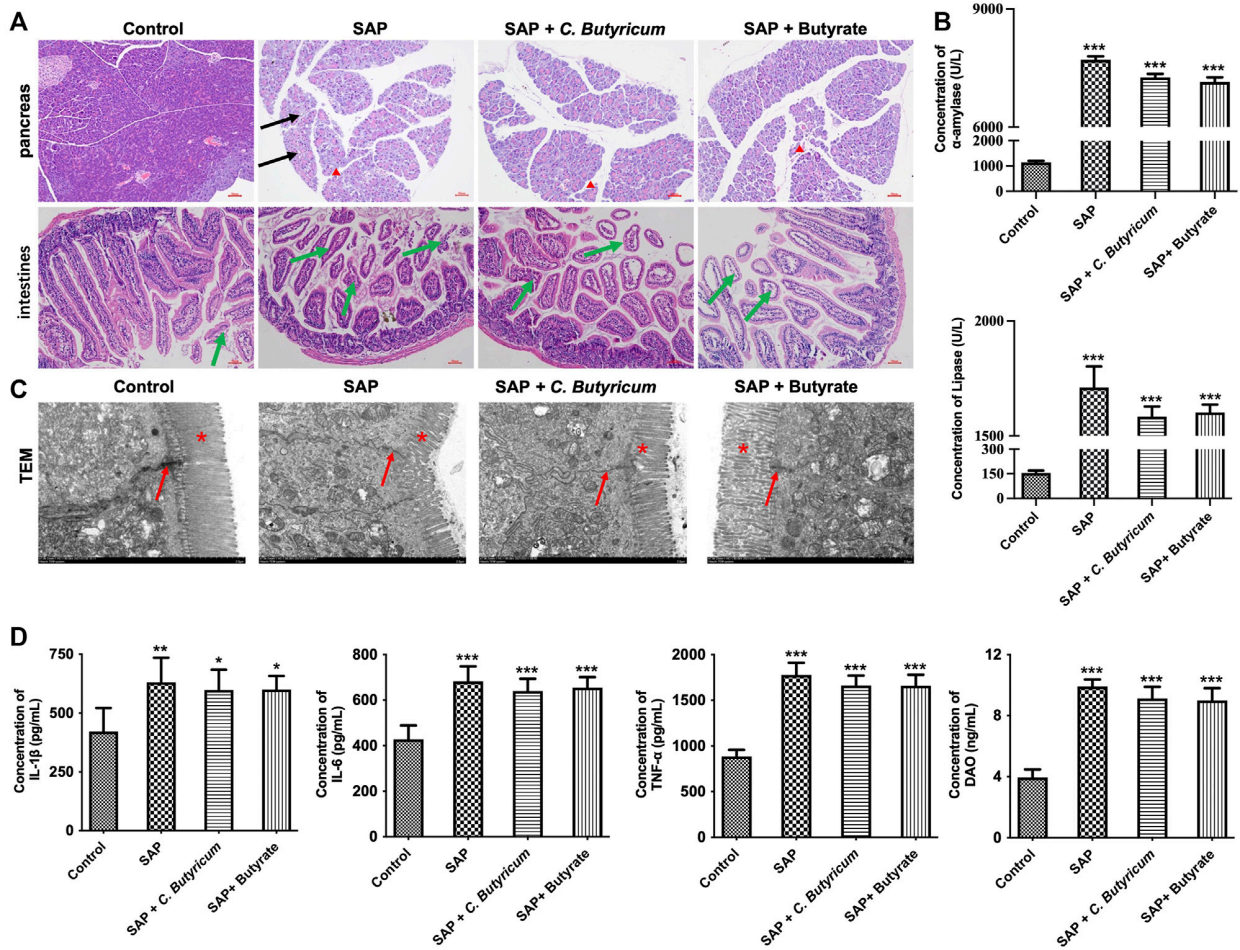
*Clostridium butyricum* or butyrate could not exert protective roles on intestinal injury during SAP in MMP9−/− mice. **(A)** H and E staining showed the pathological changes in the pancreas and small intestine tissues. The black arrows denote acinar cells and the red triangle denote interstitial edema in pancreas tissues. The green arrows denote crypt atrophy in small intestine tissues. **(B)** ELISA assay was used to detect the levels of *α*-amylase and lipase in serum samples of MMP9−/− mice. **(C)** The intestinal epithelial villi in MMP9−/− mice was observed by TEM. The red arrow represents tight junctions; the red star represents intestinal microvilli. **(D)** The levels of IL-1β, IL-6, TNF-α and DAO in serum samples of MMP9−/− mice was detected by ELISA. *p < 0.05, **p < 0.01, ***p < 0.001 vs. control group. diamine oxidase, DAO; MMP9, matrix metallopeptidase 9; SAP, severe acute pancreatitis; TEM, transmission electron microscope.

To further investigate whether C. *butyricum* or butyrate could exert protective roles on intestinal inflammation and barrier integrity during SAP *via* modulating MMP9, MMP9−/− mice were subjected to caerulein and LPS. The results of HE staining assay showed that the pancreas tissues of MMP9−/− mice from SAP group displayed increased intracellular vacuoles in acinar cells, interstitial edema and acinar cell necrosis, hemorrhage, compared with the control group; however, C*. butyricum* or butyrate treatment did not restore the damaged pancreas tissues compared with the SAP group (Figure 7A). In addition, the levels of α-amylase and lipase in serum samples were significantly increased in the SAP group compared with the control group (Figure 7B). However, C. *butyricum* or butyrate treatment had no effects on the levels of α-amylase and lipase in serum samples compared with the SAP group (Figure 7B). Collectively, C. *butyricum* or butyrate could not attenuate pancreas injury during SAP in MMP9−/− mice.

**FIGURE 8:**
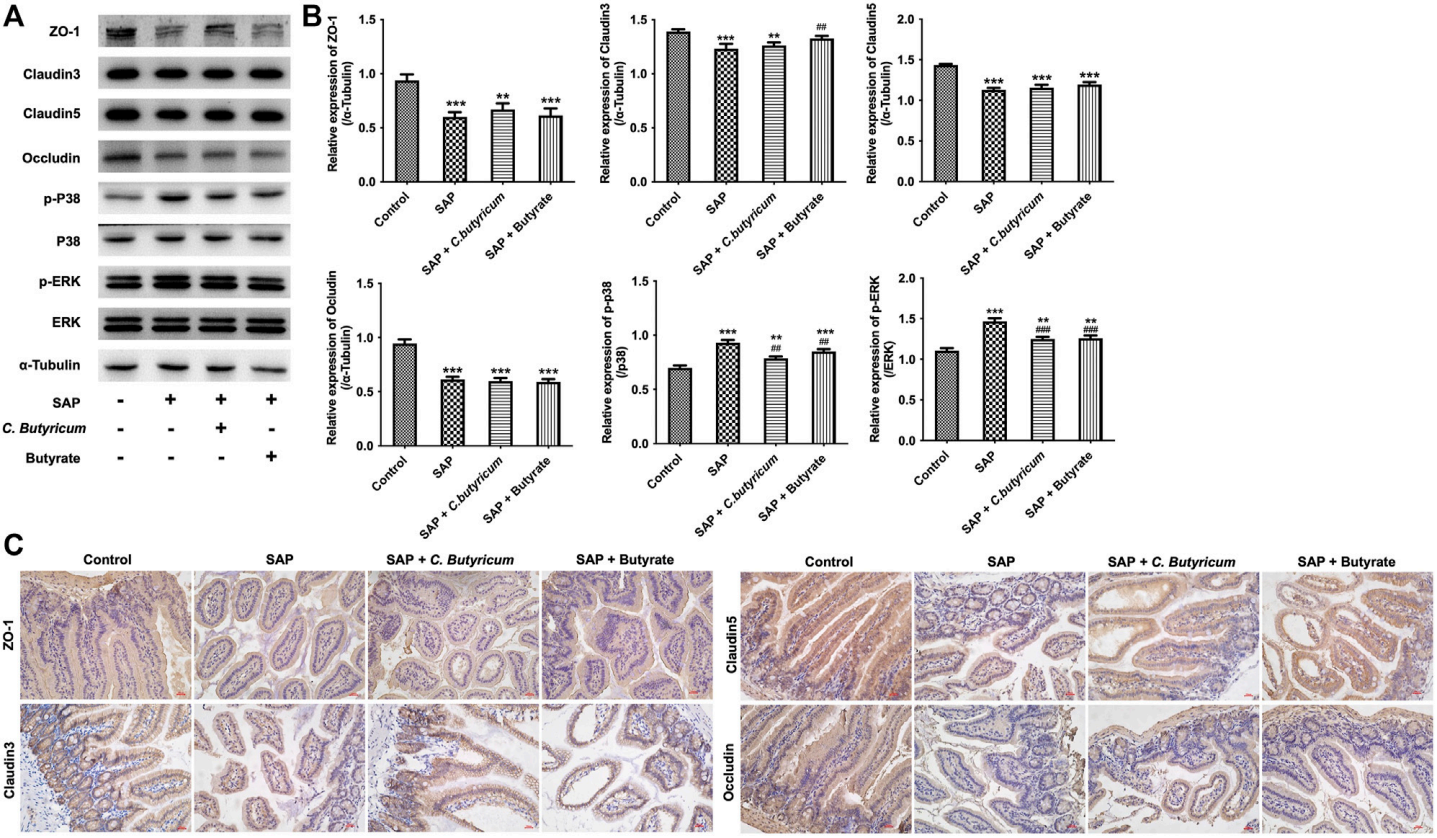
*Clostridium butyricum* or butyrate could not exert protective roles on intestinal injury during SAP in MMP9−/− mice *via* regulation of p38 and ERK MAPK signaling pathways. **(A,B)** Western blot analysis of the expressions of ZO-1, Claudin3, Claudin5, Occludin, p-p38, p-ERK in small intestine tissues of MMP9−/− mice. **(C)** IHC analysis of the expressions of ZO-1, Claudin3, Claudin5, Occludin in small intestine tissues of MMP9−/− mice. **p < 0.01, ***p < 0.001 vs. control group; ^##^ *p* < 0.01, ^###^ *p* < 0.001 vs. SAP group. MMP9, matrix metallopeptidase 9; ZO-1, Zona occludens 1.

Furthermore, MMP9−/− mice subjected to caerulein and LPS displayed increased intestinal injury, as determined by reduction of goblet cells, crypt atrophy, villi necrosis and edema (Figure 7A). However, C. *butyricum* or butyrate treatment caused no major histopathological changes in the intestine tissues of MMP9−/− mice compared with the SAP group (Figure 7A). Moreover, TEM observation showed dysfunctional cell junctions and villous shedding and disordered arrangement in intestines tissues of MMP9−/− mice from SAP group compared with the control group; however, C. *butyricum* or butyrate treatment did not change these effects in MMP9−/− mice (Figure 7C). Meanwhile, the levels of IL-1β, IL-6, TNF-α and DAO were notably increased in serum samples in MMP9−/− mice from SAP group; however, C. *butyricum* or butyrate treatment failed to reduce their levels in MMP9−/− mice compared with the SAP group (Figure 7D). Significantly, the expressions of ZO-1, Claudin3, Claudin5, Occludin were notably downregulated in intestines tissues from MMP9−/− mice during SAP, compared with the control group; however, C. *butyricum* or butyrate treatment did not change their expressions in MMP9−/− mice (Figures 8A–C). Additionally, the expressions of p-p38 and p-ERK were markedly upregulated in intestines tissues from MMP9−/− mice during SAP, whereas these changes were reversed by C*. butyricum* or butyrate treatment (Figures 8A,B). Collectively, C. *butyricum* or butyrate could not attenuate intestinal injury during SAP in MMP9−/− mice.

## Discussion

Evidence have shown that SAP is an acute inflammatory disease of the pancreas that is associated with intestinal barrier dysfunction (Tu et al., 2014; Zhang et al., 2015). In addition, the intestinal epithelial barrier is strongly interacted with the richness and diversity of gut microbial community (Takiishi et al., 2017). Gut microbiota dysbiosis could affect the intestinal barrier function, which in turn affect the development of SAP (Takiishi et al., 2017; Wan et al., 2019; Li J. et al., 2020). Zhu et al. (2019) found that patients with SAP have a lower proportion of beneficial bacteria such as *Blautia* in fecal samples compared with patients with mild AP and moderately SAP. Yu et al. (2020) reported that *Enterococcus* was significantly higher and *Eubacterium hallii* was remarkedly lower in abundance in rectal swab samples from patients with SAP. Thus, maintaining the balance of gut microbiota might be a potential approach for the treatment of SAP (Tang et al., 2019). In this study, we studied the gut microbial community between patients with SAP and healthy controls by sequencing 16S rRNA gene of the microbiota. Our data showed that the abundance of C. *butyricum* was significantly decreased in the SAP group. In addition, C. *butyricum* or its major metabolite butyrate treatment significantly upregulated the expressions of tight junction proteins (ZO-1, claudin-3, claudin-5, occludin) in Caco-2 and HT-29 cells, suggesting that C. *butyricum* or butyrate could enhance the intercellular tight junction. Moreover, our previous study showed that C*. butyricum* or butyrate treatment could attenuate intestinal injury caused by SAP in rats *via* maintaining the intestinal barrier function (Zhao et al., 2020). Consistent with our results, Pan et al. (2019) reported that C*. butyricum* treatment markedly attenuated intestinal inflammation and barrier dysfunction in mice during SAP. However, the underlying mechanism by which *C. butyricum* or butyrate protects intestinal barrier function remains unclear.

It has been shown that MMPs could increase endothelial cell permeability *via* disruption of tight junction proteins (Apostolidou et al., 2012). In addition, Yang et al. (2016) found that inhibition of MMP9 could attenuate IL-1β-induced tight junction proteins disruption. In addition, MMP9 has been found to play an important role in the development of the intestinal injury (Lucafò et al., 2019; Wu et al., 2019). Overexpression of MMP9 could facilitate the loss of intestinal villous (Kocael et al., 2016). In the present study, our results showed that overexpression of MMP9 significantly downregulated the expressions of tight junction proteins in Caco-2 and HT-29 cells, whereas MMP9 knockdown exhibited the opposite effects, suggesting that MMP9 could induce the disruption of the intercellular tight junction *in vitro*. Thus, we further investigated whether C. *butyricum* or butyrate could exert protective roles on intestinal injury during SAP *via* downregulating MMP9 expression. Our results showed that *C. butyricum* or butyrate significantly upregulated the expressions of tight junction proteins in MMP9-overexpressed Caco-2 and HT-29 cells, suggesting that C. *butyricum* or butyrate treatment could protect MMP9-induced the destruction of intercellular tight junction in Caco-2 and HT-29 cells. However, C. *butyricum* or butyrate could not affect intercellular tight junction in MMP9 knockdown Caco-2 and HT-29 cells. Consistently, C. *butyricum* or butyrate could not improve intestinal injury during SAP in MMP9−/− mice. However, in our previous study, a rat model with SAP was established and injuries of intestinal tissues were evaluated in C. *butyricum*-treated mice, and we found that C*. butyricum* or butyrate could attenuate intestinal injury caused by SAP in wild type rats (Zhao et al., 2020). These data indicated that the protective effect of C. *butyricum* or butyrate on intestinal injury during SAP was absent in MMP9−/− mice, demonstrating the importance of MMP9 for C*. butyricum* or butyrate-mediated protective effects on intestinal injury. Collectively, C. *butyricum* or butyrate could exert protective roles on intestinal injury during SAP *via* downregulation of MMP9.

ERK and p38 signaling pathways can mediate intestinal epithelial barrier permeability through affecting intestinal epithelial tight junctions and cell injury (Tang et al., 2021; Zhang et al., 2021). Mao et al. (2020) found that probiotic *Lactobacillus rhamnosus* could improve intestinal barrier function in LPS-treated piglets *via* inhibition of ERK and p38 signalings. In addition, Al-Sadi et al. (2019) reported that MMP9 could increase the intestinal epithelial tight permeability *via* activation of p38 MAPK signaling pathway. In this study, we found that overexpression of MMP9 significantly increased the expressions of p-p38 and p-ERK in Caco-2 and HT-29 cells; however, these changes were reversed by C. *butyricum* or butyrate treatment. These data showed that C. *butyricum* or butyrate could attenuate intestinal injury during SAP by downregulating MMP9 through p38 and ERK signaling pathways.

A limitation of the current study is that we used the commercial product of C. *butyricum* not the isolated C. *butyricum* strain to investigate whether C. *butyricum* could exert protective roles on intestinal injury during SAP *via* modulating MMP9. In the future, we aimed to determine the strain of the isolated C. *butyricum* and investigate the effect of the isolated C. *butyricum* on intestinal barrier functions.

## Conclusion

In this study, we found that C. *butyricum* or butyrate treatment could protect against pancreatic and intestinal Injury after SAP *via* downregulation of MMP9 *in vitro* and *in vivo*. Thus, C. *butyricum* or butyrate might be a possible therapeutic drug to diminish intestinal injury during SAP.

## Data Availability

The raw data supporting the conclusion of this article will be made available by the authors, without undue reservation.
