# Corrigendum: *Clostridium butyricum* protects against pancreatic and intestinal injury after severe acute pancreatitis via downregulation of MMP9

**DOI:** 10.3389/fphar.2023.1197442

**Published:** 2023-05-09

**Authors:** Qingqing Yan, Lin Jia, Biyan Wen, Yao Wu, Yanbo Zeng, Qing Wang

**Affiliations:** ^1^ Department of Gastroenterology, Guangzhou First People’s Hospital, South China University of Technology, Guangzhou, China; ^2^ Department of Gastroenterology, The Second Affiliated Hospital, School of Medicine, South China University of Technology, Guangzhou, China; ^3^ Department of Gastroenterology, The First Affiliated Hospital of Nan Chang University, Nanchang, China; ^4^ Department of Gastroenterology, Changhai Hospital, Shanghai, China

**Keywords:** severe acute pancreatitis, *Clostridium butyricum*, gut microbiota, matrix metallopeptidase 9, intestinal, injury

In the published article, there was an error in the **Funding statement**. “The funding statement for the Guangzhou Technology Projects was displayed as “No.202102020099”. The original text is that This study was supported by grants from the National Natural Science Foundation of China (No. 81870438) and the Guangzhou Technology Projects (No. 202102020099).” The correct **Funding statement** appears below.

“Funding

This study was supported by grants from The National Natural Science Foundation of China (No.81870438) and the Guangzhou Technology Projects (No.202102080099).”

The authors apologize for this error and state that this does not change the scientific conclusions of the article in any way. The original article has been updated.

