# Supplementary figures and images for: Clostridium butyricum Protects Against Pancreatic and Intestinal Injury After Severe Acute Pancreatitis via Downregulation of MMP9 (part 2 of 2)
Source: Front Pharmacol. 2022 Jul 18;13:919010. doi: 10.3389/fphar.2022.919010 (PMC9342915; doi:10.3389/fphar.2022.919010)

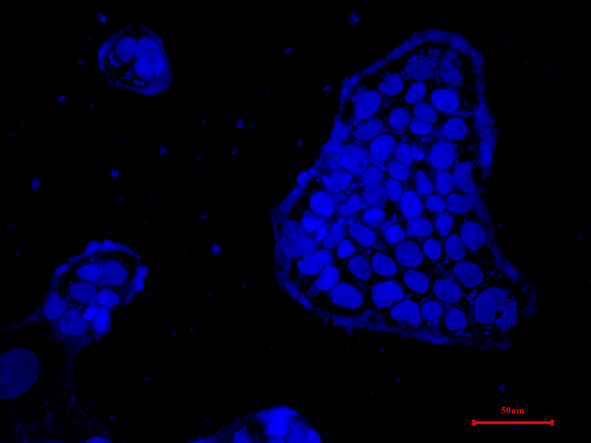

Supplement: Supplementary file 1 [file DataSheet1.ZIP › Data sources/Figure 2C, 2D, S3-IF/Figure 2C,D-Caco2/3-Claudin5/CON+C.buty/DAPI-4.jpg]

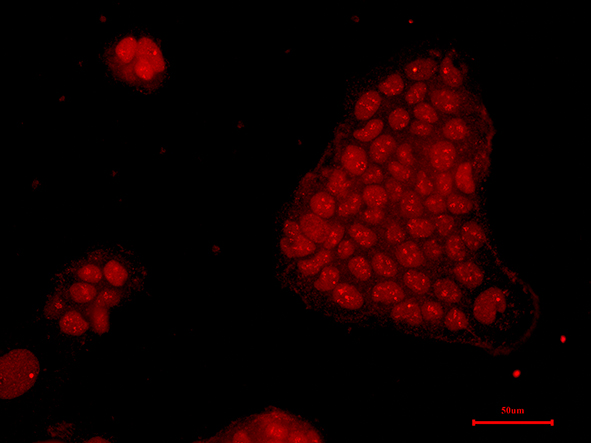

Supplement: Supplementary file 1 [file DataSheet1.ZIP › Data sources/Figure 2C, 2D, S3-IF/Figure 2C,D-Caco2/3-Claudin5/CON+C.buty/c5-4.jpg]

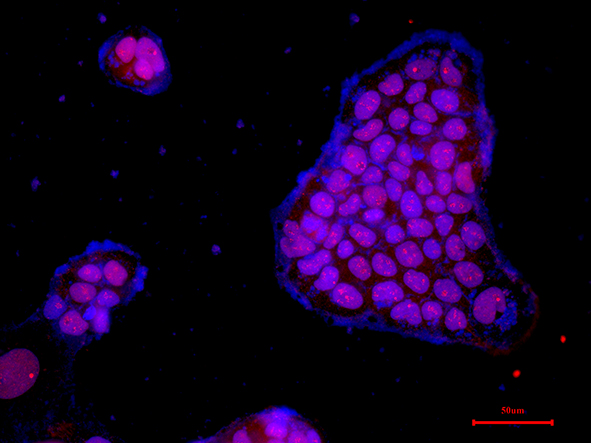

Supplement: Supplementary file 1 [file DataSheet1.ZIP › Data sources/Figure 2C, 2D, S3-IF/Figure 2C,D-Caco2/3-Claudin5/CON+C.buty/12.jpg]

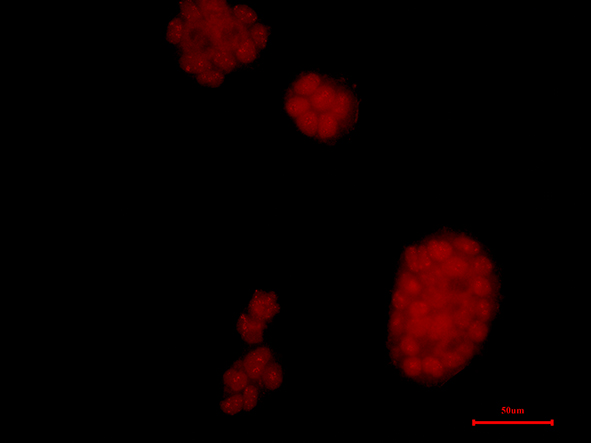

Supplement: Supplementary file 1 [file DataSheet1.ZIP › Data sources/Figure 2C, 2D, S3-IF/Figure 2C,D-Caco2/3-Claudin5/C0N/con-c5-4.jpg]

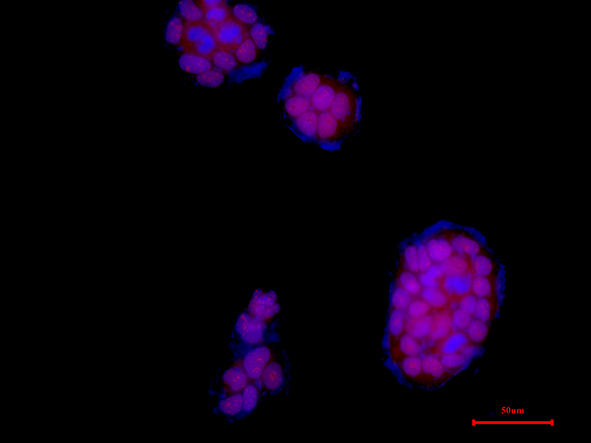

Supplement: Supplementary file 1 [file DataSheet1.ZIP › Data sources/Figure 2C, 2D, S3-IF/Figure 2C,D-Caco2/3-Claudin5/C0N/merge.jpg]

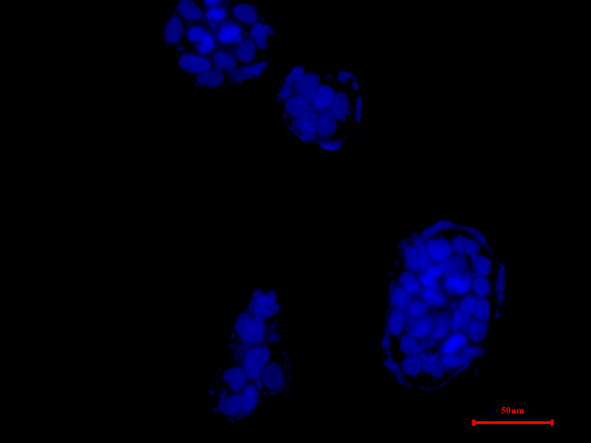

Supplement: Supplementary file 1 [file DataSheet1.ZIP › Data sources/Figure 2C, 2D, S3-IF/Figure 2C,D-Caco2/3-Claudin5/C0N/con-DAPI-4.jpg]

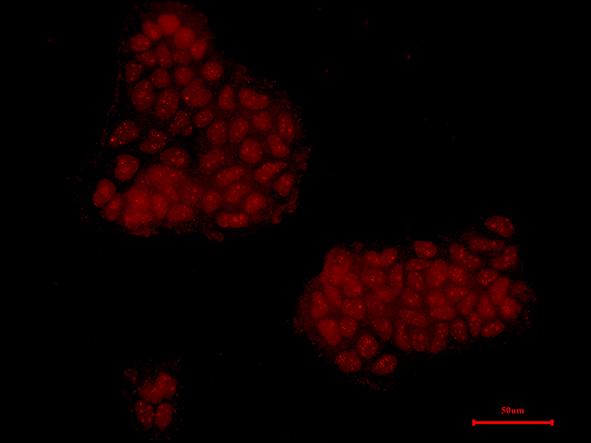

Supplement: Supplementary file 1 [file DataSheet1.ZIP › Data sources/Figure 2C, 2D, S3-IF/Figure 2C,D-Caco2/3-Claudin5/CON+buty/c5-2.jpg]

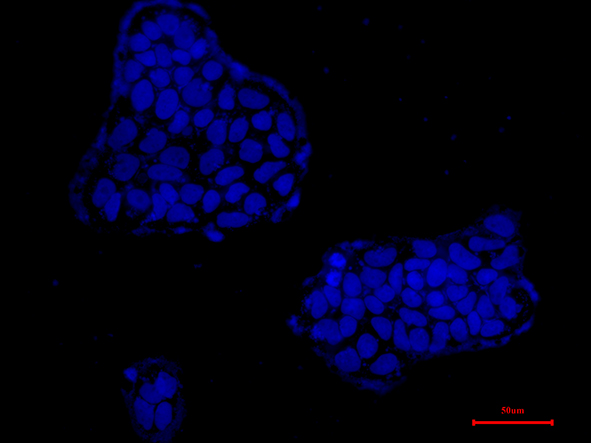

Supplement: Supplementary file 1 [file DataSheet1.ZIP › Data sources/Figure 2C, 2D, S3-IF/Figure 2C,D-Caco2/3-Claudin5/CON+buty/DAPI-2.jpg]

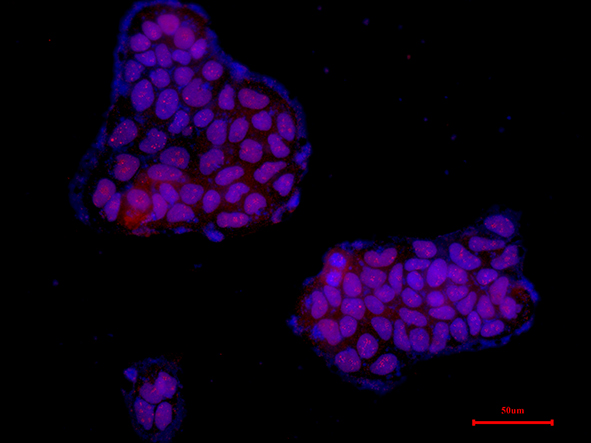

Supplement: Supplementary file 1 [file DataSheet1.ZIP › Data sources/Figure 2C, 2D, S3-IF/Figure 2C,D-Caco2/3-Claudin5/CON+buty/6.jpg]

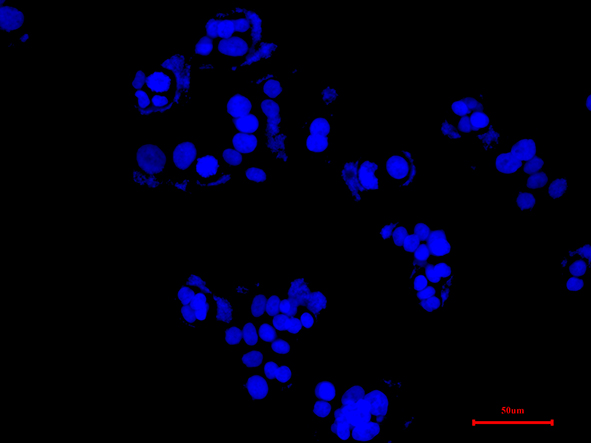

Supplement: Supplementary file 1 [file DataSheet1.ZIP › Data sources/Figure 2C, 2D, S3-IF/Figure S3-HT29/1-ZO-1/CON+C.buty/DAPI-2.jpg]

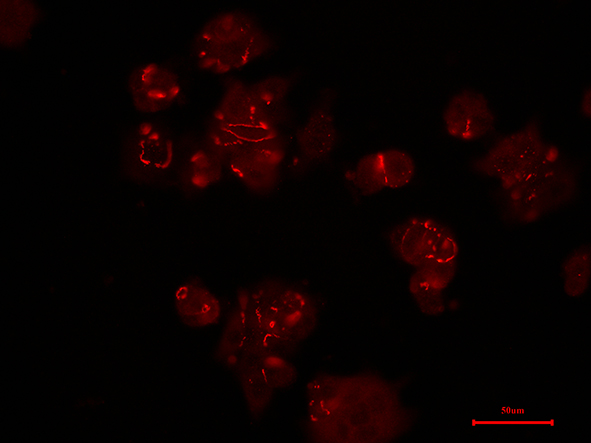

Supplement: Supplementary file 1 [file DataSheet1.ZIP › Data sources/Figure 2C, 2D, S3-IF/Figure S3-HT29/1-ZO-1/CON+C.buty/ZO1-2.jpg]

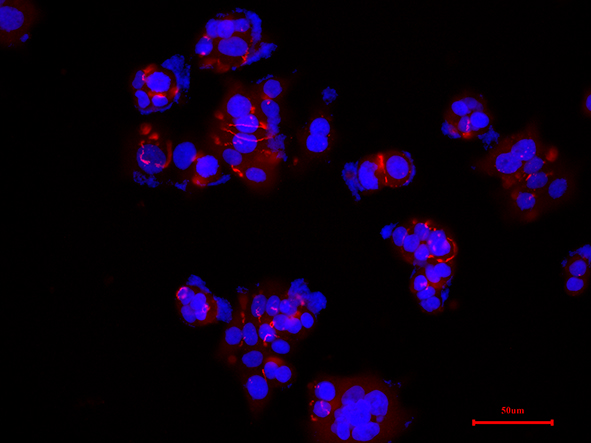

Supplement: Supplementary file 1 [file DataSheet1.ZIP › Data sources/Figure 2C, 2D, S3-IF/Figure S3-HT29/1-ZO-1/CON+C.buty/merge.jpg]

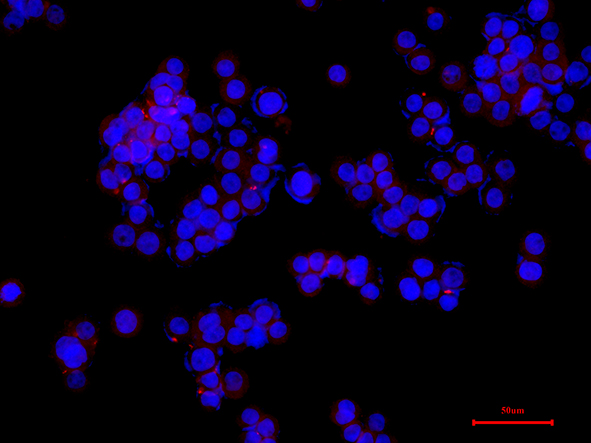

Supplement: Supplementary file 1 [file DataSheet1.ZIP › Data sources/Figure 2C, 2D, S3-IF/Figure S3-HT29/1-ZO-1/C0N/merge.jpg]

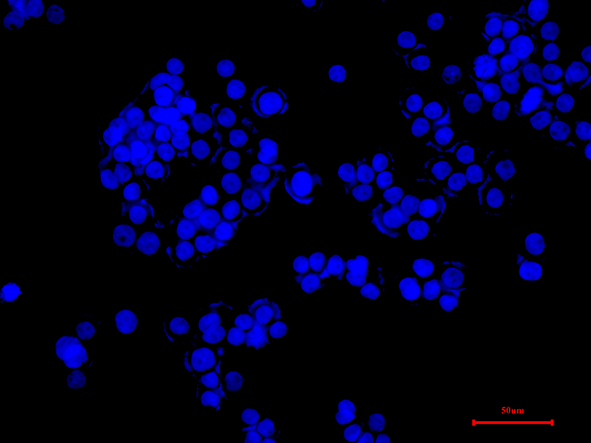

Supplement: Supplementary file 1 [file DataSheet1.ZIP › Data sources/Figure 2C, 2D, S3-IF/Figure S3-HT29/1-ZO-1/C0N/con-DAPI-2.jpg]

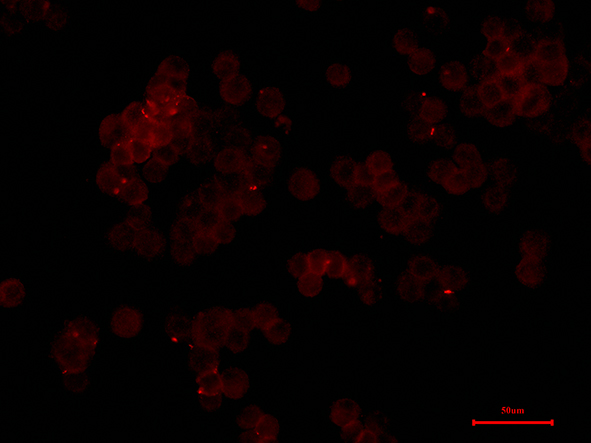

Supplement: Supplementary file 1 [file DataSheet1.ZIP › Data sources/Figure 2C, 2D, S3-IF/Figure S3-HT29/1-ZO-1/C0N/con-ZO1-2.jpg]

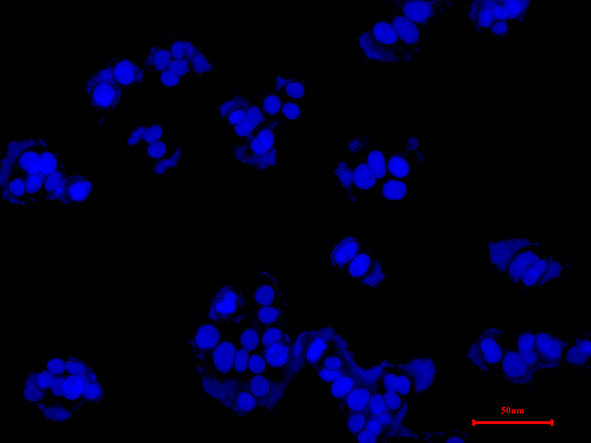

Supplement: Supplementary file 1 [file DataSheet1.ZIP › Data sources/Figure 2C, 2D, S3-IF/Figure S3-HT29/1-ZO-1/CON+buty/DAPI-3.jpg]

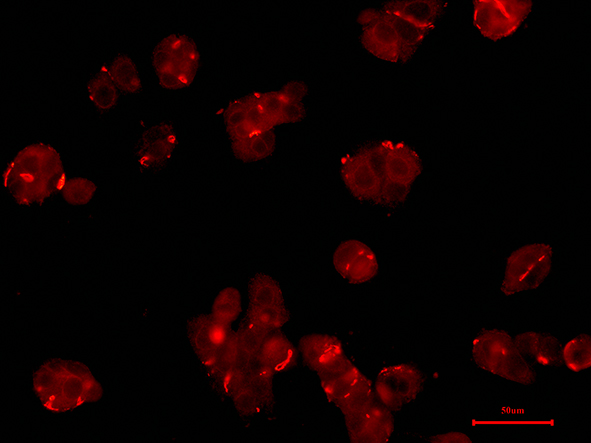

Supplement: Supplementary file 1 [file DataSheet1.ZIP › Data sources/Figure 2C, 2D, S3-IF/Figure S3-HT29/1-ZO-1/CON+buty/ZO1-3.jpg]

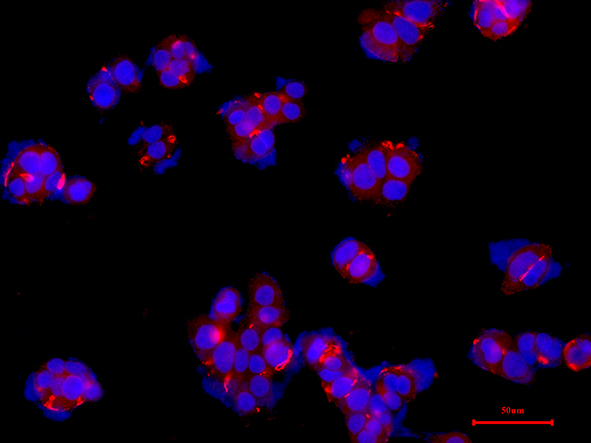

Supplement: Supplementary file 1 [file DataSheet1.ZIP › Data sources/Figure 2C, 2D, S3-IF/Figure S3-HT29/1-ZO-1/CON+buty/merge.jpg]

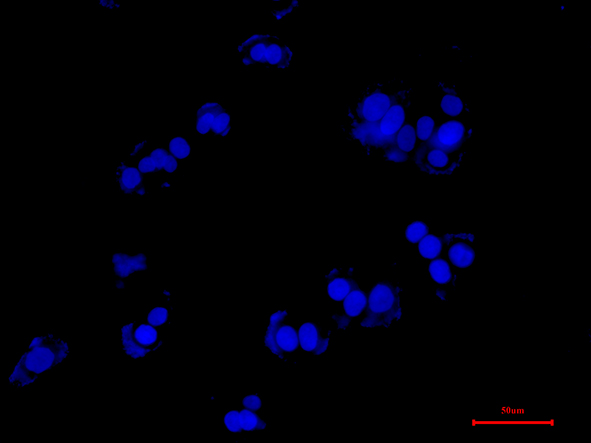

Supplement: Supplementary file 1 [file DataSheet1.ZIP › Data sources/Figure 2C, 2D, S3-IF/Figure S3-HT29/4-Occludin/CON+C.buty/DAPI-4.jpg]

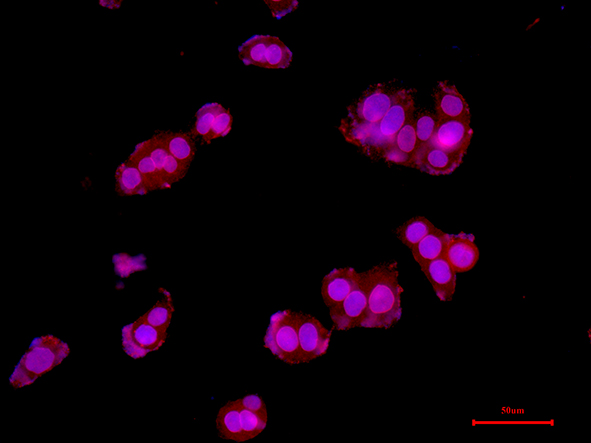

Supplement: Supplementary file 1 [file DataSheet1.ZIP › Data sources/Figure 2C, 2D, S3-IF/Figure S3-HT29/4-Occludin/CON+C.buty/merge.jpg]

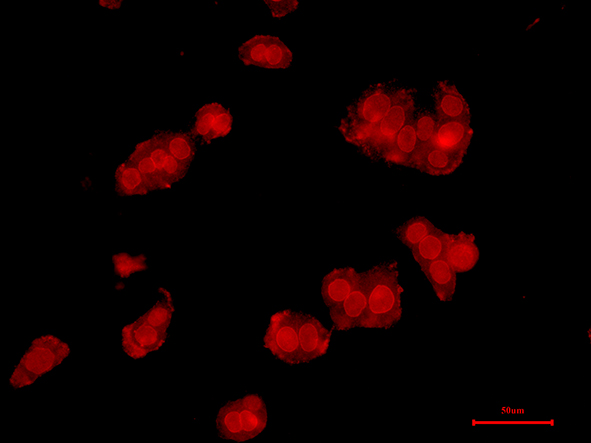

Supplement: Supplementary file 1 [file DataSheet1.ZIP › Data sources/Figure 2C, 2D, S3-IF/Figure S3-HT29/4-Occludin/CON+C.buty/occ-3.jpg]

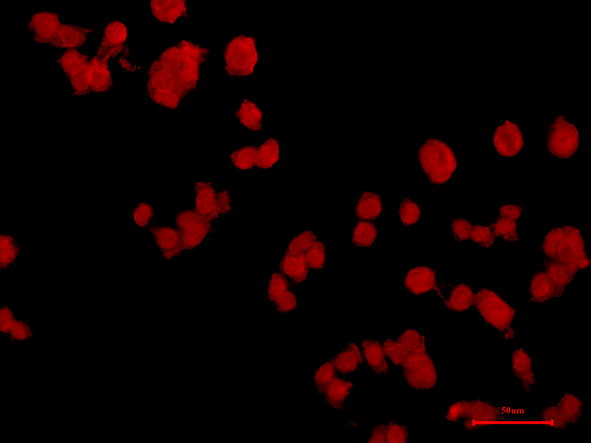

Supplement: Supplementary file 1 [file DataSheet1.ZIP › Data sources/Figure 2C, 2D, S3-IF/Figure S3-HT29/4-Occludin/C0N/con-occ-4.jpg]

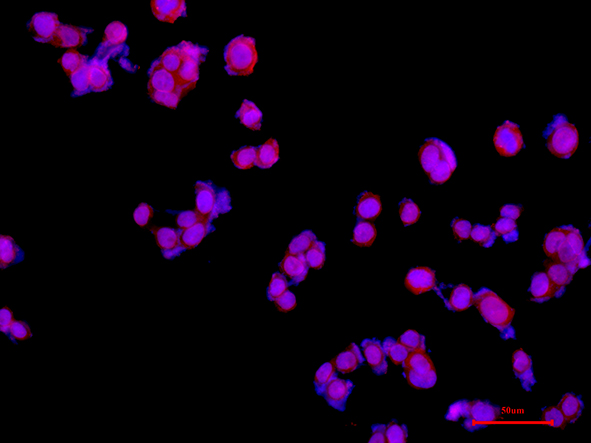

Supplement: Supplementary file 1 [file DataSheet1.ZIP › Data sources/Figure 2C, 2D, S3-IF/Figure S3-HT29/4-Occludin/C0N/merge.jpg]

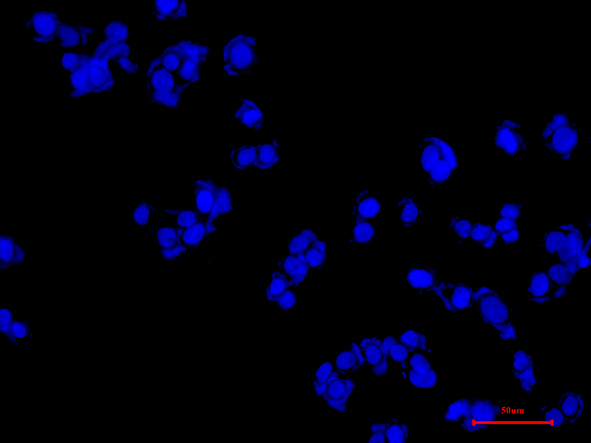

Supplement: Supplementary file 1 [file DataSheet1.ZIP › Data sources/Figure 2C, 2D, S3-IF/Figure S3-HT29/4-Occludin/C0N/con-DAPI-4.jpg]

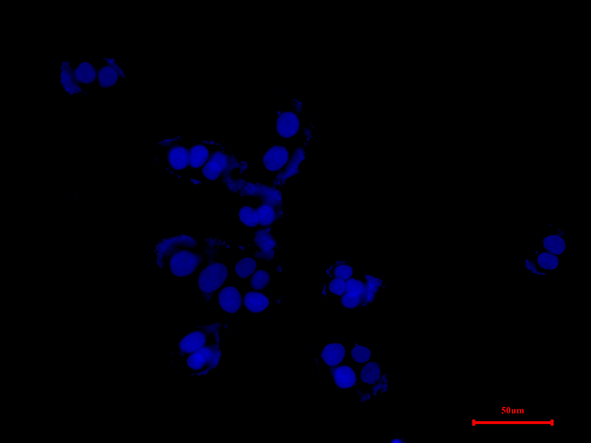

Supplement: Supplementary file 1 [file DataSheet1.ZIP › Data sources/Figure 2C, 2D, S3-IF/Figure S3-HT29/4-Occludin/CON+buty/DAPI-3.jpg]

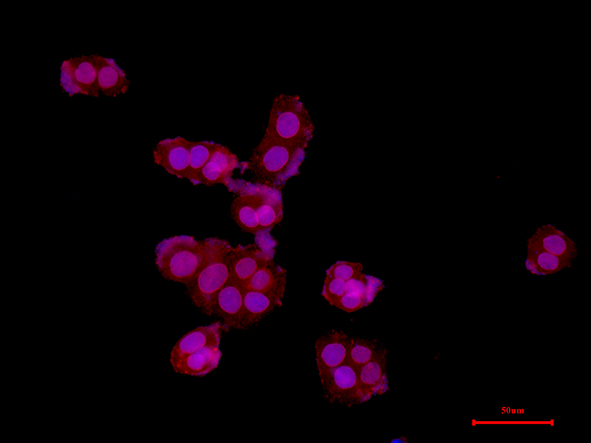

Supplement: Supplementary file 1 [file DataSheet1.ZIP › Data sources/Figure 2C, 2D, S3-IF/Figure S3-HT29/4-Occludin/CON+buty/merge.jpg]

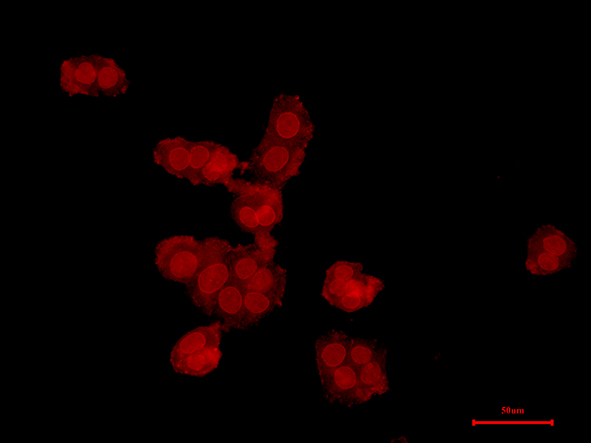

Supplement: Supplementary file 1 [file DataSheet1.ZIP › Data sources/Figure 2C, 2D, S3-IF/Figure S3-HT29/4-Occludin/CON+buty/occ-3.jpg]

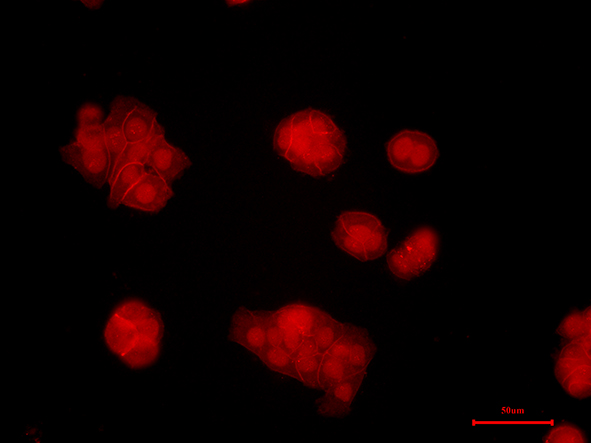

Supplement: Supplementary file 1 [file DataSheet1.ZIP › Data sources/Figure 2C, 2D, S3-IF/Figure S3-HT29/2-Claudin3/CON+C.buty/C3-1.jpg]

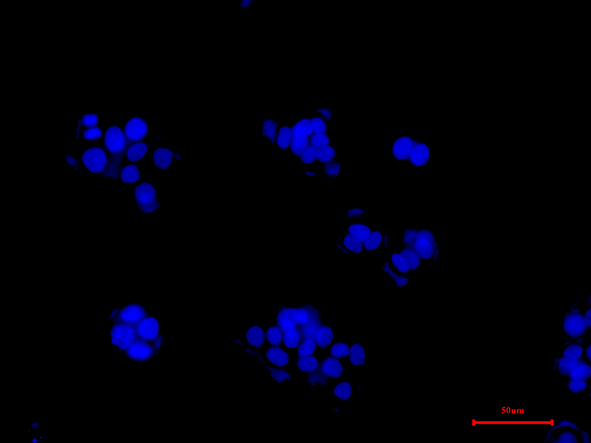

Supplement: Supplementary file 1 [file DataSheet1.ZIP › Data sources/Figure 2C, 2D, S3-IF/Figure S3-HT29/2-Claudin3/CON+C.buty/DAPI-1.jpg]

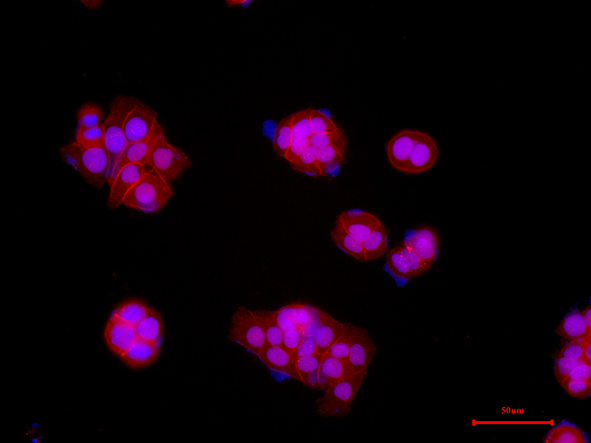

Supplement: Supplementary file 1 [file DataSheet1.ZIP › Data sources/Figure 2C, 2D, S3-IF/Figure S3-HT29/2-Claudin3/CON+C.buty/merge.jpg]

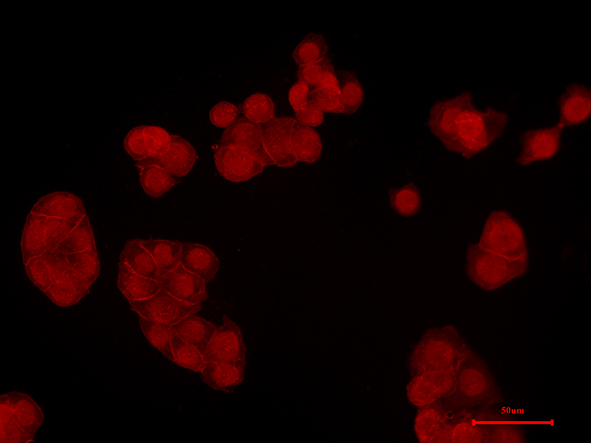

Supplement: Supplementary file 1 [file DataSheet1.ZIP › Data sources/Figure 2C, 2D, S3-IF/Figure S3-HT29/2-Claudin3/C0N/con-C3-3.jpg]

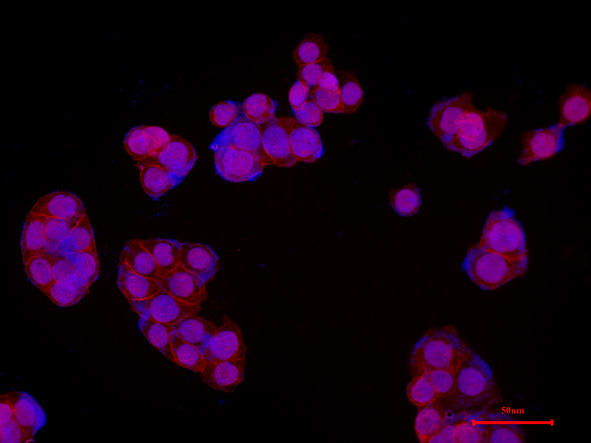

Supplement: Supplementary file 1 [file DataSheet1.ZIP › Data sources/Figure 2C, 2D, S3-IF/Figure S3-HT29/2-Claudin3/C0N/merge.jpg]

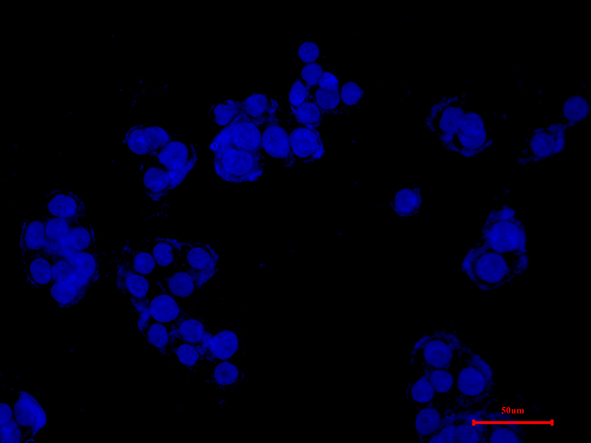

Supplement: Supplementary file 1 [file DataSheet1.ZIP › Data sources/Figure 2C, 2D, S3-IF/Figure S3-HT29/2-Claudin3/C0N/con-DAPI-3.jpg]

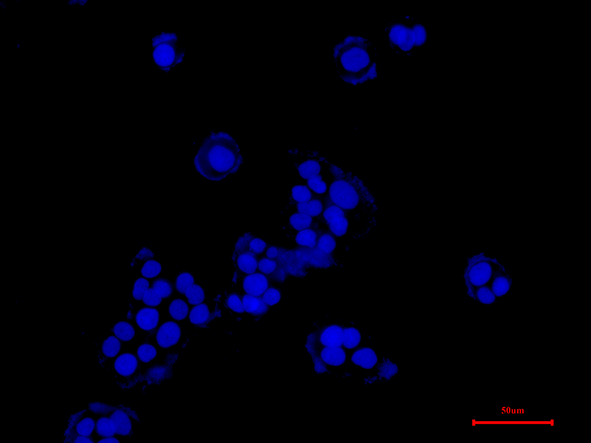

Supplement: Supplementary file 1 [file DataSheet1.ZIP › Data sources/Figure 2C, 2D, S3-IF/Figure S3-HT29/2-Claudin3/CON+buty/DAPI-2.jpg]

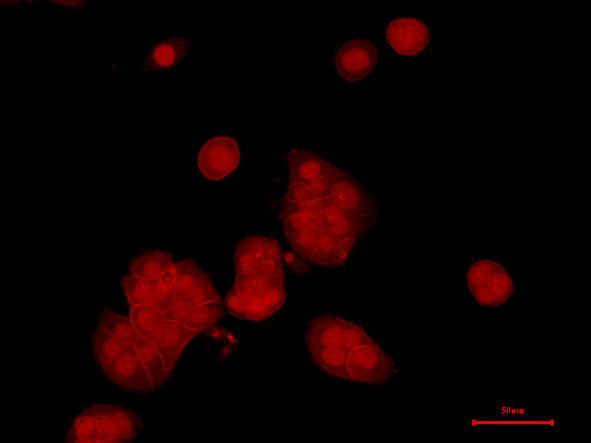

Supplement: Supplementary file 1 [file DataSheet1.ZIP › Data sources/Figure 2C, 2D, S3-IF/Figure S3-HT29/2-Claudin3/CON+buty/C3-2.jpg]

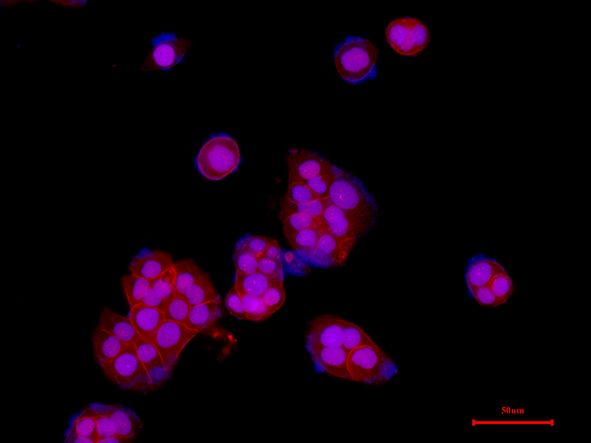

Supplement: Supplementary file 1 [file DataSheet1.ZIP › Data sources/Figure 2C, 2D, S3-IF/Figure S3-HT29/2-Claudin3/CON+buty/merge.jpg]

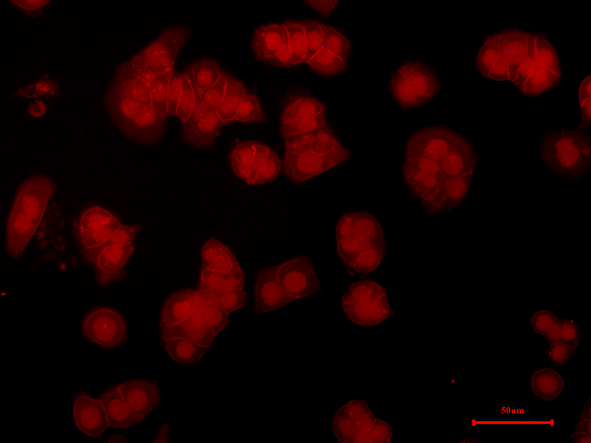

Supplement: Supplementary file 1 [file DataSheet1.ZIP › Data sources/Figure 2C, 2D, S3-IF/Figure S3-HT29/3-Claudin5/CON+C.buty/C5-2.jpg]

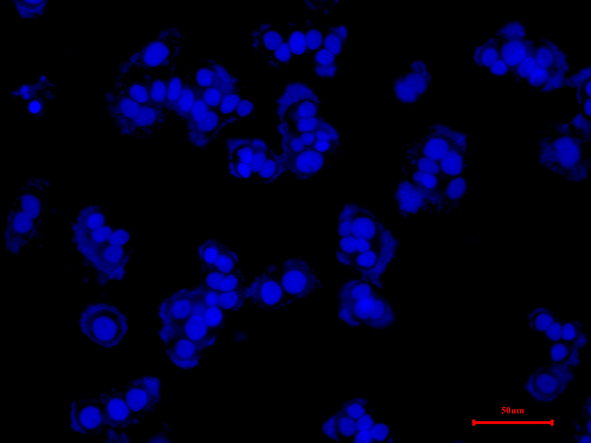

Supplement: Supplementary file 1 [file DataSheet1.ZIP › Data sources/Figure 2C, 2D, S3-IF/Figure S3-HT29/3-Claudin5/CON+C.buty/DAPI-2.jpg]

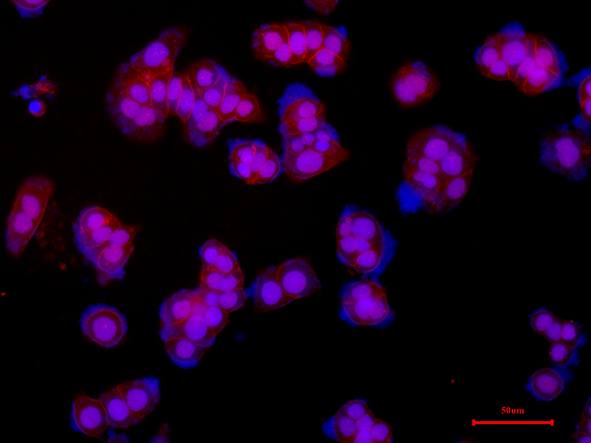

Supplement: Supplementary file 1 [file DataSheet1.ZIP › Data sources/Figure 2C, 2D, S3-IF/Figure S3-HT29/3-Claudin5/CON+C.buty/merge.jpg]

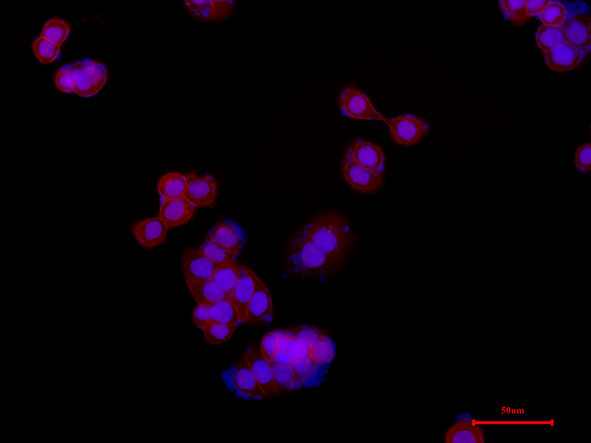

Supplement: Supplementary file 1 [file DataSheet1.ZIP › Data sources/Figure 2C, 2D, S3-IF/Figure S3-HT29/3-Claudin5/C0N/merge.jpg]

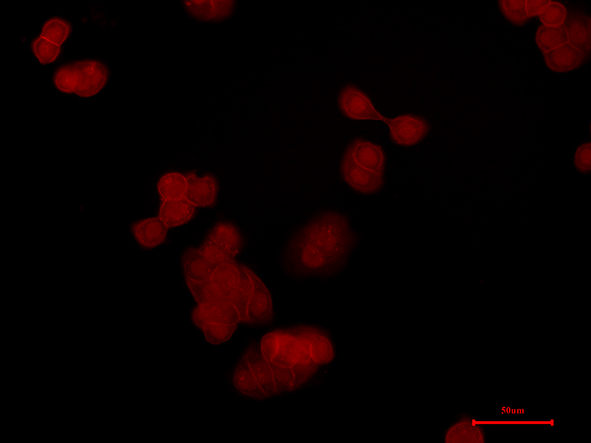

Supplement: Supplementary file 1 [file DataSheet1.ZIP › Data sources/Figure 2C, 2D, S3-IF/Figure S3-HT29/3-Claudin5/C0N/con-C5-2.jpg]

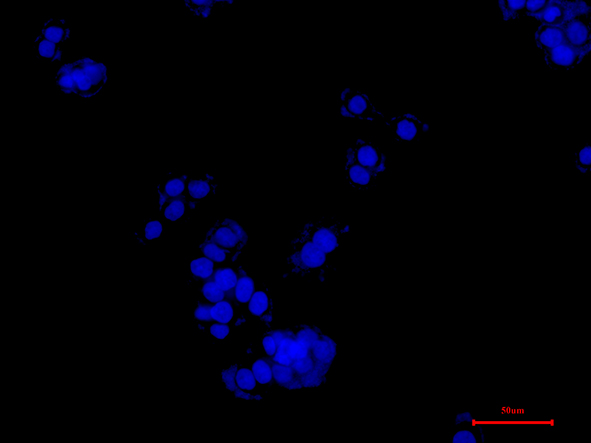

Supplement: Supplementary file 1 [file DataSheet1.ZIP › Data sources/Figure 2C, 2D, S3-IF/Figure S3-HT29/3-Claudin5/C0N/con-DAPI-2.jpg]

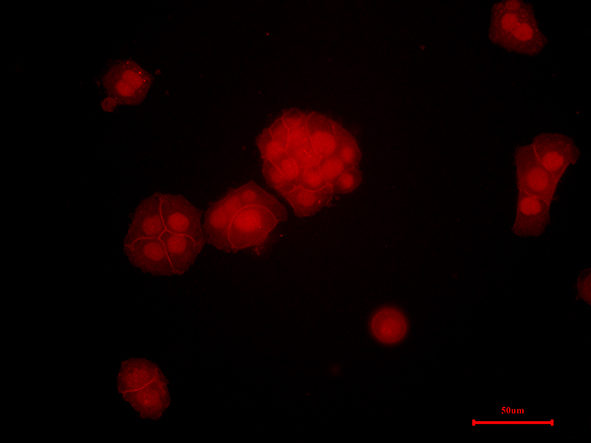

Supplement: Supplementary file 1 [file DataSheet1.ZIP › Data sources/Figure 2C, 2D, S3-IF/Figure S3-HT29/3-Claudin5/CON+buty/C5-2.jpg]

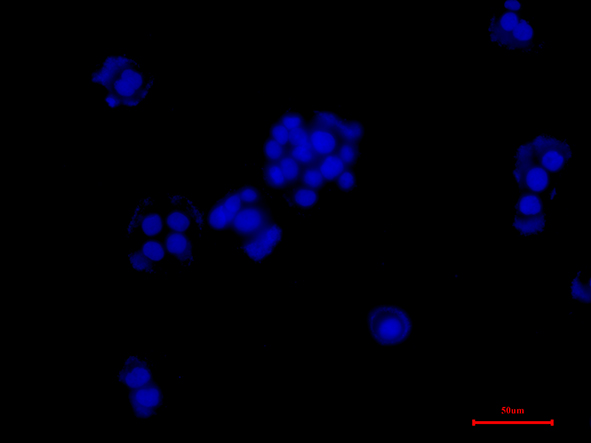

Supplement: Supplementary file 1 [file DataSheet1.ZIP › Data sources/Figure 2C, 2D, S3-IF/Figure S3-HT29/3-Claudin5/CON+buty/DAPI-2.jpg]

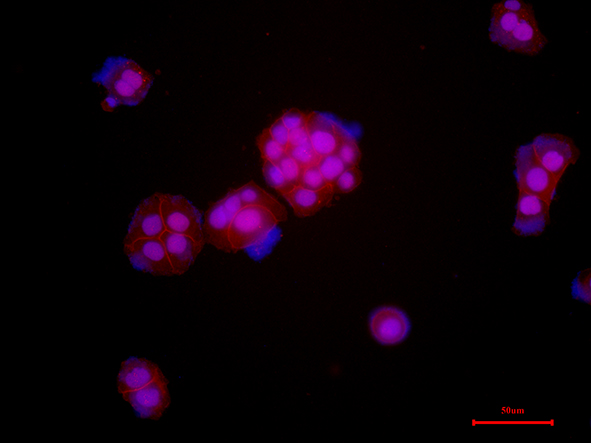

Supplement: Supplementary file 1 [file DataSheet1.ZIP › Data sources/Figure 2C, 2D, S3-IF/Figure S3-HT29/3-Claudin5/CON+buty/merge.jpg]
