# Supplementary figures and images for: Clostridium butyricum Protects Against Pancreatic and Intestinal Injury After Severe Acute Pancreatitis via Downregulation of MMP9 (part 1 of 2)
Source: Front Pharmacol. 2022 Jul 18;13:919010. doi: 10.3389/fphar.2022.919010 (PMC9342915; doi:10.3389/fphar.2022.919010)

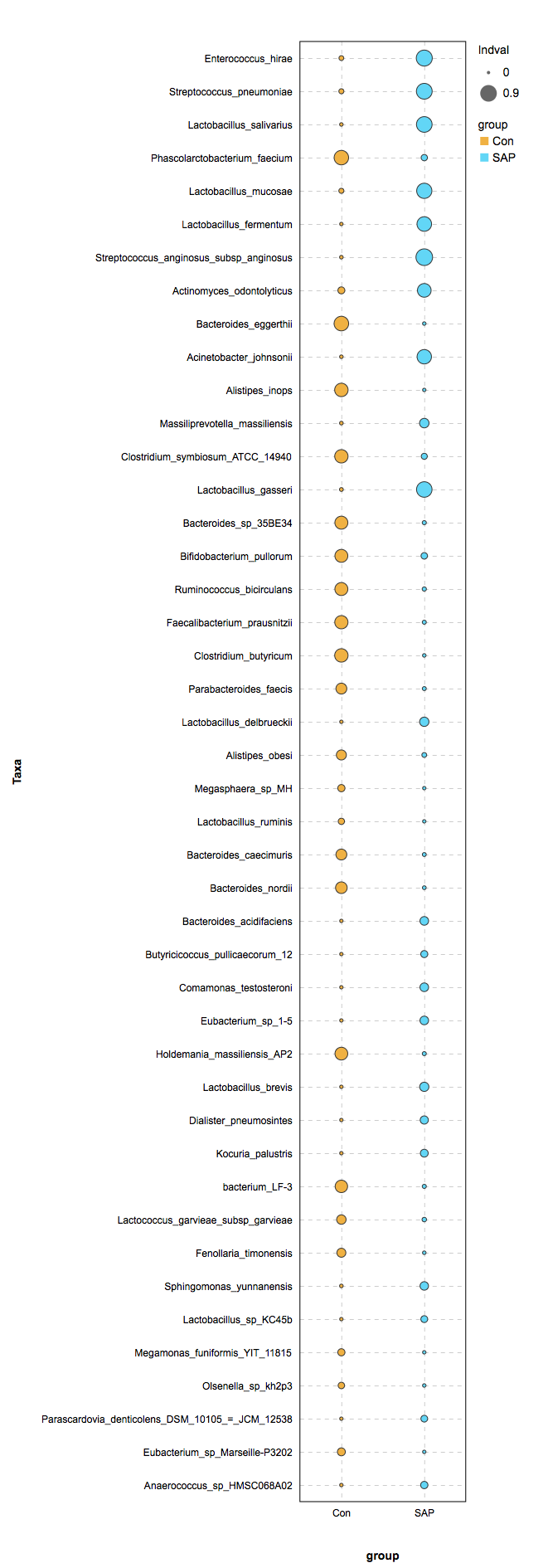

Supplement: Supplementary file 1 [file DataSheet1.ZIP › Data sources/Figure 1/Figure 1E-indicator.png]

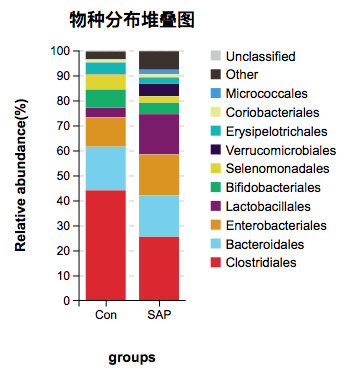

Supplement: Supplementary file 1 [file DataSheet1.ZIP › Data sources/Figure 1/Figure 1C- order.png]

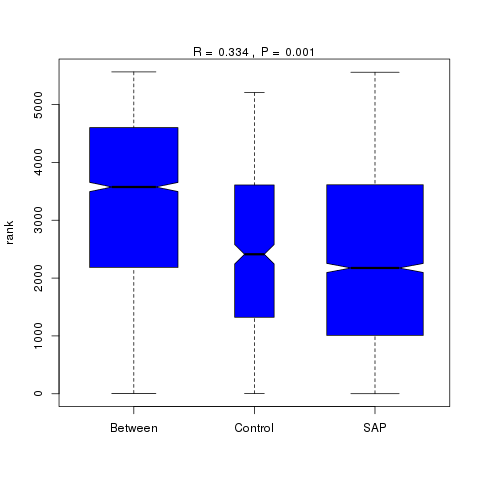

Supplement: Supplementary file 1 [file DataSheet1.ZIP › Data sources/Figure 1/Figure 1B-anosim.box.png]

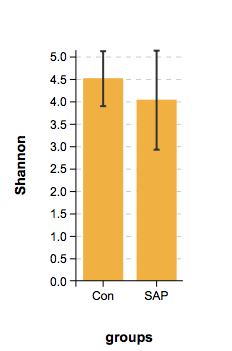

Supplement: Supplementary file 1 [file DataSheet1.ZIP › Data sources/Figure 1/Figure 1A-shannon.png]

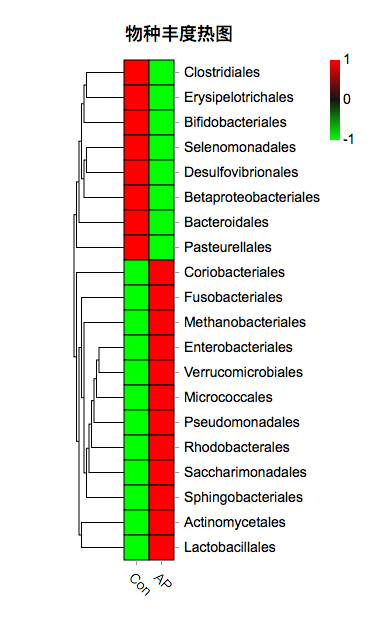

Supplement: Supplementary file 1 [file DataSheet1.ZIP › Data sources/Figure 1/Figure 1D-heatmap order.png]

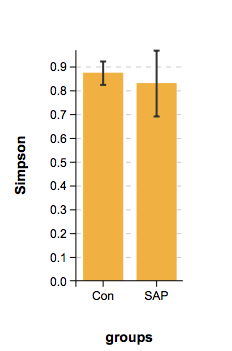

Supplement: Supplementary file 1 [file DataSheet1.ZIP › Data sources/Figure 1/Figure 1A-simpson .png]

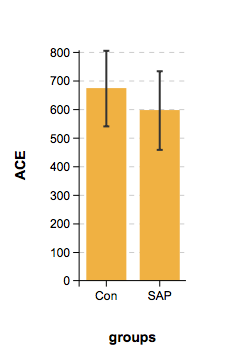

Supplement: Supplementary file 1 [file DataSheet1.ZIP › Data sources/Figure 1/Figure 1A-ace.png]

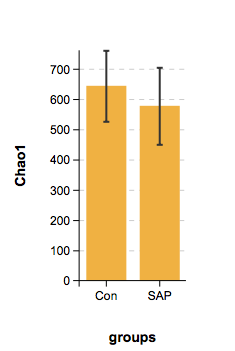

Supplement: Supplementary file 1 [file DataSheet1.ZIP › Data sources/Figure 1/Figure 1A-chao1.png]

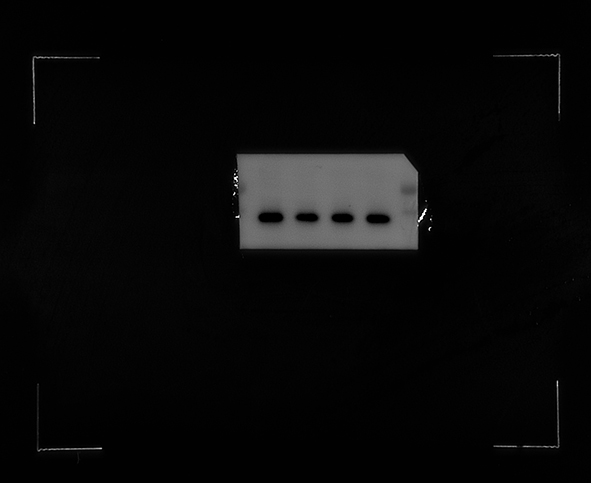

Supplement: Supplementary file 1 [file DataSheet1.ZIP › Data sources/Figure 2, 3, 4, 5, 6, 8-WB/Figure 8/Claudin5.jpg]

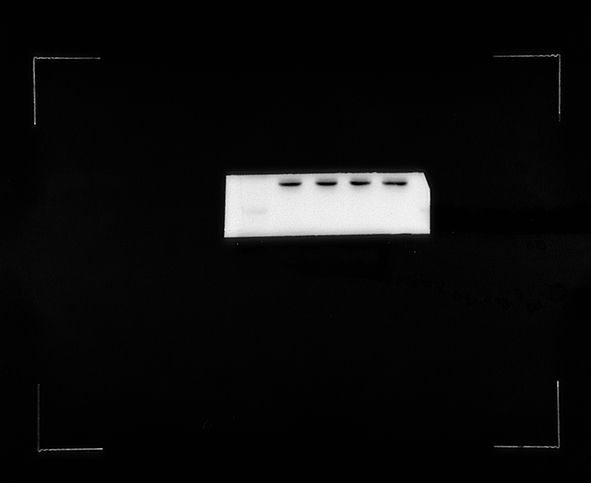

Supplement: Supplementary file 1 [file DataSheet1.ZIP › Data sources/Figure 2, 3, 4, 5, 6, 8-WB/Figure 8/P38.jpg]

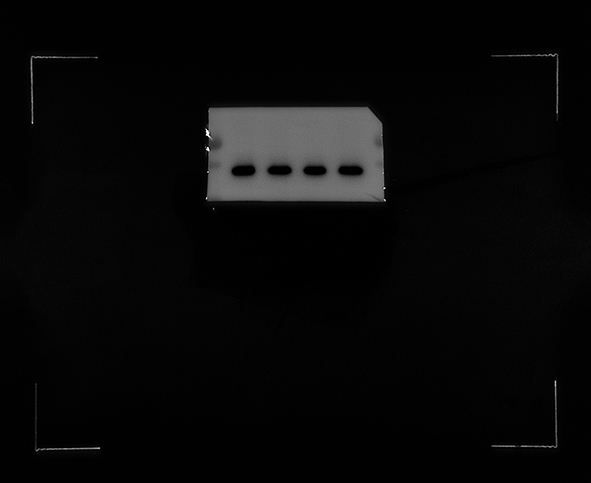

Supplement: Supplementary file 1 [file DataSheet1.ZIP › Data sources/Figure 2, 3, 4, 5, 6, 8-WB/Figure 8/Claudin3.jpg]

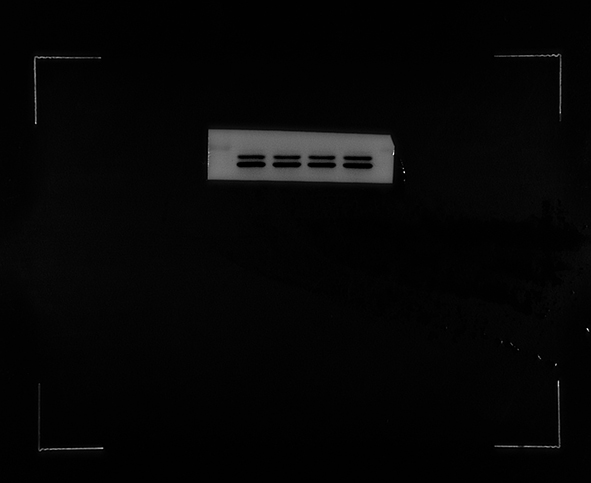

Supplement: Supplementary file 1 [file DataSheet1.ZIP › Data sources/Figure 2, 3, 4, 5, 6, 8-WB/Figure 8/ERK.jpg]

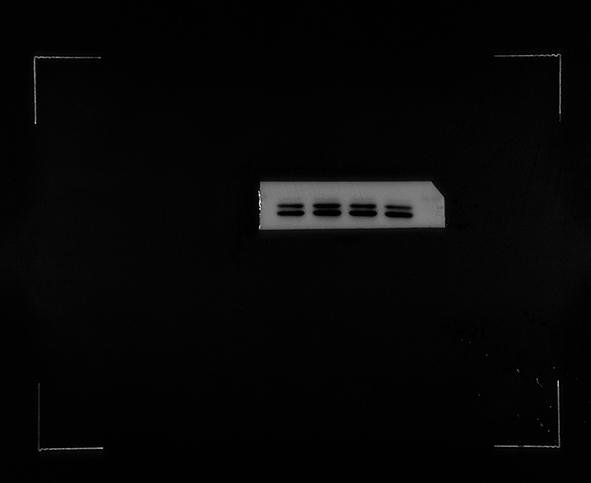

Supplement: Supplementary file 1 [file DataSheet1.ZIP › Data sources/Figure 2, 3, 4, 5, 6, 8-WB/Figure 8/p-ERK.jpg]

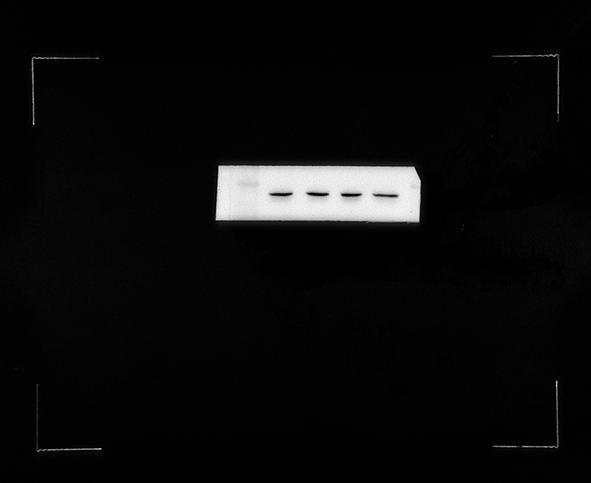

Supplement: Supplementary file 1 [file DataSheet1.ZIP › Data sources/Figure 2, 3, 4, 5, 6, 8-WB/Figure 8/Tubin.jpg]

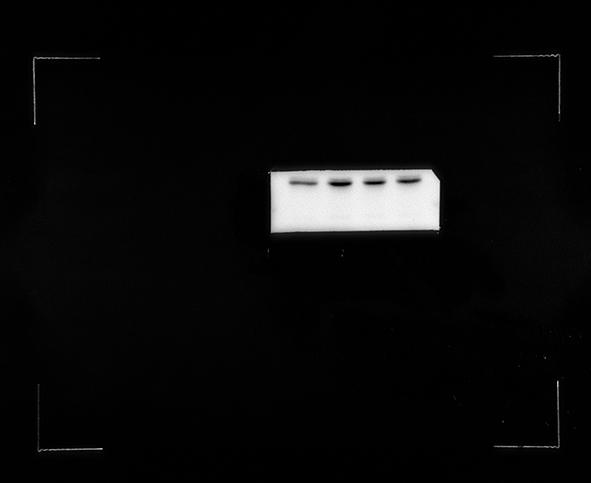

Supplement: Supplementary file 1 [file DataSheet1.ZIP › Data sources/Figure 2, 3, 4, 5, 6, 8-WB/Figure 8/p-P38.jpg]

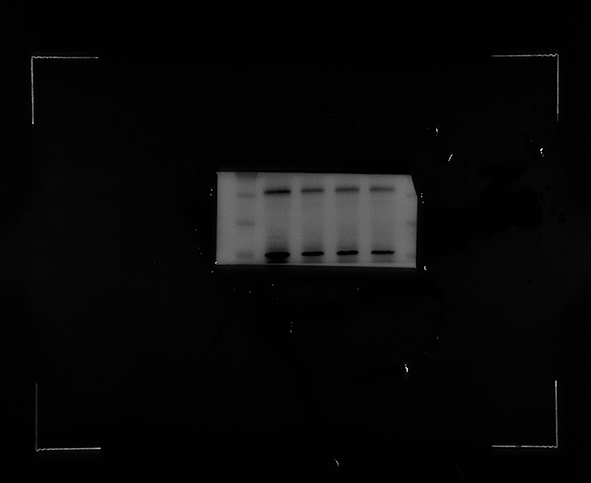

Supplement: Supplementary file 1 [file DataSheet1.ZIP › Data sources/Figure 2, 3, 4, 5, 6, 8-WB/Figure 8/Occludin.jpg]

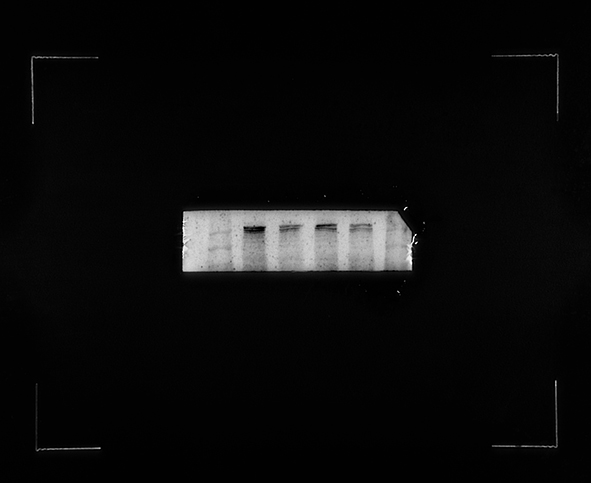

Supplement: Supplementary file 1 [file DataSheet1.ZIP › Data sources/Figure 2, 3, 4, 5, 6, 8-WB/Figure 8/ZO-1.jpg]

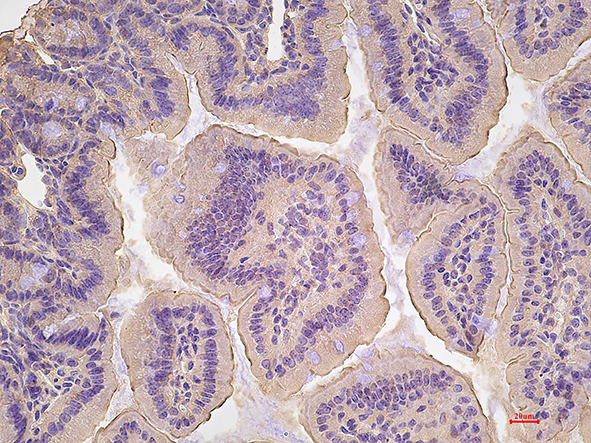

Supplement: Supplementary file 1 [file DataSheet1.ZIP › Data sources/Figure 8C-IHC/1-ZO-1/SAP-Buty.jpg]

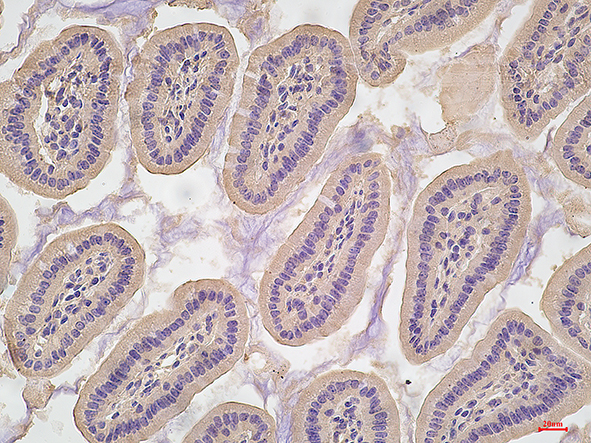

Supplement: Supplementary file 1 [file DataSheet1.ZIP › Data sources/Figure 8C-IHC/1-ZO-1/SAP.jpg]

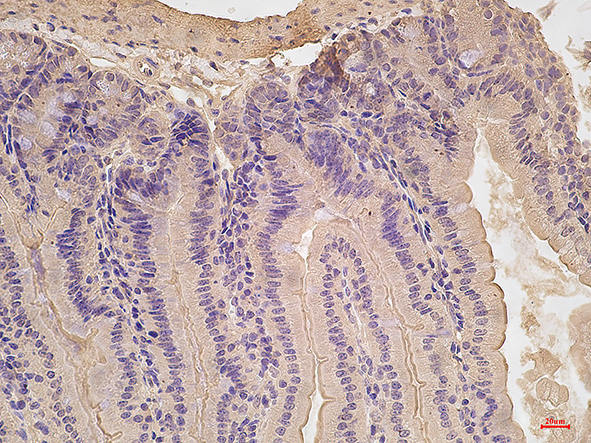

Supplement: Supplementary file 1 [file DataSheet1.ZIP › Data sources/Figure 8C-IHC/1-ZO-1/Control.jpg]

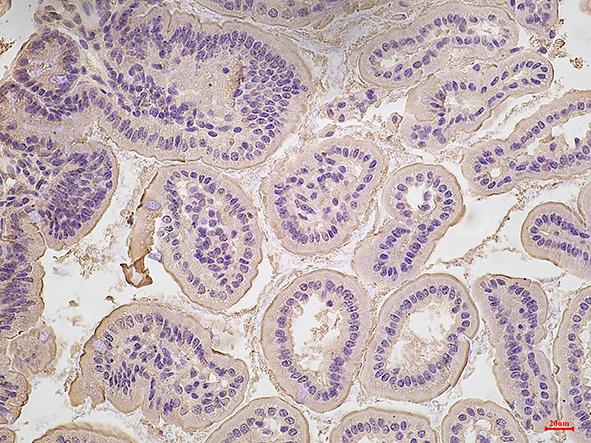

Supplement: Supplementary file 1 [file DataSheet1.ZIP › Data sources/Figure 8C-IHC/1-ZO-1/SAP-C.buty.jpg]

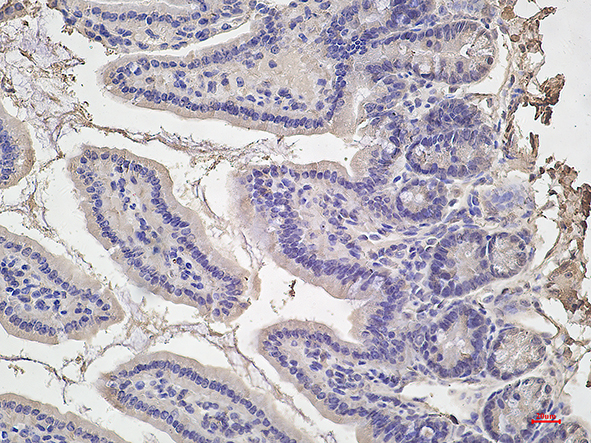

Supplement: Supplementary file 1 [file DataSheet1.ZIP › Data sources/Figure 8C-IHC/2-occludin1/SAP.jpg]

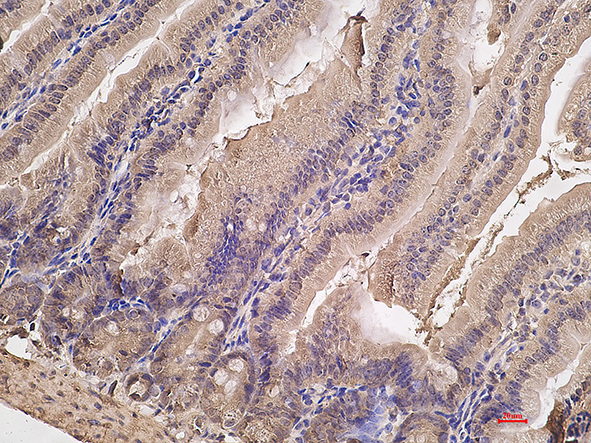

Supplement: Supplementary file 1 [file DataSheet1.ZIP › Data sources/Figure 8C-IHC/2-occludin1/Cont.jpg]

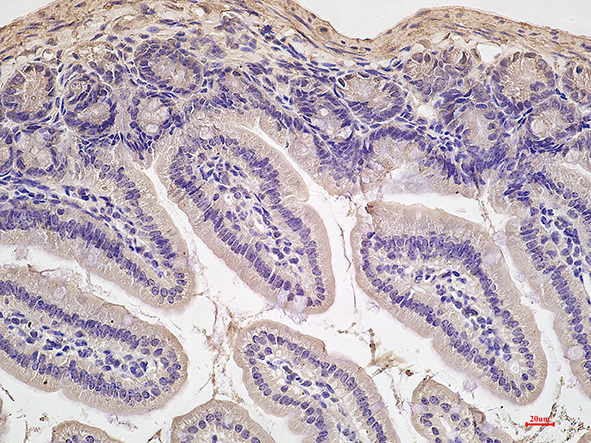

Supplement: Supplementary file 1 [file DataSheet1.ZIP › Data sources/Figure 8C-IHC/2-occludin1/SAP+buty.jpg]

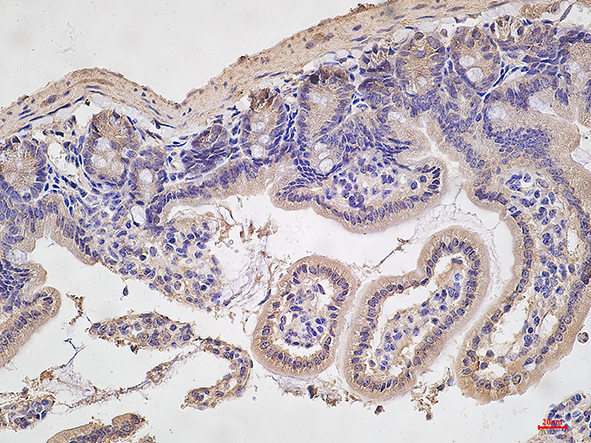

Supplement: Supplementary file 1 [file DataSheet1.ZIP › Data sources/Figure 8C-IHC/2-occludin1/SAP+C.buty.jpg]

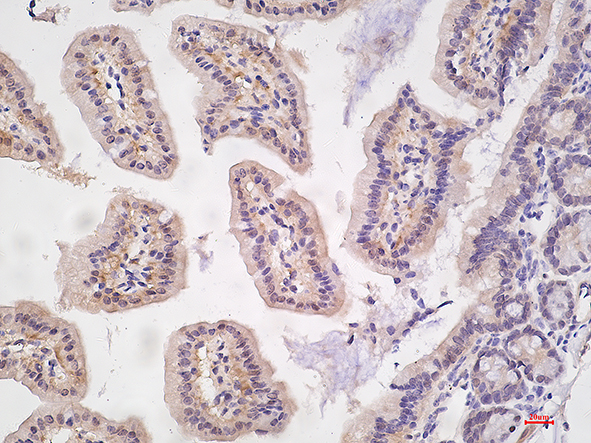

Supplement: Supplementary file 1 [file DataSheet1.ZIP › Data sources/Figure 8C-IHC/3-claudin3/SAP.jpg]

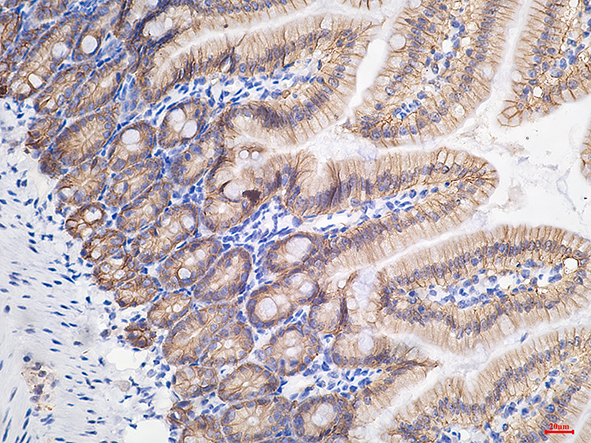

Supplement: Supplementary file 1 [file DataSheet1.ZIP › Data sources/Figure 8C-IHC/3-claudin3/Cont.jpg]

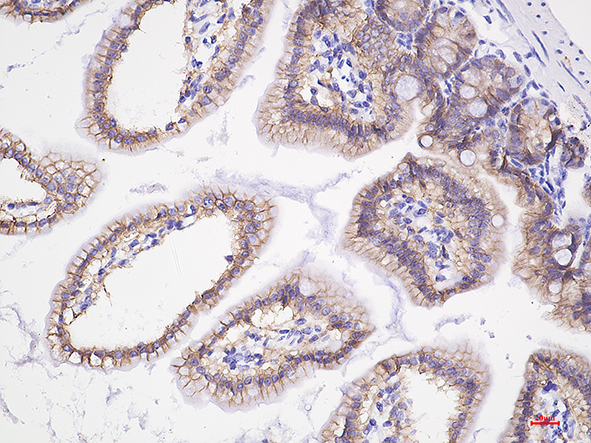

Supplement: Supplementary file 1 [file DataSheet1.ZIP › Data sources/Figure 8C-IHC/3-claudin3/SAP+buty.jpg]

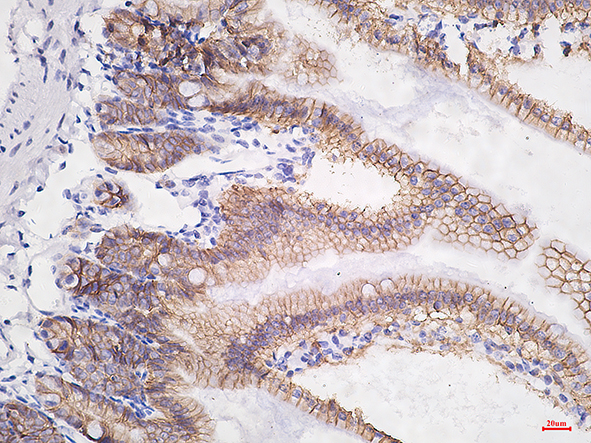

Supplement: Supplementary file 1 [file DataSheet1.ZIP › Data sources/Figure 8C-IHC/3-claudin3/SAP+C.buty.jpg]

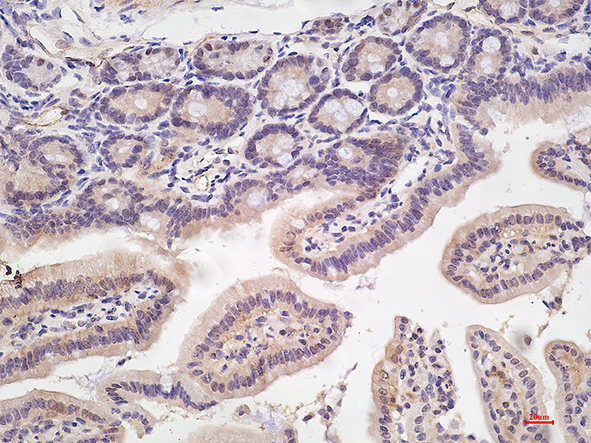

Supplement: Supplementary file 1 [file DataSheet1.ZIP › Data sources/Figure 8C-IHC/4-claudin5/SAP.jpg]

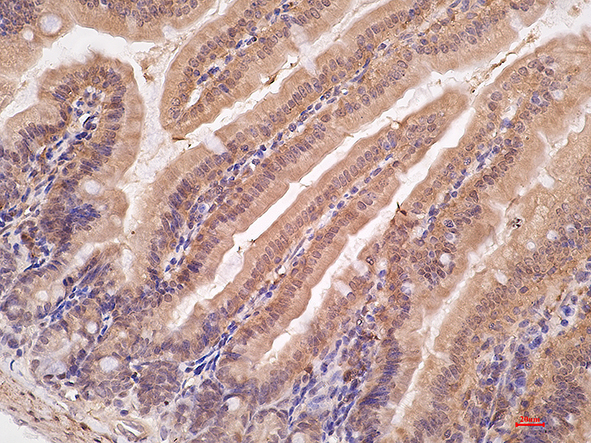

Supplement: Supplementary file 1 [file DataSheet1.ZIP › Data sources/Figure 8C-IHC/4-claudin5/Cont.jpg]

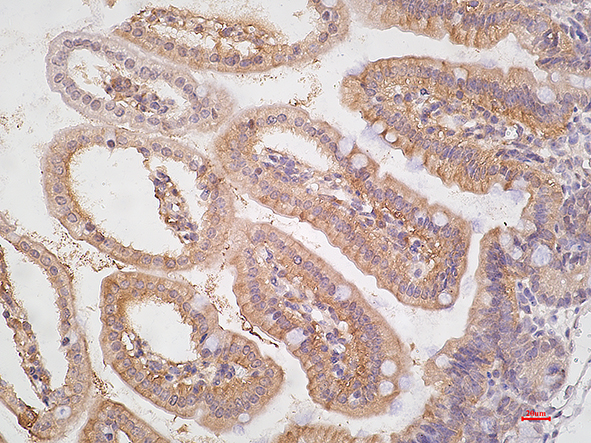

Supplement: Supplementary file 1 [file DataSheet1.ZIP › Data sources/Figure 8C-IHC/4-claudin5/SAP+Buty.jpg]

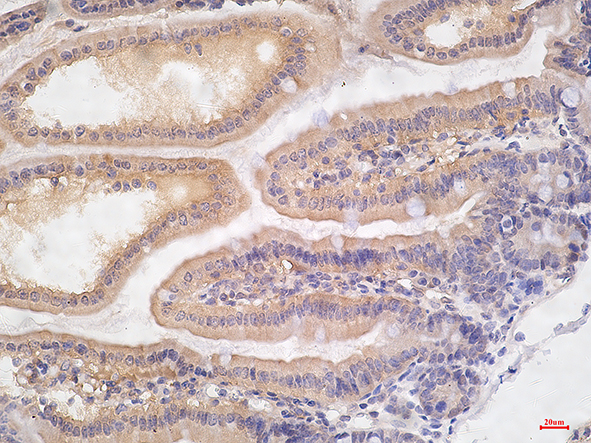

Supplement: Supplementary file 1 [file DataSheet1.ZIP › Data sources/Figure 8C-IHC/4-claudin5/SAP+C.Buty.jpg]

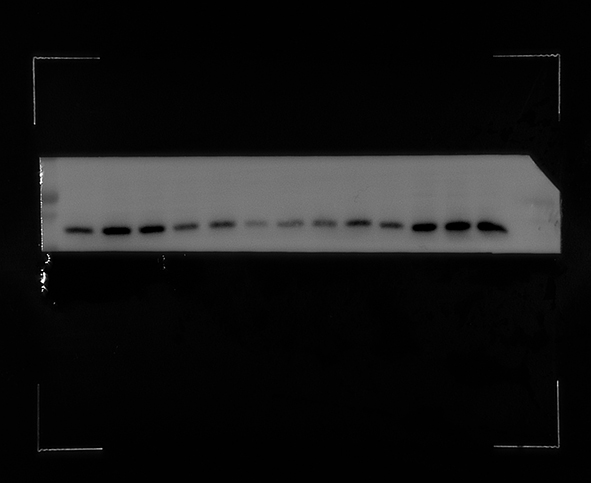

Supplement: Supplementary file 1 [file DataSheet1.ZIP › Data sources/Figure 2, 3, 4, 5, 6, 8-WB/Figure 3,5,6/Caco2/Claudin5.jpg]

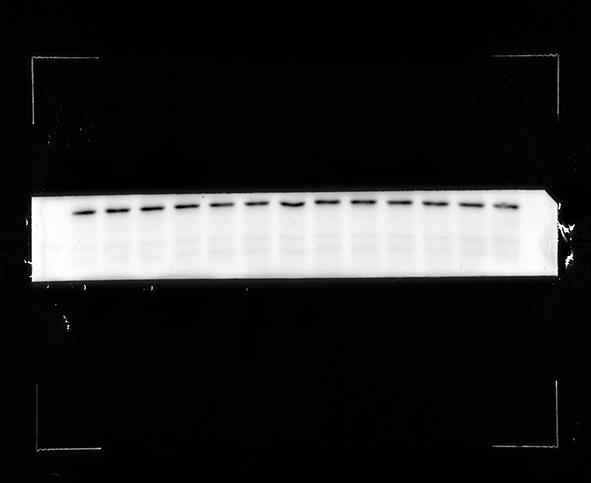

Supplement: Supplementary file 1 [file DataSheet1.ZIP › Data sources/Figure 2, 3, 4, 5, 6, 8-WB/Figure 3,5,6/Caco2/P38.jpg]

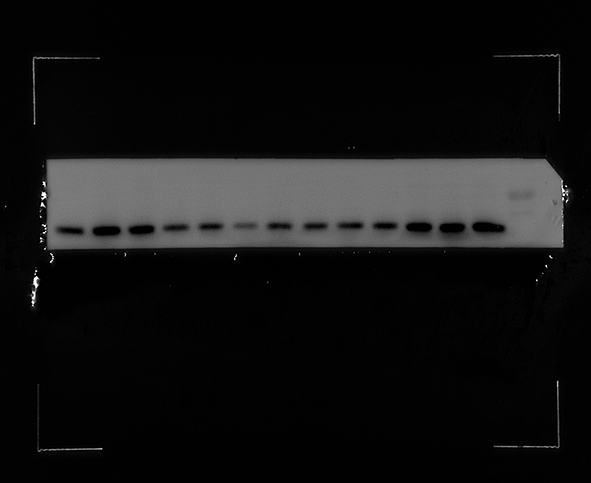

Supplement: Supplementary file 1 [file DataSheet1.ZIP › Data sources/Figure 2, 3, 4, 5, 6, 8-WB/Figure 3,5,6/Caco2/Claudin3.jpg]

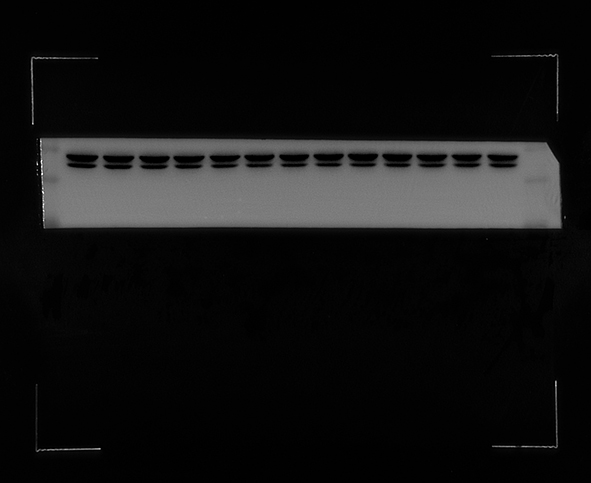

Supplement: Supplementary file 1 [file DataSheet1.ZIP › Data sources/Figure 2, 3, 4, 5, 6, 8-WB/Figure 3,5,6/Caco2/ERK.jpg]

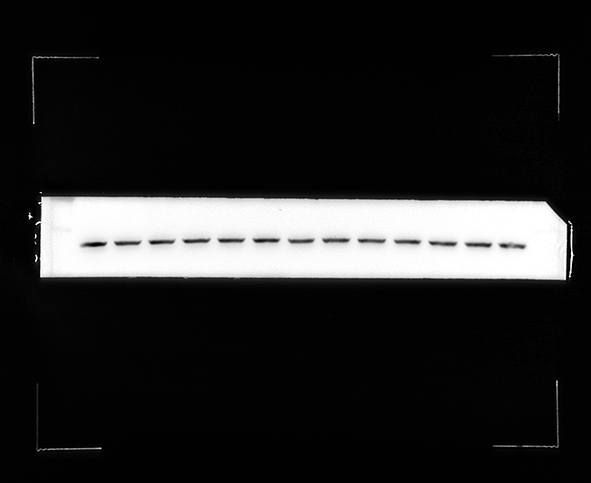

Supplement: Supplementary file 1 [file DataSheet1.ZIP › Data sources/Figure 2, 3, 4, 5, 6, 8-WB/Figure 3,5,6/Caco2/a-Tublin.jpg]

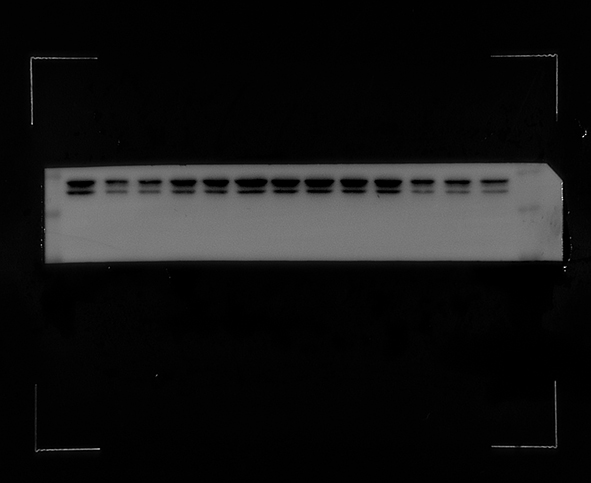

Supplement: Supplementary file 1 [file DataSheet1.ZIP › Data sources/Figure 2, 3, 4, 5, 6, 8-WB/Figure 3,5,6/Caco2/p-ERK.jpg]

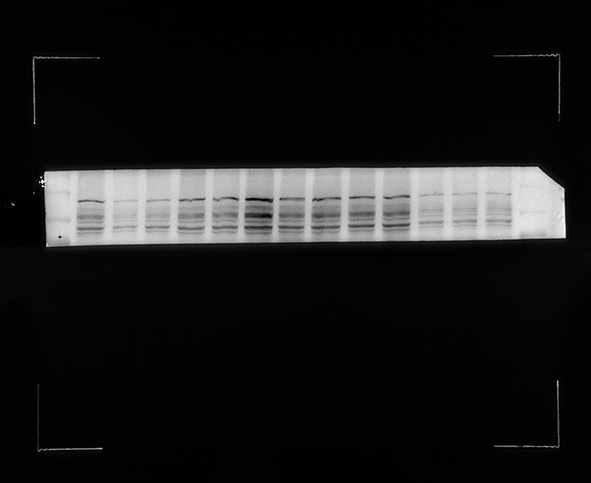

Supplement: Supplementary file 1 [file DataSheet1.ZIP › Data sources/Figure 2, 3, 4, 5, 6, 8-WB/Figure 3,5,6/Caco2/MMP-9.jpg]

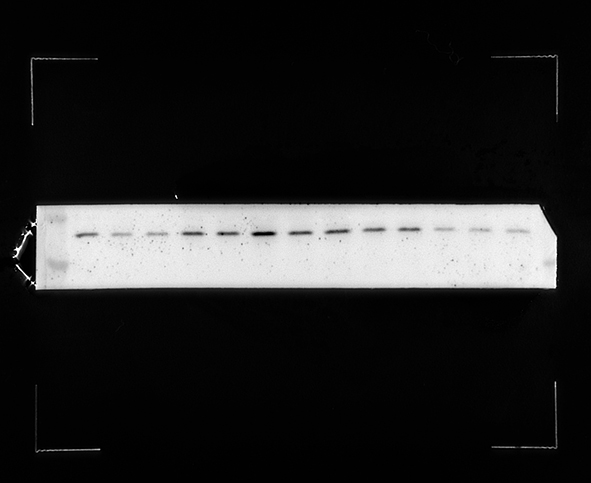

Supplement: Supplementary file 1 [file DataSheet1.ZIP › Data sources/Figure 2, 3, 4, 5, 6, 8-WB/Figure 3,5,6/Caco2/p-P38.jpg]

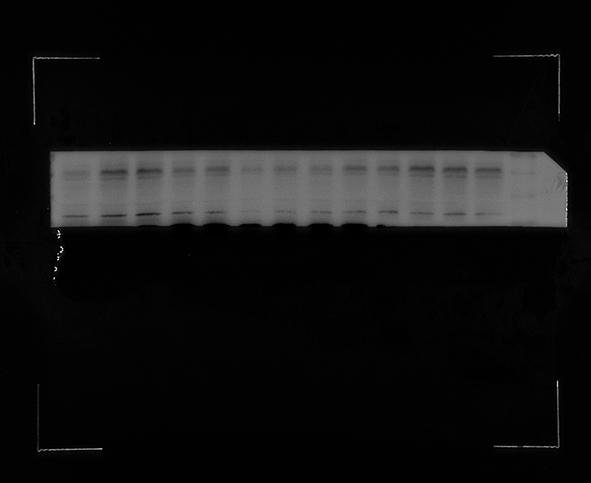

Supplement: Supplementary file 1 [file DataSheet1.ZIP › Data sources/Figure 2, 3, 4, 5, 6, 8-WB/Figure 3,5,6/Caco2/Occludin.jpg]

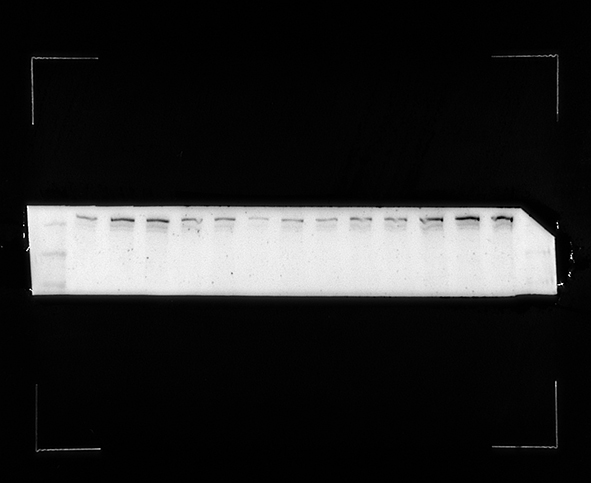

Supplement: Supplementary file 1 [file DataSheet1.ZIP › Data sources/Figure 2, 3, 4, 5, 6, 8-WB/Figure 3,5,6/Caco2/ZO-1.jpg]

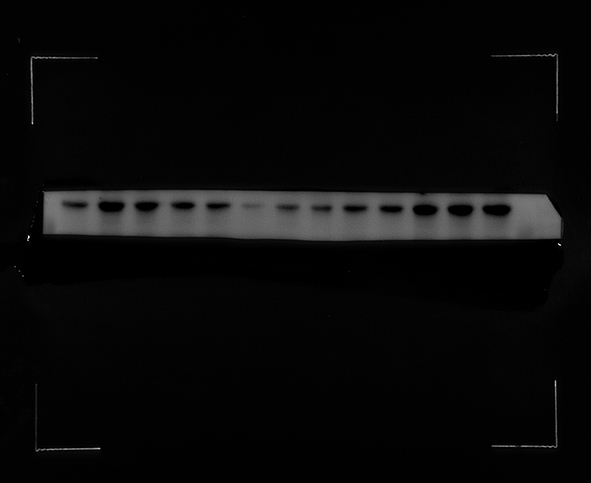

Supplement: Supplementary file 1 [file DataSheet1.ZIP › Data sources/Figure 2, 3, 4, 5, 6, 8-WB/Figure 3,5,6/HT29/Claudin5.jpg]

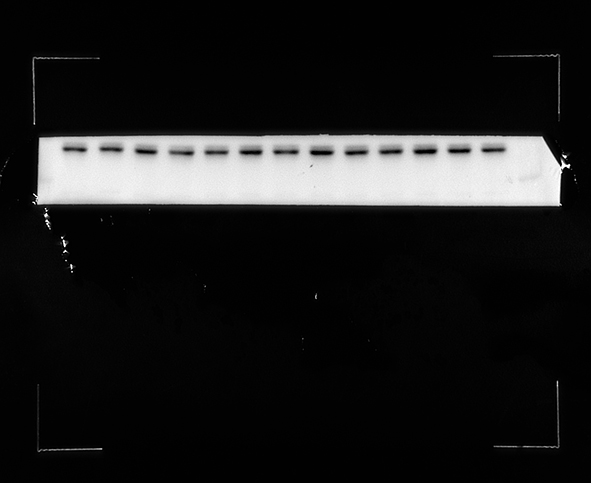

Supplement: Supplementary file 1 [file DataSheet1.ZIP › Data sources/Figure 2, 3, 4, 5, 6, 8-WB/Figure 3,5,6/HT29/P38.jpg]

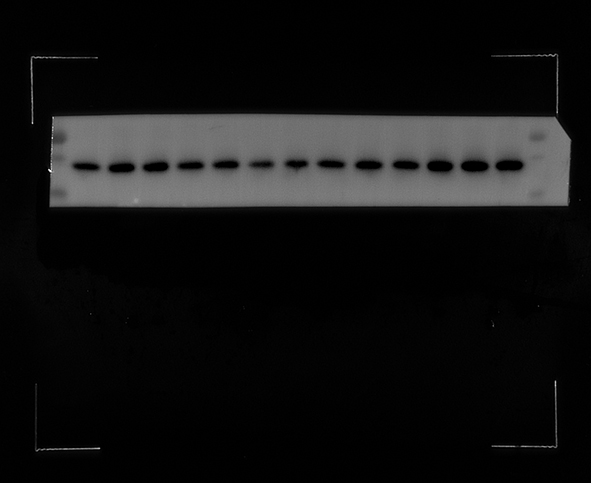

Supplement: Supplementary file 1 [file DataSheet1.ZIP › Data sources/Figure 2, 3, 4, 5, 6, 8-WB/Figure 3,5,6/HT29/Claudin3.jpg]

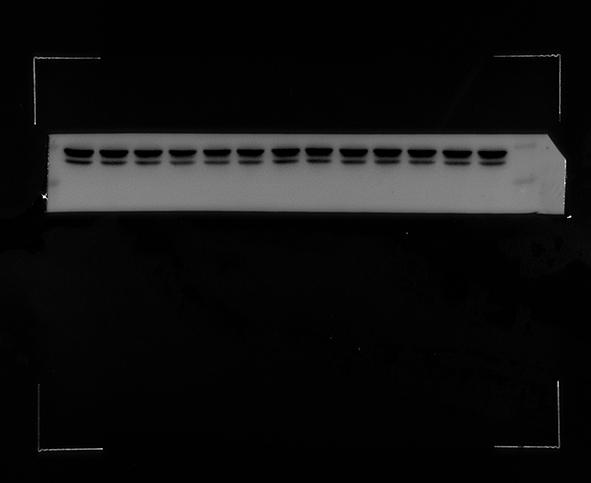

Supplement: Supplementary file 1 [file DataSheet1.ZIP › Data sources/Figure 2, 3, 4, 5, 6, 8-WB/Figure 3,5,6/HT29/ERK.jpg]

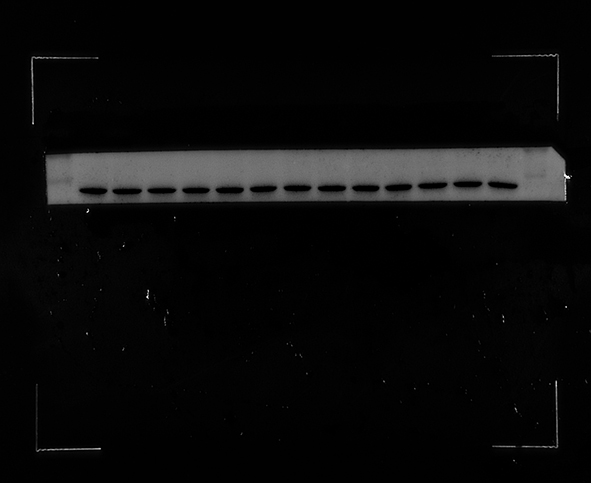

Supplement: Supplementary file 1 [file DataSheet1.ZIP › Data sources/Figure 2, 3, 4, 5, 6, 8-WB/Figure 3,5,6/HT29/Tublin.jpg]

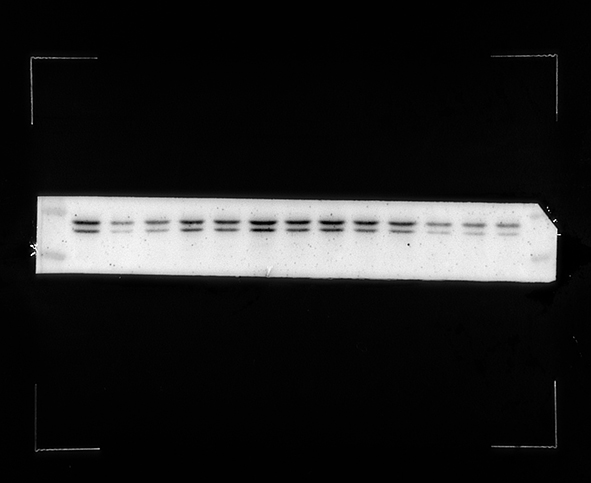

Supplement: Supplementary file 1 [file DataSheet1.ZIP › Data sources/Figure 2, 3, 4, 5, 6, 8-WB/Figure 3,5,6/HT29/p-ERK.jpg]

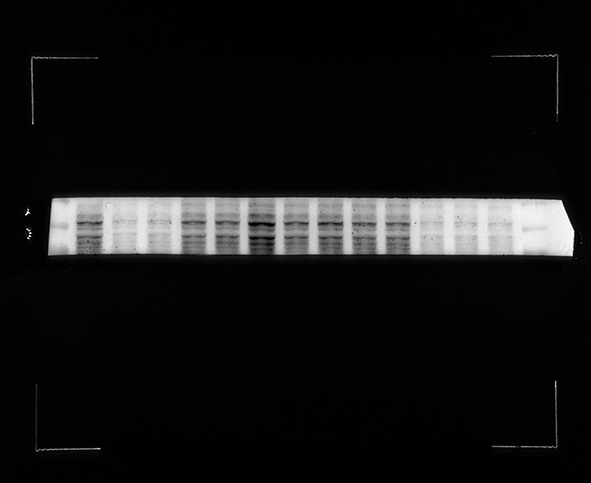

Supplement: Supplementary file 1 [file DataSheet1.ZIP › Data sources/Figure 2, 3, 4, 5, 6, 8-WB/Figure 3,5,6/HT29/MMP-9.jpg]

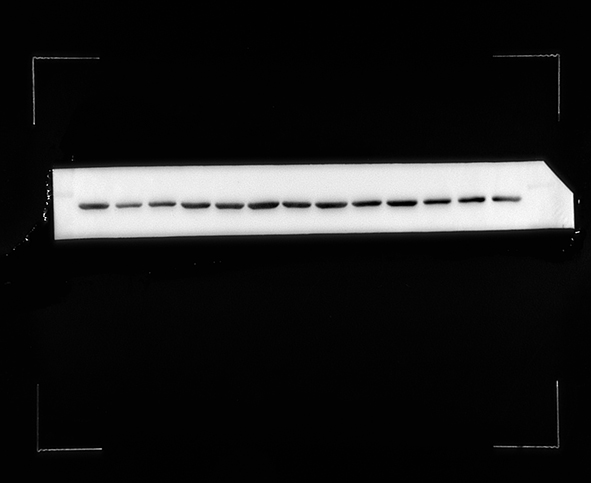

Supplement: Supplementary file 1 [file DataSheet1.ZIP › Data sources/Figure 2, 3, 4, 5, 6, 8-WB/Figure 3,5,6/HT29/p-P38.jpg]

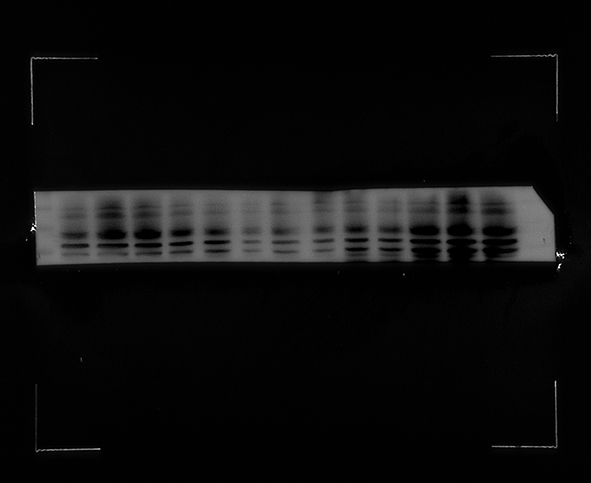

Supplement: Supplementary file 1 [file DataSheet1.ZIP › Data sources/Figure 2, 3, 4, 5, 6, 8-WB/Figure 3,5,6/HT29/Occludin.jpg]

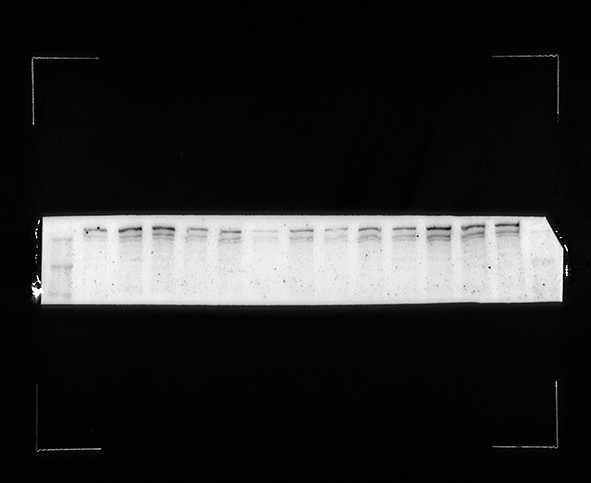

Supplement: Supplementary file 1 [file DataSheet1.ZIP › Data sources/Figure 2, 3, 4, 5, 6, 8-WB/Figure 3,5,6/HT29/ZO-1.jpg]

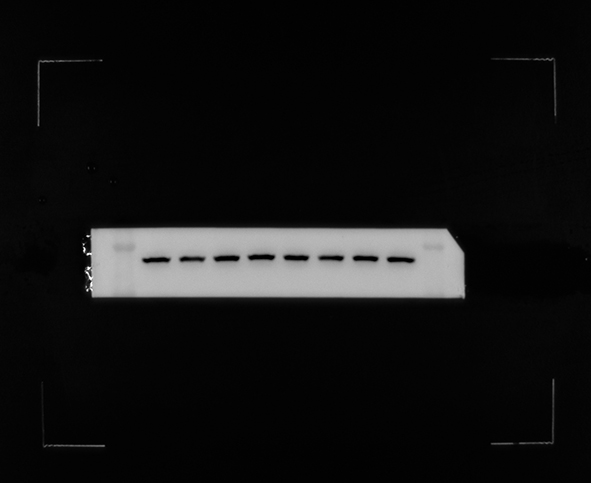

Supplement: Supplementary file 1 [file DataSheet1.ZIP › Data sources/Figure 2, 3, 4, 5, 6, 8-WB/Figure 4/Figure 4A,C-Caco-2 cell/Tublin.jpg]

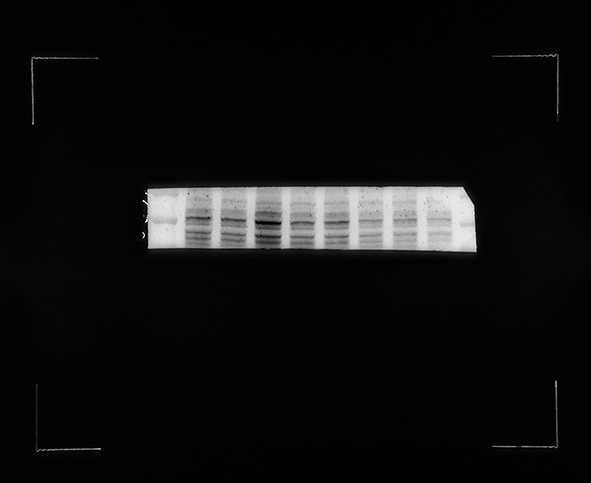

Supplement: Supplementary file 1 [file DataSheet1.ZIP › Data sources/Figure 2, 3, 4, 5, 6, 8-WB/Figure 4/Figure 4A,C-Caco-2 cell/MMP-9.jpg]

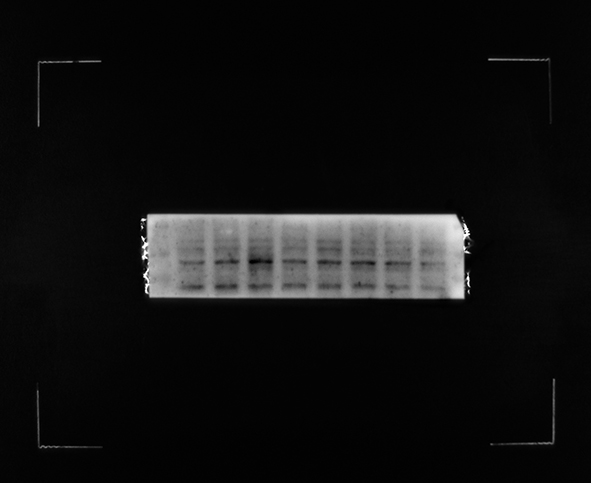

Supplement: Supplementary file 1 [file DataSheet1.ZIP › Data sources/Figure 2, 3, 4, 5, 6, 8-WB/Figure 4/Figure 4B,C-HT-29 cell/MMP-9.jpg]

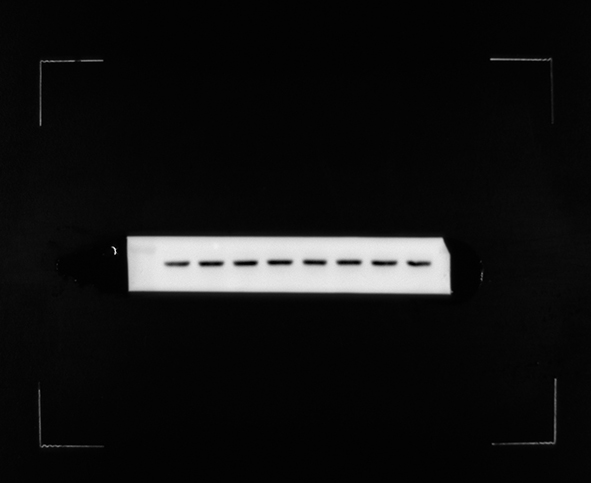

Supplement: Supplementary file 1 [file DataSheet1.ZIP › Data sources/Figure 2, 3, 4, 5, 6, 8-WB/Figure 4/Figure 4B,C-HT-29 cell/a-Tubulin.jpg]

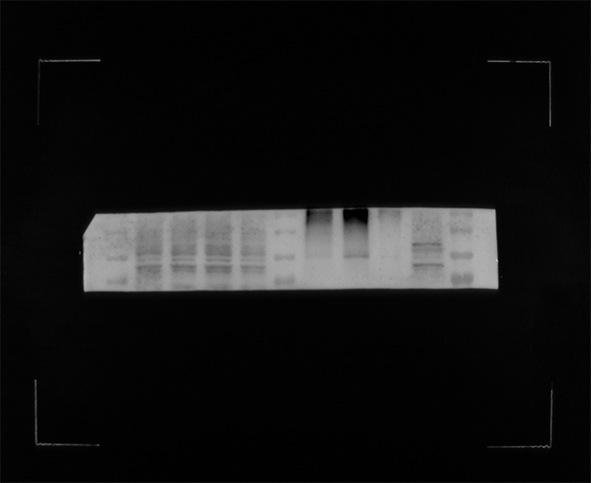

Supplement: Supplementary file 1 [file DataSheet1.ZIP › Data sources/Figure 2, 3, 4, 5, 6, 8-WB/Figure 2/Figure 2A-Caco2 cell/MMP-9 (left).jpg]

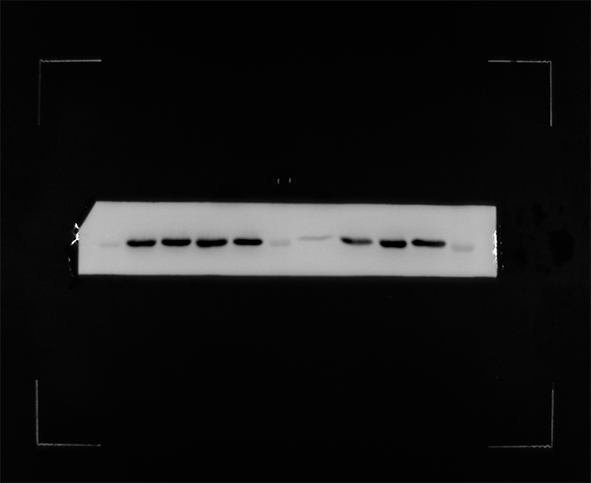

Supplement: Supplementary file 1 [file DataSheet1.ZIP › Data sources/Figure 2, 3, 4, 5, 6, 8-WB/Figure 2/Figure 2A-Caco2 cell/GAPDH (left).jpg]

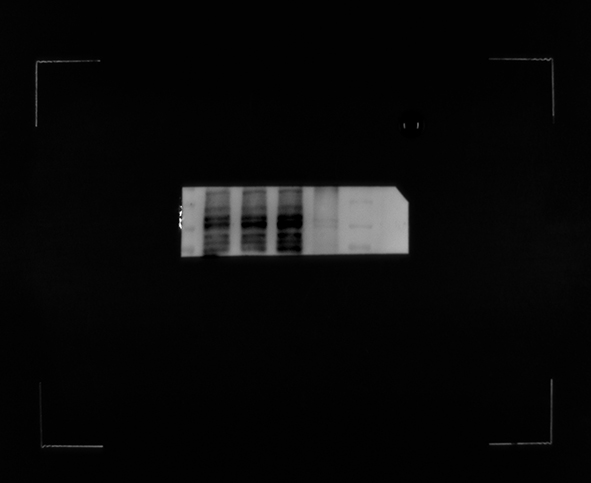

Supplement: Supplementary file 1 [file DataSheet1.ZIP › Data sources/Figure 2, 3, 4, 5, 6, 8-WB/Figure 2/Figure 2B-HT29 cell/MMP-9.jpg]

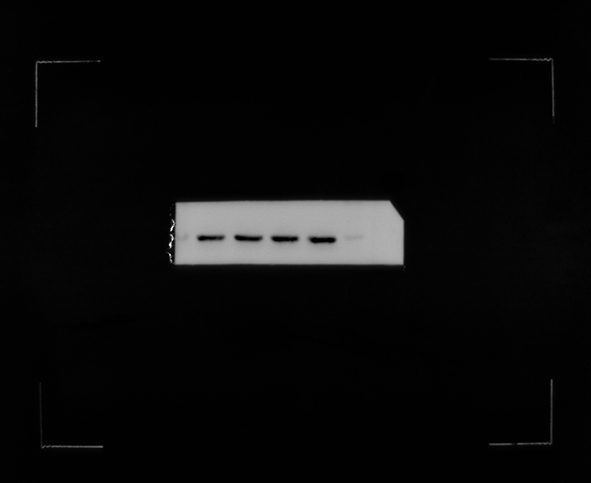

Supplement: Supplementary file 1 [file DataSheet1.ZIP › Data sources/Figure 2, 3, 4, 5, 6, 8-WB/Figure 2/Figure 2B-HT29 cell/GAPDH.jpg]

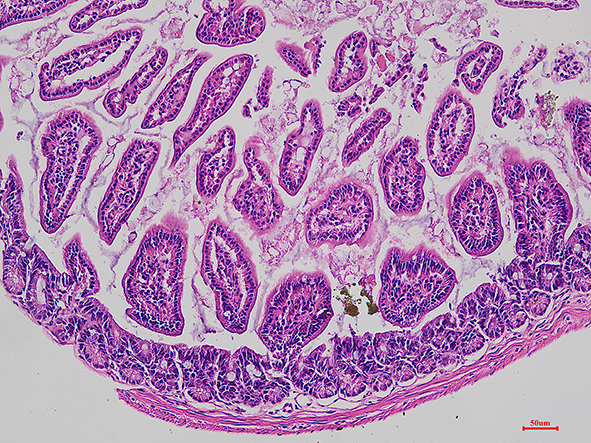

Supplement: Supplementary file 1 [file DataSheet1.ZIP › Data sources/Figure 7/Figure 7A-HE/Intestines/2-SAP.jpg]

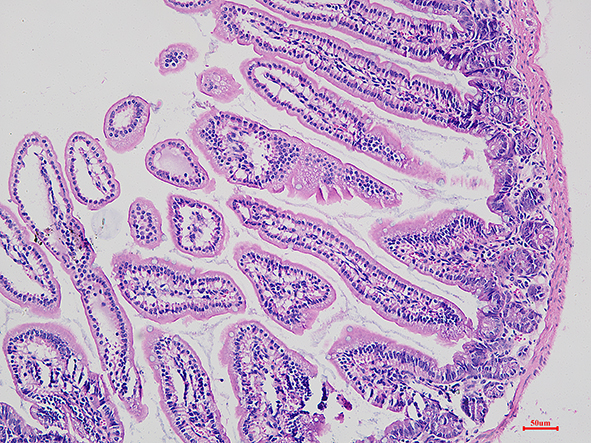

Supplement: Supplementary file 1 [file DataSheet1.ZIP › Data sources/Figure 7/Figure 7A-HE/Intestines/4-SAP+buty.jpg]

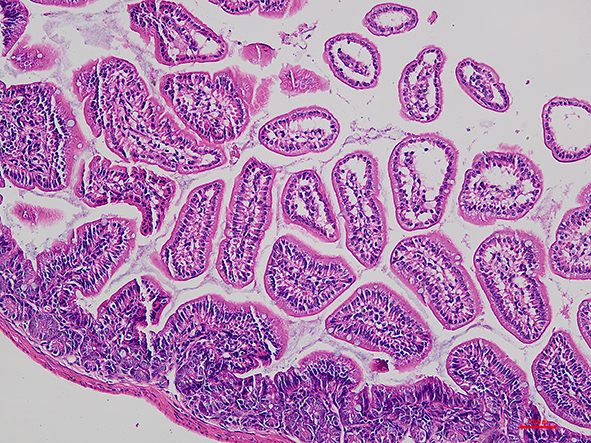

Supplement: Supplementary file 1 [file DataSheet1.ZIP › Data sources/Figure 7/Figure 7A-HE/Intestines/3-SAP+C.buty.jpg]

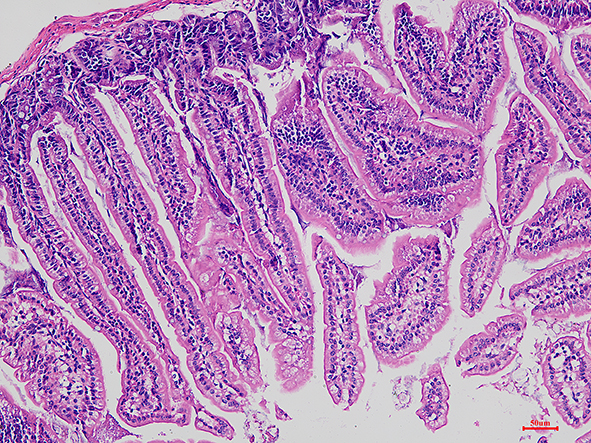

Supplement: Supplementary file 1 [file DataSheet1.ZIP › Data sources/Figure 7/Figure 7A-HE/Intestines/1-Con.jpg]

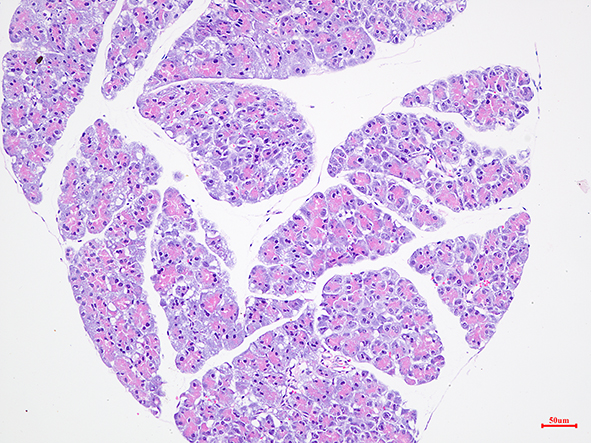

Supplement: Supplementary file 1 [file DataSheet1.ZIP › Data sources/Figure 7/Figure 7A-HE/Pancreas/2-SAP.jpg]

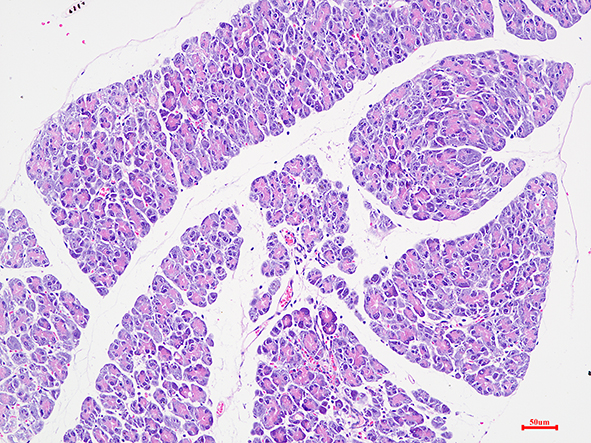

Supplement: Supplementary file 1 [file DataSheet1.ZIP › Data sources/Figure 7/Figure 7A-HE/Pancreas/4-SAP+buty.jpg]

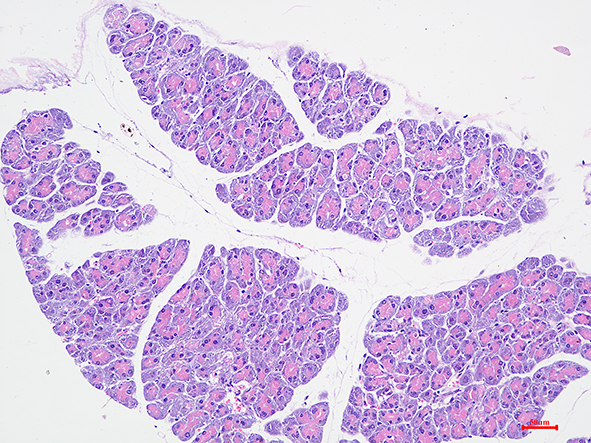

Supplement: Supplementary file 1 [file DataSheet1.ZIP › Data sources/Figure 7/Figure 7A-HE/Pancreas/3-SAP+C.buty.jpg]

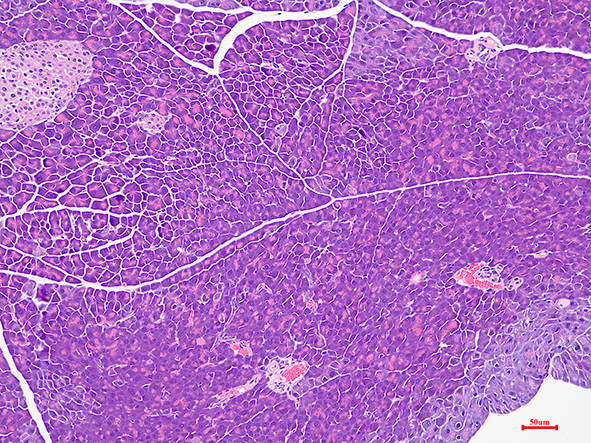

Supplement: Supplementary file 1 [file DataSheet1.ZIP › Data sources/Figure 7/Figure 7A-HE/Pancreas/1-con.jpg]

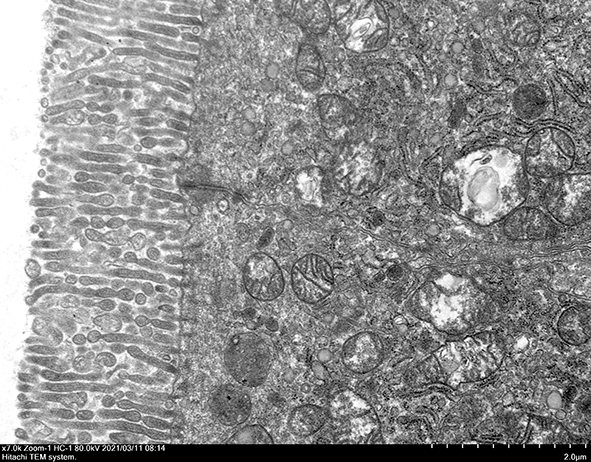

Supplement: Supplementary file 1 [file DataSheet1.ZIP › Data sources/Figure 7/Figure 7C-TEM/4-SAP+Buty/SAP + Buty.jpg]

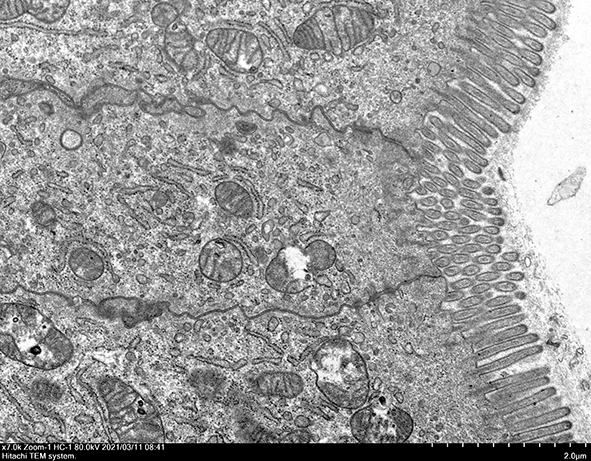

Supplement: Supplementary file 1 [file DataSheet1.ZIP › Data sources/Figure 7/Figure 7C-TEM/2-SAP/SAP.jpg]

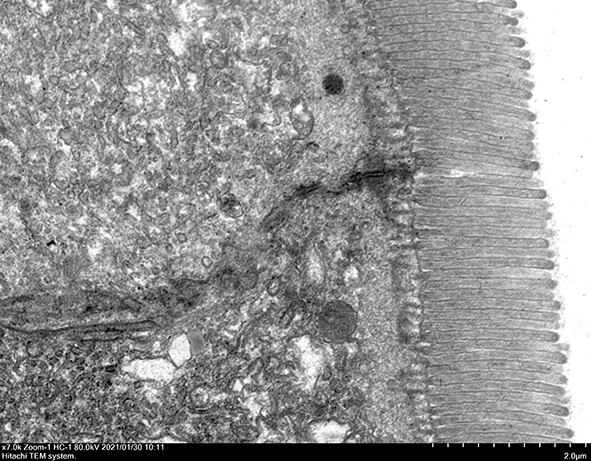

Supplement: Supplementary file 1 [file DataSheet1.ZIP › Data sources/Figure 7/Figure 7C-TEM/1-Control/control.jpg]

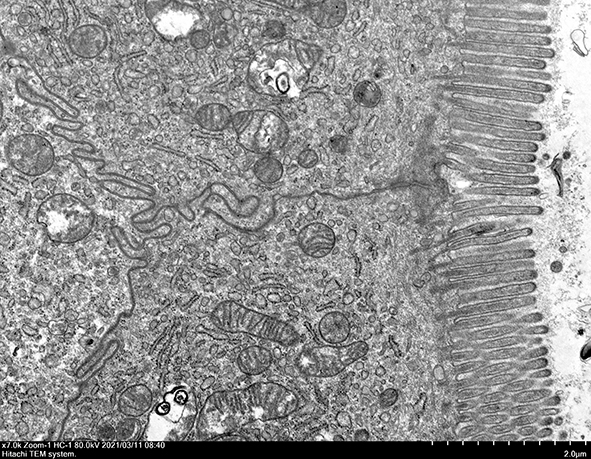

Supplement: Supplementary file 1 [file DataSheet1.ZIP › Data sources/Figure 7/Figure 7C-TEM/3-SAP+C.buty/SAP + C.buty.jpg]

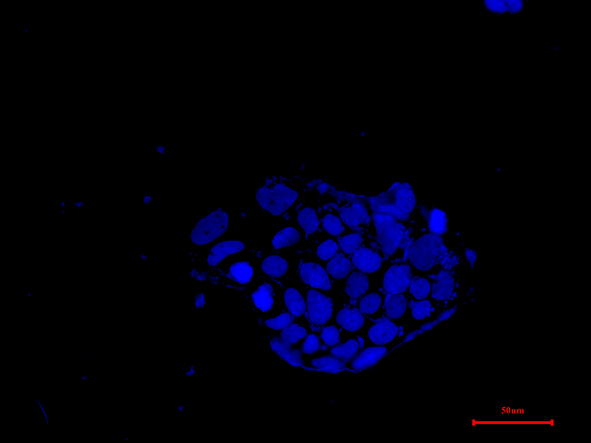

Supplement: Supplementary file 1 [file DataSheet1.ZIP › Data sources/Figure 2C, 2D, S3-IF/Figure 2C,D-Caco2/1-ZO-1/C. Butyricum/C.buty-DAPI.jpg]

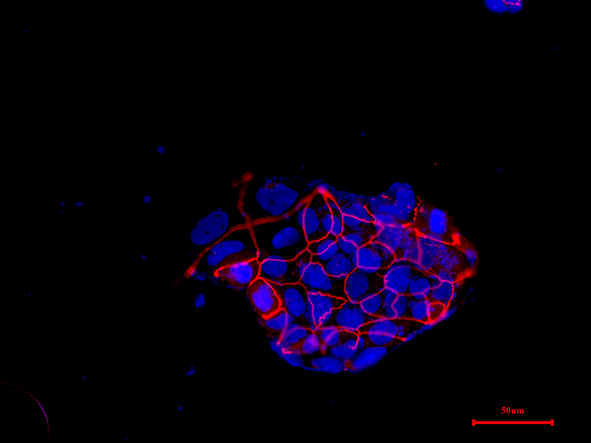

Supplement: Supplementary file 1 [file DataSheet1.ZIP › Data sources/Figure 2C, 2D, S3-IF/Figure 2C,D-Caco2/1-ZO-1/C. Butyricum/merge.jpg]

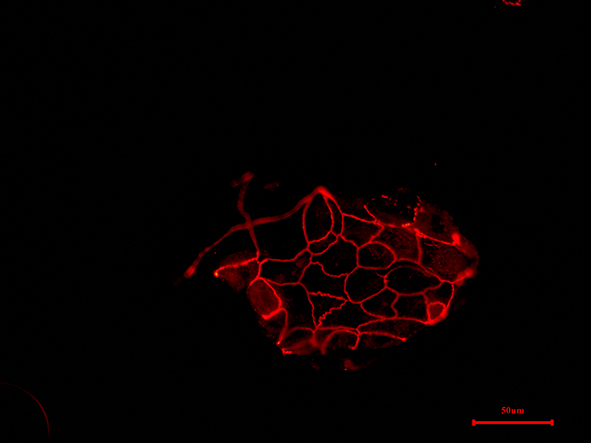

Supplement: Supplementary file 1 [file DataSheet1.ZIP › Data sources/Figure 2C, 2D, S3-IF/Figure 2C,D-Caco2/1-ZO-1/C. Butyricum/C.buty-ZO1.jpg]

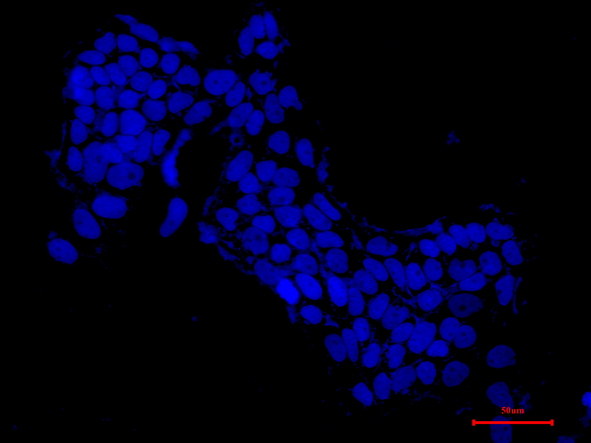

Supplement: Supplementary file 1 [file DataSheet1.ZIP › Data sources/Figure 2C, 2D, S3-IF/Figure 2C,D-Caco2/1-ZO-1/Control/con-DAPI.jpg]

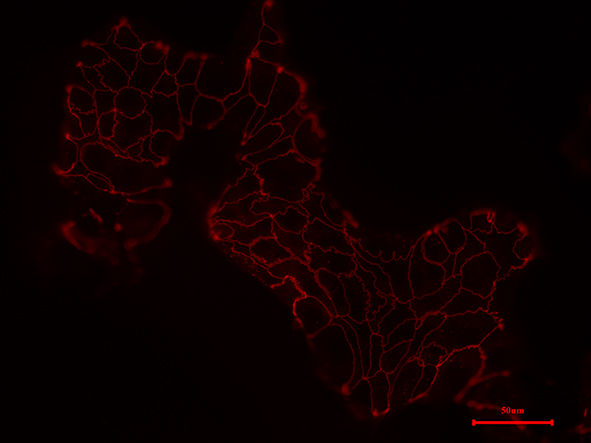

Supplement: Supplementary file 1 [file DataSheet1.ZIP › Data sources/Figure 2C, 2D, S3-IF/Figure 2C,D-Caco2/1-ZO-1/Control/con-ZO1.jpg]

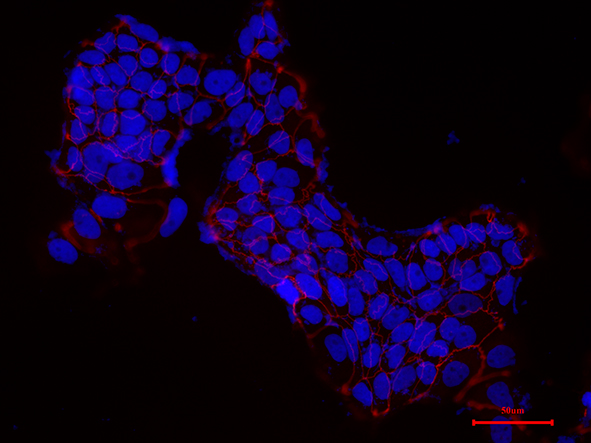

Supplement: Supplementary file 1 [file DataSheet1.ZIP › Data sources/Figure 2C, 2D, S3-IF/Figure 2C,D-Caco2/1-ZO-1/Control/merge.jpg]

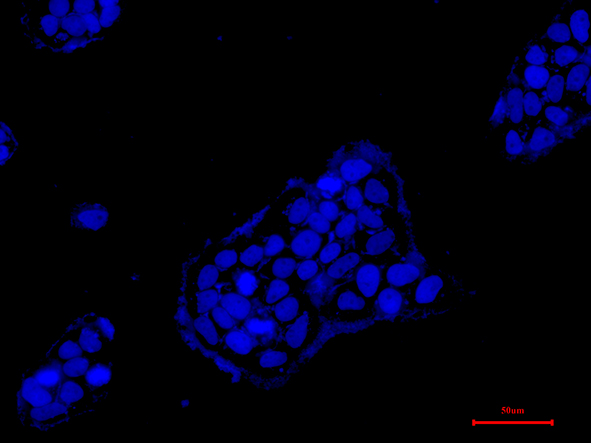

Supplement: Supplementary file 1 [file DataSheet1.ZIP › Data sources/Figure 2C, 2D, S3-IF/Figure 2C,D-Caco2/1-ZO-1/Butyrate/buty-DAPI.jpg]

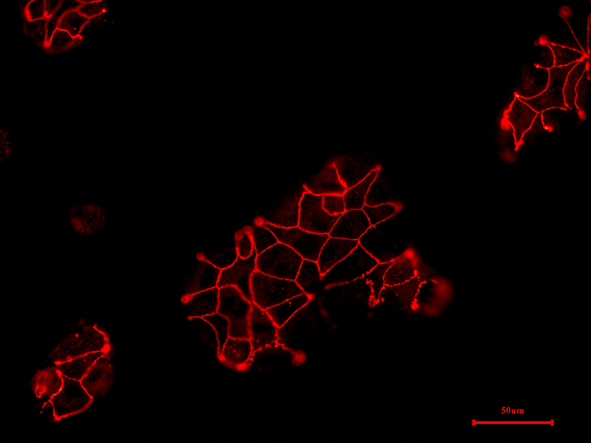

Supplement: Supplementary file 1 [file DataSheet1.ZIP › Data sources/Figure 2C, 2D, S3-IF/Figure 2C,D-Caco2/1-ZO-1/Butyrate/buty-ZO1.jpg]

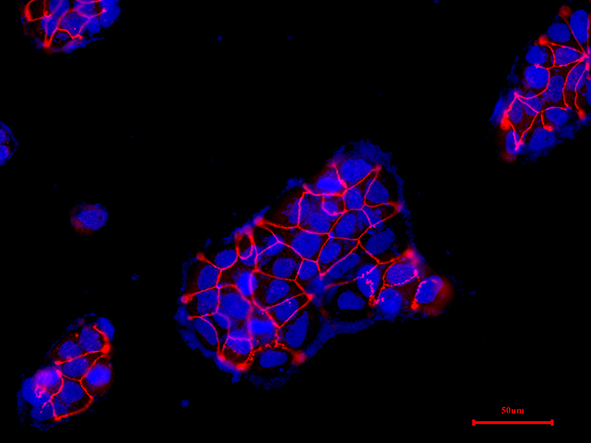

Supplement: Supplementary file 1 [file DataSheet1.ZIP › Data sources/Figure 2C, 2D, S3-IF/Figure 2C,D-Caco2/1-ZO-1/Butyrate/merge.jpg]

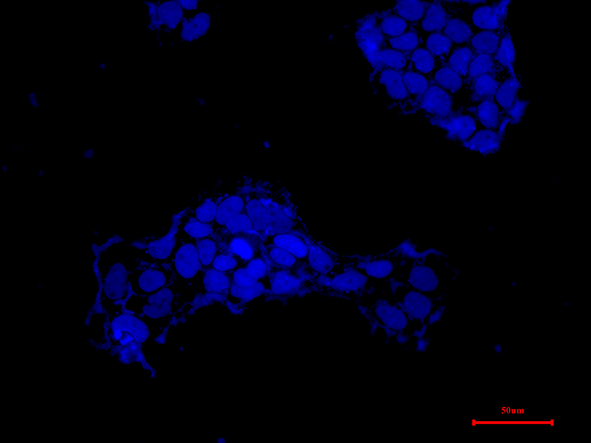

Supplement: Supplementary file 1 [file DataSheet1.ZIP › Data sources/Figure 2C, 2D, S3-IF/Figure 2C,D-Caco2/4-Occludin/CON+C.buty/DAPI-2.jpg]

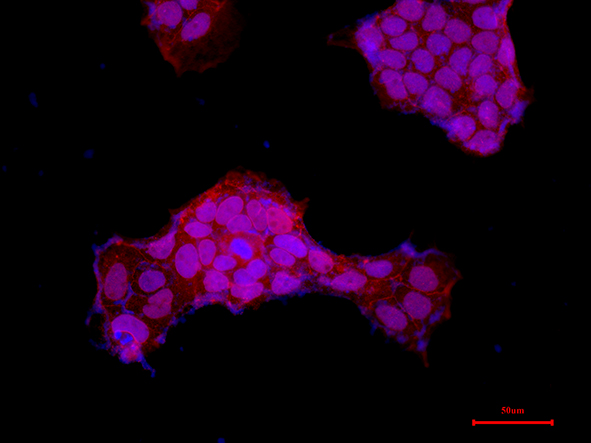

Supplement: Supplementary file 1 [file DataSheet1.ZIP › Data sources/Figure 2C, 2D, S3-IF/Figure 2C,D-Caco2/4-Occludin/CON+C.buty/10.jpg]

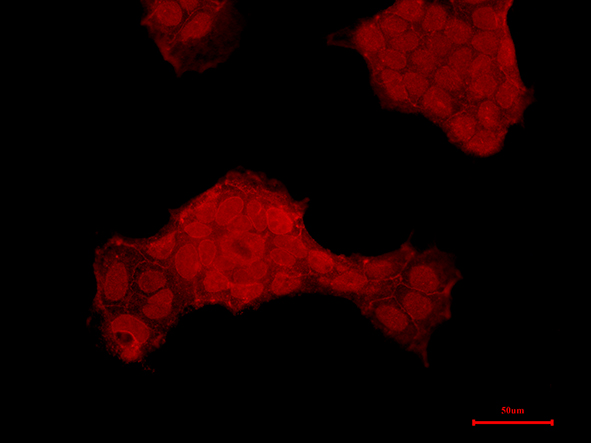

Supplement: Supplementary file 1 [file DataSheet1.ZIP › Data sources/Figure 2C, 2D, S3-IF/Figure 2C,D-Caco2/4-Occludin/CON+C.buty/occ-2.jpg]

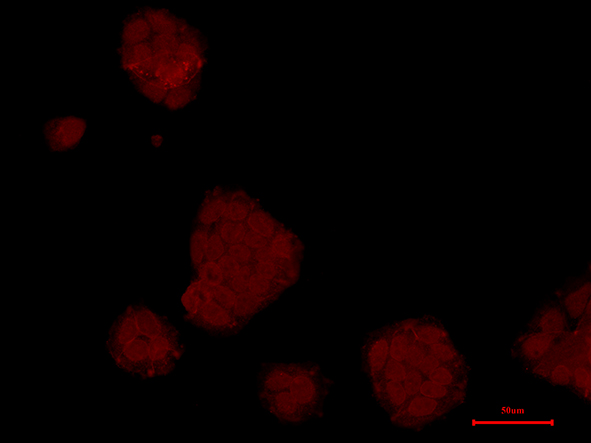

Supplement: Supplementary file 1 [file DataSheet1.ZIP › Data sources/Figure 2C, 2D, S3-IF/Figure 2C,D-Caco2/4-Occludin/C0N/con-occ-3.jpg]

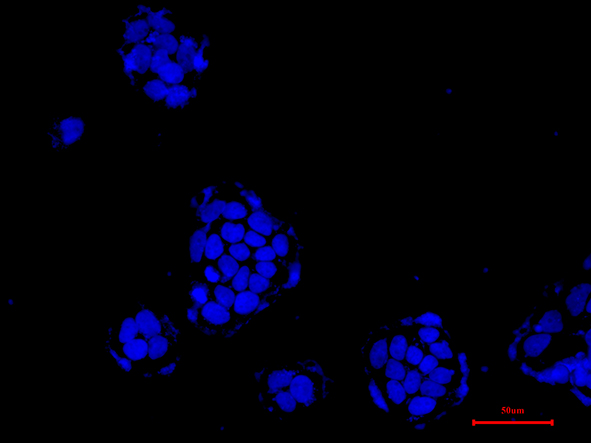

Supplement: Supplementary file 1 [file DataSheet1.ZIP › Data sources/Figure 2C, 2D, S3-IF/Figure 2C,D-Caco2/4-Occludin/C0N/con-DAPI-3.jpg]

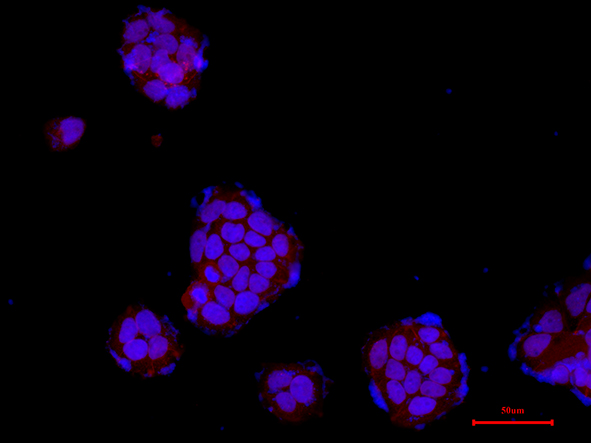

Supplement: Supplementary file 1 [file DataSheet1.ZIP › Data sources/Figure 2C, 2D, S3-IF/Figure 2C,D-Caco2/4-Occludin/C0N/3.jpg]

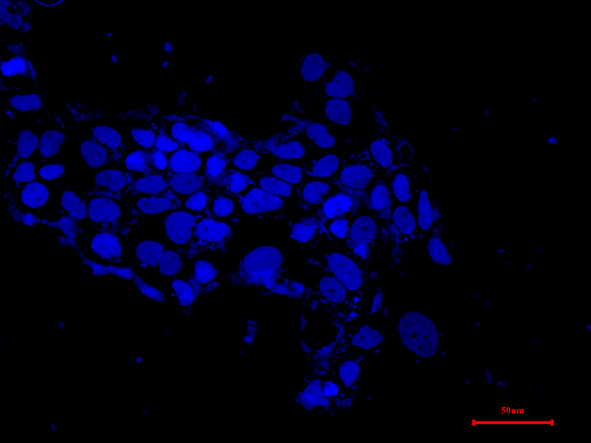

Supplement: Supplementary file 1 [file DataSheet1.ZIP › Data sources/Figure 2C, 2D, S3-IF/Figure 2C,D-Caco2/4-Occludin/CON+buty/DAPI-4.jpg]

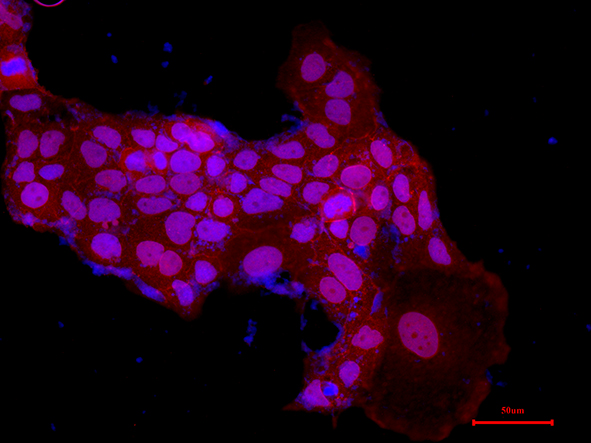

Supplement: Supplementary file 1 [file DataSheet1.ZIP › Data sources/Figure 2C, 2D, S3-IF/Figure 2C,D-Caco2/4-Occludin/CON+buty/8.jpg]

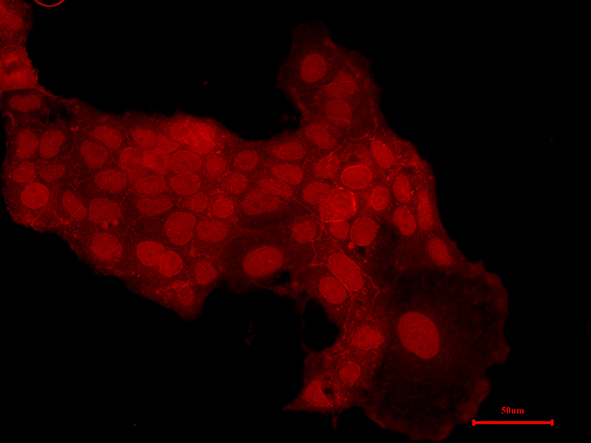

Supplement: Supplementary file 1 [file DataSheet1.ZIP › Data sources/Figure 2C, 2D, S3-IF/Figure 2C,D-Caco2/4-Occludin/CON+buty/occ-4.jpg]

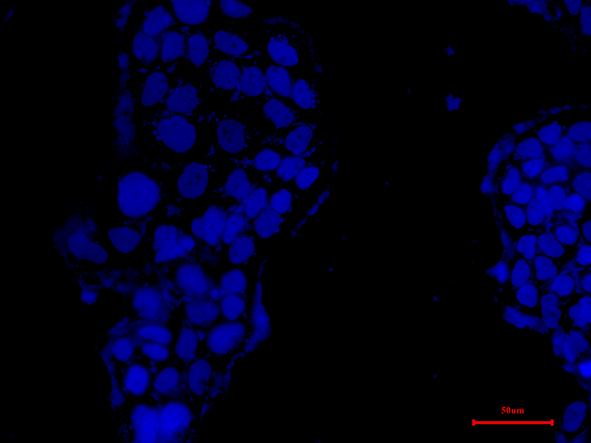

Supplement: Supplementary file 1 [file DataSheet1.ZIP › Data sources/Figure 2C, 2D, S3-IF/Figure 2C,D-Caco2/2-Claudin3/CON+C.buty/DAPI-2.jpg]

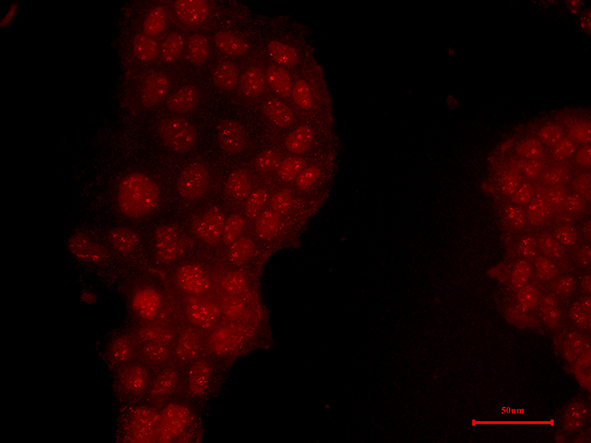

Supplement: Supplementary file 1 [file DataSheet1.ZIP › Data sources/Figure 2C, 2D, S3-IF/Figure 2C,D-Caco2/2-Claudin3/CON+C.buty/c3-2.jpg]

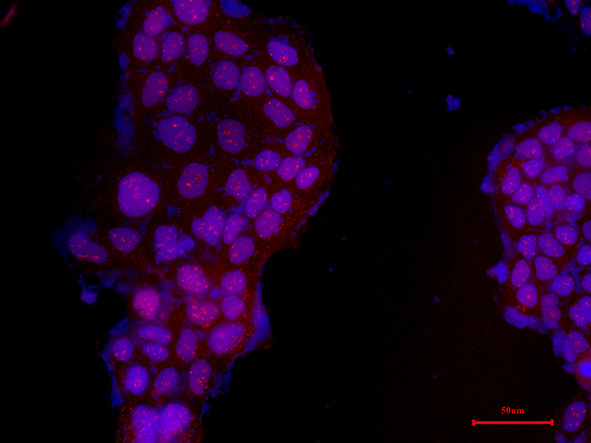

Supplement: Supplementary file 1 [file DataSheet1.ZIP › Data sources/Figure 2C, 2D, S3-IF/Figure 2C,D-Caco2/2-Claudin3/CON+C.buty/c3-2-1.jpg]

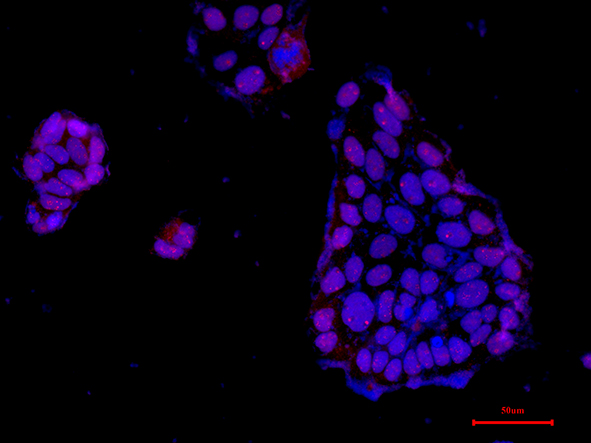

Supplement: Supplementary file 1 [file DataSheet1.ZIP › Data sources/Figure 2C, 2D, S3-IF/Figure 2C,D-Caco2/2-Claudin3/C0N/con-c3-2-1.jpg]

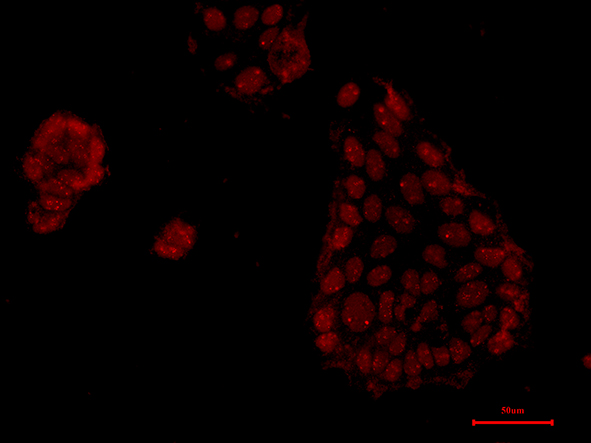

Supplement: Supplementary file 1 [file DataSheet1.ZIP › Data sources/Figure 2C, 2D, S3-IF/Figure 2C,D-Caco2/2-Claudin3/C0N/con-c3-2.jpg]

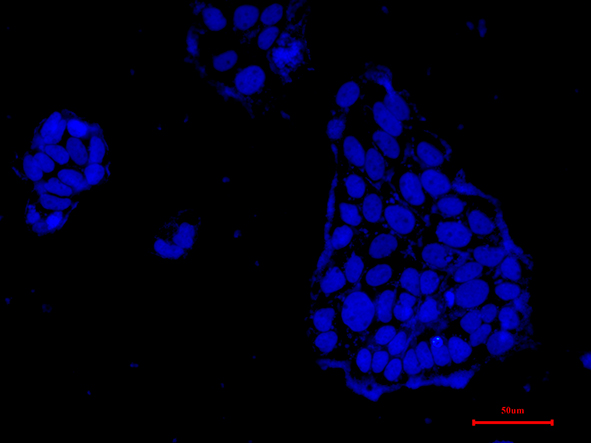

Supplement: Supplementary file 1 [file DataSheet1.ZIP › Data sources/Figure 2C, 2D, S3-IF/Figure 2C,D-Caco2/2-Claudin3/C0N/con-DAPI-2.jpg]

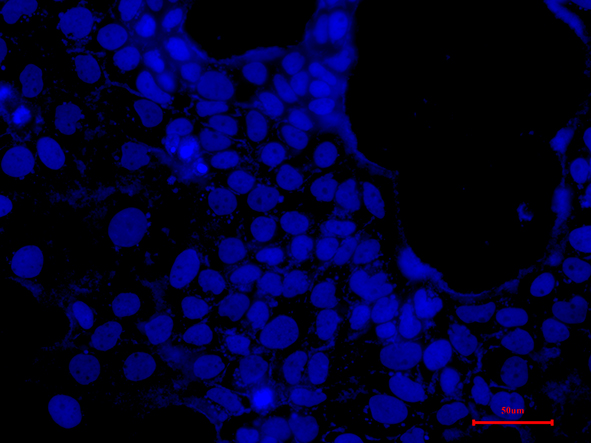

Supplement: Supplementary file 1 [file DataSheet1.ZIP › Data sources/Figure 2C, 2D, S3-IF/Figure 2C,D-Caco2/2-Claudin3/CON+buty/DAPI-2.jpg]

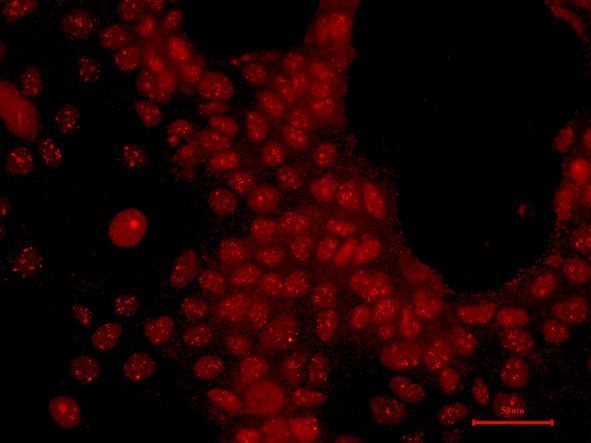

Supplement: Supplementary file 1 [file DataSheet1.ZIP › Data sources/Figure 2C, 2D, S3-IF/Figure 2C,D-Caco2/2-Claudin3/CON+buty/c3-2.jpg]

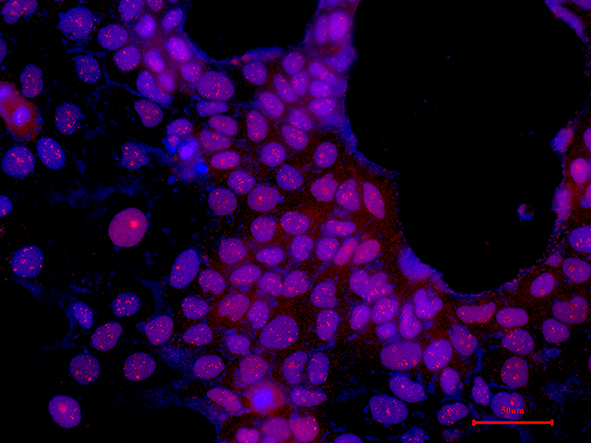

Supplement: Supplementary file 1 [file DataSheet1.ZIP › Data sources/Figure 2C, 2D, S3-IF/Figure 2C,D-Caco2/2-Claudin3/CON+buty/c3-2-1.jpg]
